# Additional Sarasinosides from the Marine Sponge *Melophlus sarasinorum* Collected from the Bismarck Sea

**DOI:** 10.1021/acs.jnatprod.3c01045

**Published:** 2023-11-30

**Authors:** Shauna O’Brien, Rodney Lacret, Maggie M. Reddy, Laurence K. Jennings, Pilar Sánchez, Fernando Reyes, Augustine Mungkaje, Kevin Calabro, Olivier P. Thomas

**Affiliations:** † School of Biological and Chemical Sciences, Ryan Institute, University of Galway, H91TK33 Galway, Ireland; ‡ BioLab, Instituto Universitario de Bio-Orgánica Antonio González (IUBO-AG), Universidad de La Laguna, Avenida Astrofísico Francisco Sánchez 2, 38206 La Laguna, Spain; § Departamento de Medicina Física y Farmacología, Facultad de Farmacia, Universidad de La Laguna, 38200 La Laguna, Tenerife, Spain; ∥ Department of Biological Sciences, University of Cape Town, Private Bag X3, Rondebosch 7701, South Africa; ⊥ Fundación MEDINA, Centro de Excelencia en Investigación de Medicamentos Innovadores en Andalucía, Avenida del Conocimiento 34, Parque Tecnologico de Ciencias de la Salud, E18016, Armilla, Granada, Spain; # Biological Sciences, University of Papua New Guinea, P.O Box 320, University 134, National Capital District, Port Moresby, Papua New Guinea

## Abstract

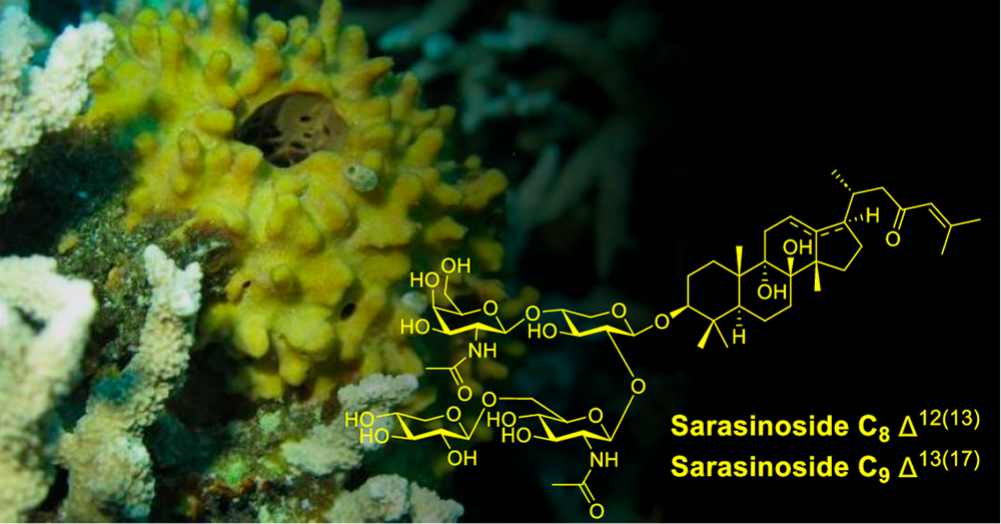

In our continuing efforts to describe the biological and chemical diversity of sponges from Kimbe Bay, Papua New Guinea, the known 30-norlanostane saponin sarasinoside C_1_ (**1**) was identified along with six new analogues named sarasinosides C_4_, C_5_, C_6_, C_7_, C_8_, and C_9_ (**2**–**7**) from the sponge *Melophlus sarasinorum*. The structures of the new compounds were elucidated by analysis of 1D and 2D NMR and HRMS data, as well as comparison with literature data. All new compounds are characterized by the same tetraose moiety, β-d-Xyl*p*-(1→6)-β-d-GlcNAc*p*-(1→2)-[β-d-GalNAc*p*-(1→4)]-β-d-Xyl*p*, as described previously for sarasinoside C_1_, but differed in their aglycone moieties. When comparing NMR data of sarasinoside C_8_ with those of known analogues, a misassignment was identified in the configuration of the C-8/C-9 diol for the previously described sarasinoside R (**8**), and it has been corrected here using a combination of ROESY analysis and molecular modeling.

Papua New Guinea (PNG), situated in the Coral Triangle in the western Pacific, is an area richly diverse in marine biodiversity and has the highest diversity of sponges in the world,^1^ which has in turn resulted in high chemical diversity and enormous potential for marine biodiscovery. The *Tara* Pacific expedition (2016–2018) offered a rare opportunity to study the biological and chemical diversity of sponges in Kimbe Bay, which is located in the north of PNG in the Bismarck Sea.^2^ The astrophorid sponge *Melophlus sarasinorum* was selected for further chemical investigation owing to its diverse chemical profile in our initial screening program and potential biological activity. This species was first described by Thiele in 1899 from the Celebes Sea (Sulawesi Sea).^3^*M. sarasinorum* is conspicuous in shallow-water reefs in PNG and has been widely recorded throughout much of the western Pacific Ocean. Species of the suborder Astrophorina are particularly challenging in terms of systematic classification, as morphologically diagnostic characters are not always supported by DNA.^4^ As such, the family to which *M. sarasinorum* belongs is still unclear, with morphological analyses affirming its position within the Ancorinidae^4^ and DNA placing it within the Geodiidae.^5^ Regardless of the classification scheme, the families Ancorinidae and Geodiidae remain non-monophyletic, which indicates that a more comprehensive revision of the order is needed. Until recently, the genus *Melophlus* accommodated three species. The type species is *M. sarasinorum* along with *M. ruber* and *M. hajdui.* However, a recent taxonomic study transferred the latter two species to *Stellettinopsis* based on morphological grounds. Triaenes are a morphologically diagnostic feature of *Stellettinopsis* that was detected in both *M. ruber* and *M. hajdui*. The presence of triaenes, however, can be easily missed, and therefore some authors argue that skeletal organization and microsclere geometry should also be considered.^6^ Nevertheless, these recent taxonomic changes make *M. sarasinorum* a monotypic species, which means that it is the only species in the genus *Melophlus* and therefore distantly related to other astrophorid sponges.

A review of the literature on marine natural products isolated from *M. sarasinorum*, including various misapplied names, indicated a major occurrence of saponins. In the phylum Porifera, both triterpenoid and steroid saponins have been found, although triterpenoid aglycone units are more common. Four families of saponins characterized by a lanostane triterpenoid skeleton have been reported thus far from various marine sponges such as erylosides from species in the genus *Erylus*;^7−10^ ulososides from *Ulosa* sp.^11^ and *Ectyoplasia ferox*,^12^ urabosides from *Ectyoplasia ferox*,^12^ and sarasinosides from different species.^13,14^ Sarasinosides are characterized by an aglycone with a 30-norlanostane core skeleton and a 23-keto-Δ^24(25)^ side-chain, with structural variations occurring in the oxidation patterns and migration of unsaturation in the triterpenoid unit.^15^ Sarasinoside A_1_ was described as the first member of this family of compounds in 1987 and 1988 from two specimens of the sponge *M. sarasinorum* collected from Palau and Guam, respectively.^14,16,17^ Eight new derivatives, sarasinosides A_2–3_, B_1–3_, and C_1–3_, characterized by three distinct carbohydrate units of five, five, and four sugar residues, respectively, were reported from the sponge collected from Palau.^14^ Additional sarasinosides D–G, with five sugar residues, were later found in a specimen of *M. sarasinorum* collected from the Solomon Islands.^18^ In 2000, four new derivatives, sarasinosides H_1–2_ and I_1–2_, again with five sugar residues, were isolated from a sponge referred to as *M. isis*, a synonym of *M. saranisorum*.^19^ The structures of the most recently reported sarasinosides J–R are also characterized by five sugar residues and different aglycones.^20,21^ Previous studies on the biological activities of sarasinosides showed that sarasinosides with a common 8(9)-double bond or 7(8),9(11)-diene systems possess strong or moderate cytotoxic activities against tumor cell lines, yeast, and fertilized eggs of starfish and ichthyotoxicity.^15^ Herein, we report the structures and biological activities of six additional sarasinosides, named sarasinosides C_4_ (**2**), C_5_ (**3**), C_6_ (**4**), C_7_ (**5**), C_8_ (**6**), and C_9_ (**7**), together with the known sarasinoside C_1_ (**1**) from a specimen collected in Kimbe Bay. They add to the growing reports of sarasinosides from *M. saranisorum* from different regions of the Coral Triangle.^13,14^ Their structures were deduced through high-resolution mass spectrometry and 1D and 2D NMR, and all feature a β-d-Xyl*p*-(1→6)-β-d-GlcNAc*p*-(1→2)-[β-d-GalNAc*p*-(1→4)]-β-d-Xyl*p* carbohydrate moiety and a 24,25-unsaturated-23-keto side-chain characteristic of the sarasinoside C series. Each of the newly identified metabolites shows variations in the oxidation patterns of the aglycone. Upon review of the literature for sarasinoside C_8_ (**6**), a configurational misassignment of the previously reported sarasinoside R (**8**) was identified, and a corrected structure is proposed here.^21^

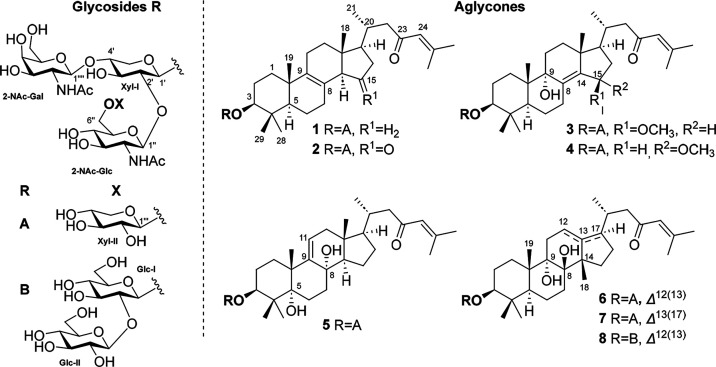



## Results and Discussion

The freeze-dried sponge material (78.7 g) was extracted three times with 1:1 CH_3_OH/CH_2_Cl_2_. The resulting extract (8.7 g) was fractionated using C-18 vacuum liquid chromatography with solvents of decreasing polarity. Fractions eluted with CH_3_OH/H_2_O (3:1) and CH_3_OH were purified by using HPLC to yield sarasinosides **1**–**7**.

The major metabolite of the MeOH fraction, sarasinoside C_1_ (**1**), was isolated as a yellowish, amorphous solid. Its molecular formula of C_55_H_87_N_2_O_20_ was determined by (+)-HRESIMS with a protonated adduct at *m*/*z* 1097.6000 [M + H]^+^. The structure was elucidated by a combination of 1D and 2D NMR analyses (Supporting Information S2 and S3) and comparison with data from the literature.^13^ In the original reports of the compound in 1987 and 1991, Kitagawa and colleagues reported only part of the NMR data for sarasinoside C_1_ in a mixture of pyridine-*d*_5_/D_2_O. Similar signals were found for **1** when the NMR was performed in the same solvent. Here, we also provide the full assignment of compound **1** in CD_3_OD that enables easy comparison to other analogues. For the aglycone component, the characteristic side-chain of a C-24/C-25 unsaturated ketone at C-23 of sarasinosides was evidenced with signals at δ_H_ 2.09 (H-22b), 2.52 (dd, H-22a), δ_C_ 52.4 (C-22), δ_C_ 204.0 (C-23), δ_H_ 6.16 (br s, H-24), δ_C_ 125.2 (C-24), δ_C_ 157.0 (C-25), δ_H_ 1.91 (s, 3H, H-26), δ_C_ 27.7 (C-26), 2.12 (s, 3H, H-27), and δ_C_ 20.9 (C-27). For the carbohydrate component, characteristic signals were identified for the four anomeric protons at δ_H_ 4.35 (d, *J* = 7.0 Hz, H-1′), 4.42 (d, *J* = 7.5 Hz, H-1‴), 4.47 (d, *J* = 8.0 Hz, H-1⁗), and 4.88 (d, *J* = 7.5 Hz, H-1″) and carbons at δ_C_ 102.1 (C-1″), 102.4 (C-1⁗), 105.2 (C-1‴), and 105.7 (C-1′) (Table 1), and they were unchanged for all sarasinosides C_1_ and C_4_–C_9_ reported herein. As expected from previous data in this family, changes between compounds occur around the C- and D-rings, having different oxidation patterns.

**Table 1:** NMR Data for the Glycoside of **1** in CD_3_OD (^1^H NMR 500 MHz and ^13^C NMR 125 MHz)

no.	δ_C_	δ_H_, mult. (*J* in Hz)
β-d-xylose		
1′	105.7, CH	4.35, d (7.5)
2′	79.4, CH	3.68,^a^
3′	77.1, CH	3.54,^a^
4′	79.4, CH	3.65,^a^
5′	64.0, CH_2_	3.18,^a^
		3.87,^a^
β-d-2NAc-glucosamine		
1″	102.1, CH	4.88, d (7.5)
2″	58.0, CH	3.66,^a^
3″	76.7, CH	3.43,^a^
4″	72.5, CH	3.25, t (9.1)
5″	77.1, CH	3.42,^a^
6″	69.9, CH_2_	3.68, dd (11.5, 6.5)
		4.10, dd (11.5, 1.8)
N-CO	174.0, C	
CO-CH_**3**_	23.0, CH_3_	1.99, s
β-d-Xylose		
1‴	105.2, CH	4.42, d (7.5)
2‴	75.1, CH	3.20, dd (9.0, 7.4)
3‴	77.6, CH	3.36, t (9.0)
4‴	71.2, CH	3.48, ddd (10.1, 8.7, 5.3)
5‴	66.9, CH_2_	3.24, dd (11.6, 10.1)
		3.82, d (11.6)
β-d-2NAc-galactosamine		
1⁗	102.4, CH	4.47, d (8.0)
2⁗	54.4, CH	3.89, dd (10.7, 8.3)
3⁗	72.8, CH	3.62,^a^
4⁗	69.6, CH	3.82,^a^
5⁗	77.2, CH	3.55,^a^
6⁗	62.7, CH_2_	3.73, dd (11.6, 4.5)
		3.81, dd (11.5, 7.5)
N-CO	174.1, C	
CO-CH_**3**_	23.0, CH_3_	1.99, s

a Overlapping signals.

Sarasinoside C_4_ (**2**) was isolated as a white amorphous solid, with (+)-HRESIMS analysis showing a [M + H]^+^ ion peak at *m*/*z* 1111.5800, consistent with the molecular formula C_55_H_87_N_2_O_21_. An initial inspection of the ^13^C NMR spectrum of the aglycone signals of **2** and comparison with those of **1** (Tables 2 and S5) revealed the presence of an additional carbonyl signal at δ_C_ 219.5. This signal was located at position C-15 in the D-ring of the aglycon after interpretation of key HMBC H-14, H_2_-16/C-15 correlations. Due to very similar chemical shifts to those of **1**, we deduced identical relative configurations for the aglycone of **2**. However, an aglycone with an unsaturation between C-8/C-9, a ketone at C-15, and a saturated carbon at C-14 is quite unusual among marine saponins, and therefore the configuration at C-14 needed additional confirmation. These features were only found in pandarosides E and F isolated from the Caribbean sponge *Pandaros acanthifolium*.^22,23^ As the β orientation of H-14 was demonstrated to be unusual in these saponins, we focused on the determination of the configuration at this position for **2**. Despite very close chemical shifts, an ROE correlation was clearly observed between H-14 and H-2α. Additionally, a chemical shift of δ_C_ 58.8 was observed for C-14 of the pandarosides, while δ_C_ 69.9 was observed for the same carbon in **2**, therefore suggesting the opposite and more common α orientation of H-14 in **2**. The value of the specific rotation was found to be extremely low for this compound, as for other compounds in the series. Higher amounts of these compounds are needed to make the measurement more accurate.

**Table 2:** ^1^H and ^13^C NMR Resonances for the Aglycones of **2**–**4** in CD_3_OD (^1^H = 600 MHz and ^13^C = 150 MHz)

		**2**		**3**		**4**	
no.		δ_C_	δ_H_, mult. (*J* in Hz)	δ_C_	δ_H_, mult. (*J* in Hz)	δ_C_	δ_H_, mult. (*J* in Hz)
1	α	35.8, CH_2_	1.18, t (7.1)	31.4, CH_2_	1.39,^a^	31.4, CH_2_	1.40,^a^
	β		1.80,^a^		1.71,^a^		1.71, td (14.0, 3.7)^a^
2	α	27.6, CH_2_	1.86, dt (13.1, 3.8)	27.4, CH_2_	1.83, dd (13.0, 3.0)^a^	27.4, CH_2_	1.82,^a^
	β		1.74,^a^		1.67,^a^		1.67,^a^
3	α	91.1, CH	3.09, dd (11.7, 4.1)	91.1, CH	3.12, dd (11.3, 3.8)	91.1, CH	3.12, dd (11.6, 4.1)
4		40.4, C		40.5, C		40.4, C	
5	α	52.5, CH	1.04, dd (12.7, 1.6)	45.5, CH	1.92,^a^	45.4, CH	1.96,^a^
6	α	19.4, CH_2_	1.70,^a^	22.9, CH_2_	1.64, ddd (13.1, 5.1, 2.8)	22.7, CH_2_	1.60,^a^
	β		1.46,^a^		1.35, qd (12.5, 4.8)		1.31,^a^
7	α	31.5, CH_2_	2.03,^a^	26.3, CH_2_	2.16,^a^	29.1, CH_2_	2.29, m
	β		1.94,^a^		2.54, br d (12.0)		
8		122.3, C		136.5, C		138.1, C	
9		142.0, C		76.2, C		76.3, C	
10		39.3, C		42.4, C		42.1, C	
11	α	21.9, CH_2_	2.09,^a^	28.3, CH_2_	1.46,^a^	28.7, CH_2_	1.48,^a^
	β				1.92,^a^		2.03,^a^
12	α	33.0, CH_2_	1.29, m	35.2, CH_2_	1.45,^a^	34.9, CH_2_	1.52,^a^
	β		1.94,^a^		1.78, dd (9.3, 3.5)		1.83,^a^
13		42.0, C		44.0, C		44.5, C	
14	α	67.0, CH	2.29, br s	144.1, C		147.3, C	
15	α	219.5, C		79.6, C	4.22, dd (7.7, 5.5)	80.6, C	4.22, d (5.9)
	β						
16	α	41.6, CH_2_	2.40, dd (18.5, 8.5)	36.4, CH_2_	2.28, dt (13.2, 7.7)	34.4, CH_2_	1.93,^a^
	β		2.01, br d (18.5)		1.42,^a^		1.55,^a^
17	α	43.2, CH	2.11, br d (8.0)	55.5, CH	1.17, ddd (12.2, 9.0, 7.7)	55.4, CH	1.44,^a^
18		22.7, CH_3_	1.01, s	18.4, CH_3_	0.93, s	18.7, CH_3_	0.89, s
19		19.7, CH_3_	1.02, s	17.9, CH_3_	1.00, s	18.7, CH_3_	0.94, s
20		32.8, CH	2.15,^a^	33.2, CH	2.13,^a^	32.2, CH	2.09,^a^
21		20.3, CH_3_	1.00, d (6.0)	20.5, CH_3_	0.95, d (6.3)	20.5, CH_3_	0.97, d (6.5)
22		51.4, CH_2_	2.22, dd (15.0, 9.2)	52.0, CH_2_	2.16,^a^	52.1, CH_2_	2.21, dd (15.2, 9.3)
			2.49, dd (15.0, 2.4)		2.59, dd (15.1, 3.2)		2.60, dd (15.2. 4.0)
23		203.0, C		203.6, C		203.6, C	
24		125.3, CH	6.18, br s	125.2, CH	6.19, br s	125.3, CH	6.20, br s
25		157.3, C		157.1, C		157.1, C	
26		27.7, CH_3_	1.91, br s	27.7, CH_3_	1.92, d (1.3)	27.7, CH_3_	1.92, br s
27		20.9, CH_3_	2.13, br s	20.9, CH_3_	2.12, d (1.3)	20.9, CH_3_	2.13, br s
28		17.2, CH_3_	0.90, s	17.3, CH_3_	0.93, s	17.3, CH_3_	0.93, s
29		28.5, CH_3_	1.08, s	29.1, CH_3_	1.11, br s	29.2, CH_3_	1.11, s
15-OCH_**3**_	α			56.1, CH_3_	3.29, br s^a^	56.4, CH_3_	3.30, s^a^
	β						

a Overlapping signals.

Sarasinoside C_5_ (**3**) and sarasinoside C_6_ (**4**) were both isolated as amorphous white solids and with the same molecular formulas of C_56_H_90_N_2_O_22_, which were deduced from the peaks of the protonated adducts at *m*/*z* 1143.6039 and 1143.6059 [M + H]^+^ in their respective (+)-HRESIMS spectra. The planar structures of both molecules were identical according to the 1D and 2D NMR data (Table 1), which showed a migration of the internal double bond from C-8/C-9 in **1** and **2** to C-8/C-14 in **3** and **4**, as demonstrated by the absence of the signal corresponding to methane H-14 in **3** and **4**. Simultaneously, the position at C-9 was substituted by a hydroxyl group, as evidenced by the presence of a new oxygenated and nonprotonated carbon at δ_C_ 76.2 (C-9). Finally, the ketone at C-15 in **2** was replaced by a new oxygenated methine in both compounds **3** and **4** with a signal at δ_H_ 4.22 (H-15). This chemical shift was indicative of the presence of a methoxy group at C-15 for both **3** and **4**, which suggested that both compounds were diastereoisomers. Comparing with similar saponins described in the literature, **3** and **4** were found to be structurally similar analogues of sarasinosides H_2_ and I_2_ reported from *M. sarasinorum* (as *M. isis*) in 2000.^19^ The aglycone for **3** is found to be the same as the one for sarasinoside H_2_ with the methoxy group in the β-position, while the aglycone of **4** was identical to the one of I_2_ and the methoxy group in the α-position. The only difference between sarasinosides C_5_ (**3**) and C_6_ (**4**) and sarasinosides H_2_ and I_2_ is the absence of a glucose unit in the carbohydrate component of the molecules. Importantly, sarasinosides H_2_ and I_2_ were identified in *M. sarasinorum* (as *M. isis*) collected from Guam, and this variation in the sugar unit could likely be linked to the environment.

Sarasinoside C_7_ (**5**) was isolated as a white amorphous solid, with (+)-HRESIMS analysis showing a [M – H_2_O + H]^+^ ion peak at *m*/*z* 1111.5824, consistent with the molecular formula C_55_H_88_N_2_O_22_. A new olefinic hydrogen at δ_H_ 5.05 (br t, *J* = 2.8 Hz, H-11) indicated the migration of the internal double bond to a trisubstituted position. The location of the double bond was assigned at C-9/C-11 through key H-7β, H-12β, and H_3_-19/C-9 and H-12β, H-14α/C-11 HMBC correlations. Two hydroxyl groups were located at C-5 and C-8 at δ_C_ 93.7 and 87.3 due to key H_2_-1, H-6α, H_3_-28, H_3_-29/C-5 and H-7β and H_2_-15/C-8 HMBC correlations. When searching for similar features in the sarasinoside series, we identified a nearly perfect match between all chemical shifts of **5** with those of sarasinoside O reported from *Lipastrotethya* sp.^21^ Observing very similar ROE values between all substituents, we also propose that these two hydroxyl groups are located on the α face of the triterpenoid aglycone. Here again, the only difference between sarasinoside C_7_ (**5**) and known sarasinoside O is the lack of the glucose terminal unit in **5**.

Sarasinoside C_8_ (**6**) was isolated as a white, amorphous solid. The molecular formula of C_55_H_88_N_2_O_22_ was determined by the (+)-HRESIMS ion peak [M + H]^+^ at *m*/*z* 1129.5866 indicative of an isomer of **5**. An initial inspection of the NMR data of **6** (Tables 3 and S6) showed similar signals to those in **5**, with variations evident by a difference in the olefinic proton resonance at δ_H_ 5.19 (d, *J* = 7.4 Hz, 1H, H-12). The location of the trisubstituted double bond at C-12/C-13 was confirmed through COSY and key H_3_-18, H-15, and H-16/C-13 and H-11α/C-12 and C-13 HMBC correlations. Interestingly, this implied that methyl C-18 had migrated from the usual position C-13 to C-14. This new position was confirmed through an additional key H_3_-18/C-8 HMBC correlation. Two nonprotonated and oxygenated carbons were again present in **6** but this time at different chemical shifts δ_C_ 69.8 (C-8) and δ_C_ 72.1 (C-9). Their locations at C-8 and C-9 were assigned through key H-6β, H-7α, H-11α, H_3_-18/C-8 and H-5α, H-7α, H-11α, H-12β, and H_3_-19/C-9 HMBC correlations.

**Table 3:** ^1^H and ^13^C NMR Resonances for the Aglycone Units of **5**–**7** in CD_3_OD (^1^H = 600 MHz and ^13^C = 150 MHz)

		**5**		**6**		**7**	
no.		δ_C_	δ_H_, mult. (*J* in Hz)	δ_C_	δ_H_, mult. (*J* in Hz)	δ_C_	δ_H_, mult. (*J* in Hz)
1	α	39.1, CH_2_	1.45, dt (13.7, 3.7)	33.7, CH_2_	1.35,^a^	33.6, CH_2_	1.29,^a^
	β		1.49, m		1.71,^a^		1.71,^a^
2	α	28.0, CH_2_	1.70,^a^	27.3, CH_2_	1.87,^a^	27.2, CH_2_	1.86, dd (13.4, 3.7)
	β		1.73,^a^		1.69,^a^		1.67,^a^
3	α	87.3, CH	3.46, m	90.6, CH	3.06, dd (11.7, 4.0)	90.6, CH	3.03, dd (11.7, 4.1)
4		41.6, C		40.2, C		40.1, C	
5	α	93.7, C		44.7, CH	1.48, dd (12.5, 1.5)	44.3, CH	1.39 dd (12.7, 1.2)
6	α	27.7, CH_2_	1.78, td (12.4, 4.0)	18.1, CH_2_	1.50,^a^	17.8, CH_2_	1.47, dd (12.6, 8.5)
	β		1.88, dd (12.0, 4.0)		1.33,^a^		1.31,^a^
7	α	28.9, CH_2_	1.71,^a^	24.7, CH2	1.69,^a^	24.5, CH_2_	1.68,^a^
	β		1.51, td (12.0, 4.0)		2.18, dd (14.9, 7.1)		2.05,^a^
8		87.3, C		69.8, C		70.5, C	
9		156.1, C		72.1, C		73.5, C	
10		45.8, C		37.3, C		38.0, C	
11	α	112.7, CH	5.05, br t (2.8)	25.1, CH_2_	2.38, dd (16.8, 7.3)	23.1, CH_2_	1.72,^a^
	β				2.48, d (16.8)		2.05,^a^
12	α	42.9, CH_2_	1.95,^a^	115.0, CH	5.19, d (7.4)	19.6, CH_2_	2.16,^a^
	β		2.23, dd (17.2, 4.1)				1.96,^a^
13		43.8, C		148.4, C		138.5, C	
14	α	52.3, CH	1.98,^a^	49.1, C		52.9, C	
15	α	21.5, CH_2_	1.62, dd (10.7, 5.3)	33.7, CH_2_	1.55, dd (11.5, 6.3)	33.9, CH_2_	1.59, dd (11.7, 7.1)
	β		1.69, dd (10.2, 2.7)				1.75, d (11.7)
16	α	29.5, CH_2_	1.37,^a^	25.5, CH_2_	1.69,^a^	28.2, CH_2_	2.18,^a^
	β		1.94,^a^		1.43,^a^		2.21,^a^
17	α	57.8, CH	1.34,^a^	50.2, CH	2.48, m	136.4, C	
18		15.3, CH_3_	0.76, s	22.0, CH_3_	1.03, s	22.1, CH_3_	1.01 s
19		23.4, CH_3_	1.27, s	18.1, CH_3_	1.15,^a^	17.8, CH3	1.09 s
20		34.2, CH	1.99,^a^	33.2, CH	2.25, m	30.6, CH	2.96, dd (15.0, 7.0)
21		19.6, CH_3_	0.92, d (6.5)	18.8, CH_3_	0.91, d (6.6)	19.6, CH_3_	1.00, d (6.9)
22		52.2, CH_2_	2.14, dd (15.2, 9.6)	48.3, CH_2_	2.14, dd (14.5, 10.7)	50.7, CH_2_	2.32 dd (14.0, 9.1)
			2.56, dd (15.2, 3.3)		2.45, dd (14.5, 2.8)		2.36, dd (14.0, 6.0)
23		203.8, C		203.8, C		203.0, C	
24		125.2, CH	6.18, br s	125.1, CH	6.14, br s	124.9, CH	6.15, br s
25		157.0, C		157.0, C		157.1, C	
26		27.6, CH_3_	1.91, s	27.6, CH_3_	1.90, s	27.6, CH_3_	1.89, br s
27		20.8, CH_3_	2.12, s	20.8, CH_3_	2.11, s	20.9, CH_3_	2.06, br s
28		18.8, CH_3_	1.02, s	17.0, CH_3_	0.92, s	16.9, CH_3_	0.89, s
29		21.7, CH_3_	1.17, s	29.1, CH_3_	1.06, s	28.6, CH_3_	1.02, s

a Overlapping signals.

Sarasinoside C_9_ (**7**) was isolated as a white amorphous solid, and its molecular formula C_55_H_88_N_2_O_22_ was determined by the (+)-HRESIMS ion peak [M – H_2_O + H]^+^ at *m*/*z* 1111.5758, indicating another isomer of **5** and **6**. The two hydroxyl groups were again present at C-8 and C-9 with very similar chemical shifts to those in **6**. Therefore, the assumption was made that the changes occurred on the position of the unsaturation, as the olefinic signal H-12 was absent in the ^1^H NMR spectrum of **7**. The migration of the double bond to Δ^13(17)^ was further assigned through the key H_3_-21/C-17 HMBC correlation. After the planar structures of **6** and **7** were established, we reviewed the literature for sarasinosides to find similarities to the reported metabolites in order to assign the configurations of both isomers.

**Figure 1:**
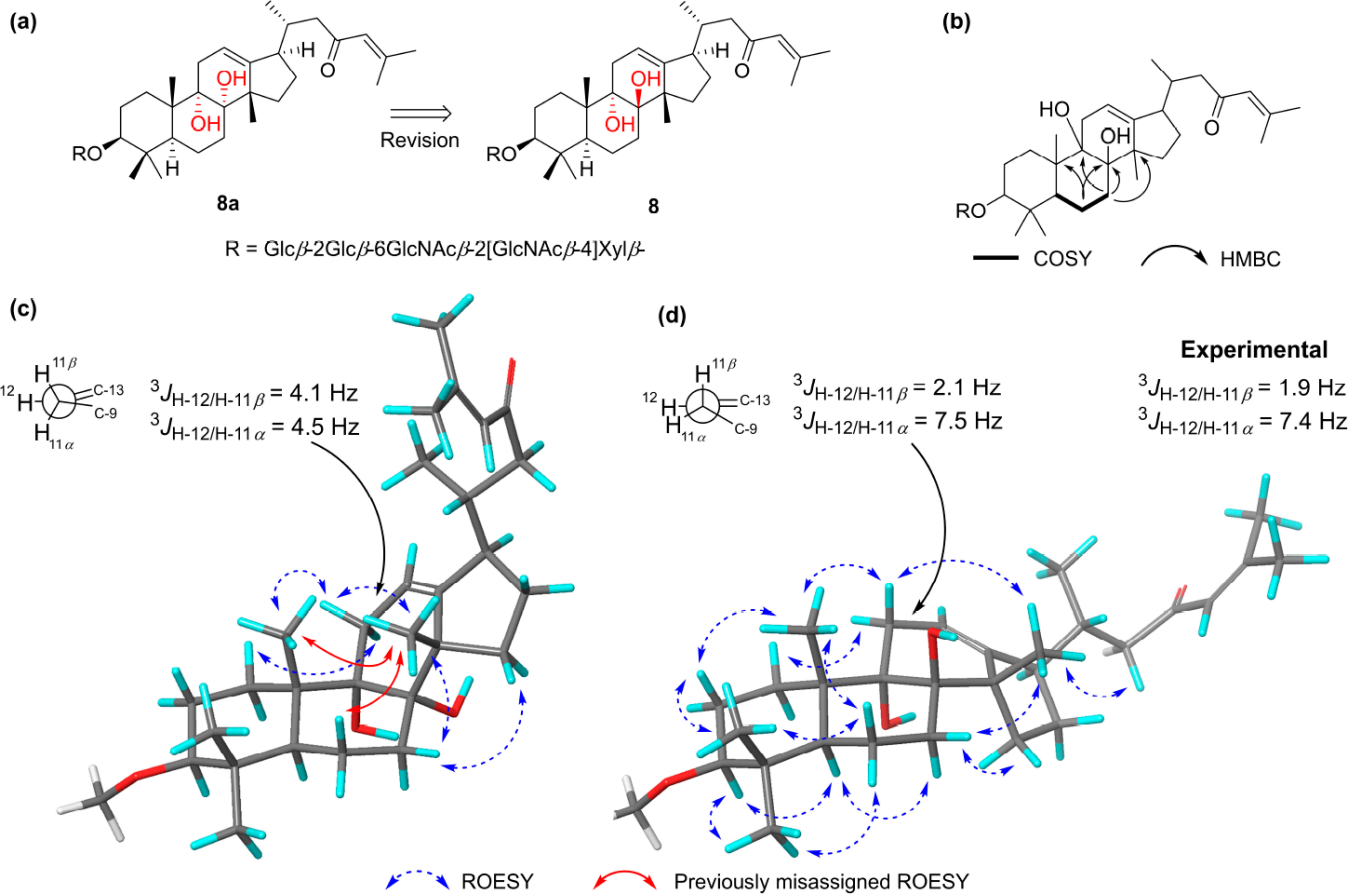
(a) The proposed structural revision of sarasinoside R (**8**). (b) Key COSY and HMBC data for the NMR reassignment of the aglycones of **6** and **8**. (c) DFT-optimized lowest energy conformers of the 8α,9α-*cis-*diol and (d) the 8β,9*α-trans*-diol. The Newman projections represent the C-11/C-12 bond geometries and the DFT-calculated spin–spin couplings of H-12 (exptl δ_H_ 5.19, dt, *J* = 7.4, 1.9 Hz).

Sarasinoside R (**8**) was previously reported with the same planar aglycone as **6**.^21^ The close similarity between the ^1^H and ^13^C NMR data for **6** and **8** also indicated the same configurations of the aglycone moiety. On closer inspection of the published data for **8**, it was evident that the NMR data contained a critical misassignment. The C-6 and C-7 methylenes had incorrectly been assigned as C-7 and C-6, respectively. This was evidenced by the lack of the COSY correlation from the axial H-5 proton to δ_H_ 2.18 or 1.69 (H-7) with the actual COSY correlation likely overlapping with the geminal correlation of δ_H_ 1.50 and 1.33 (H-6). Further evidence was present in the ROESY spectrum with large correlations from the axial C-28 methyl to the axial δ_H_ 1.33 (H-6β) and from the equatorial C-29 methyl to the equatorial δ_H_ 1.50 (H-6α). Finally, HMBC correlations from δ_H_ 2.18 (H-7α) to C-14 and from δ_H_ 1.69 (H-7β) to both C-8 and C-9 confirmed the incorrect previous assignment. Similar to sarasinoside R (**8**), the configuration of the β-axial C-19 methyl and α-axial H-5 in **6** could be assigned from the ROESY correlations between H-1α/H-3 and H-5, H-2β/H_3_-19 and H_3_-28 (previously assigned in **8** as H-6β/H-19 and H-6β/H-28), H-3/H-5, and H_3_-19/H_3_-28. In **8**, correlations between H-6β/H_3_-18, H-11β/H-18 and H-19, H-15α/H-17, H-15β and H-16β/H-18, and H_3_-18/H_3_-19 were previously used to assign the C-8 and C-9 diol in α-cis configuration and the C-14 methyl in a β configuration. While these correlations would all indicate the C-18 methyl is indeed on the β face of the molecule, only the H-6β/H_3_-18 and H_3_-18/H_3_-19 correlations indicate the α configuration of the two hydroxy groups.^21^ On reviewing the published supplementary ROESY data of sarasinoside R (**8**), these key correlations between δ_H_ 1.69 and 1.02 (H-7α/H_3_-18 misassigned as H-6β/H_3_-18) and δ_H_ 1.13 and 1.02 (H_3_-18/H_3_-19) were not observed. Additionally, in the ROESY data of the new compound **6**, these correlations including the correct H-6β/H_3_-18 (δ_H_ 1.33 and 1.03) and misassigned H-6β/H_3_-18 (δ_H_ 1.69 and 1.03, H-7α/H_3_-18) and δ_H_ 1.15 and 1.03 (H_3_-18/H_3_-19) were not evidenced, casting doubt on the configuration of the C-8/C-9 diol in both **6** and **8**. We then used a combination of ROESY analysis and molecular modeling to reinvestigate the aglycone structure to assign these configurations. First, a conformer search on the aglycone moiety for both the *trans*-8,9 and *cis*-8,9 configurations was performed including ROESY constraints and locking the conformation of the acyclic chain. This resulted in the generation of two primary conformations around the diol for each configuration. The structures were further optimized, and the energy of each was calculated using density functional theory (DFT) (Figure 1). Other than the clear lack of ROESY correlations between H_3_-18/H_3_-19 and H-6β/H_3_-18, further data indicated that the most likely configuration of the diol was the *trans*-8,9. The two modeled structures both contained a similar conformation of the first two rings, while the third ring contained significant differences. The intense ROESY correlation between δ_H_ 2.38 and 1.71 (H-1β/H-11α) indicated an equatorial position for both protons. Furthermore, the ^3^*J*_HH_ coupling between δ_H_ 5.19 and 2.48 was ≈2 Hz, indicating an ∼90-degree dihedral angle between H-12 and H-11β. Both the equatorial configuration of H-11α and the small coupling were only possible with the diol in a *trans* configuration. Furthermore, the intense ROESY correlation from δ_H_ 2.18 to 1.55 and 1.03 (H-7β/H_3_-18, H-7β/H-15β) indicated that all of these hydrogens were placed in close proximity as observed in the *trans*-diol configuration. As such, we assign the relative configuration of the aglycone of **6** as 3S*,5S*,8R*,9S*,10S*,14S*,17R*,20R* and propose the revision of the C-8 relative configuration of sarasinoside R (**8**). Compound **7** displayed similar NMR shifts for both the C-18 methyl and throughout ring B, indicating the same configuration of the diol. Furthermore, the similarities between the ROESY correlations of **6** and **7**, including between H-7β/H_3_-18 and H-7β/H-15, also indicated the same configuration of the C-8, C-9, C-10, and C-14 stereogenic centers.

Compounds **1**, **3**, and **4** were subjected to a range of cytotoxicity and antimicrobial assays. However, no significant activity was observed at the highest concentrations tested (Supporting Information S4). This is the first assessment of cytotoxicity and antimicrobial activity of sarasinoside C_1_ and sarasinosides with equivalent aglycone units to **3** and **4**, as sarasinosides H_2_ and I_2_ were not previously evaluated.^19^ The lack of activity for **3** and **4** is in agreement with the hypothesis that sarasinosides with Δ^8(9)^ unsaturation or a Δ^7(8),9(11)^ diene system show increased activity in comparison to Δ^8(14)^ unsaturation and highly oxygenated aglycone systems.^24^ Previous evaluations of sarasinosides for biological activity have reported cytotoxicity against K562 leukemia and A549 lung carcinoma cell lines (LC_50_ values between 7.4 and >100 μM). However, according to the current reference standards, they are considered inactive.^21^ Similarly, sarasinosides B_1_ and M_2_ were evaluated against Neuro-2a mouse neuroblastoma and HepG2 human Caucasian hepatocyte carcinoma cell lines, with M_2_ showing weak activity with IC_50_ values of 5.8 ± 0.3 and 5.9 ± 1.2 μM respectively.^25^

A large variety of saponins have been isolated from our specimen of *M. sarasinorum* collected from Kimbe Bay, Papua New Guinea, with sarasinoside C_1_ (**1**) being by far the major constituent. Interestingly, the specimen we collected provided the same carbohydrate motif for all isolated sarasinosides, C_4_ to C_9_. The variations were observed for the oxidation pattern of the 30-norlanostane core with an identical side-chain for all the aglycones. The aglycones of sarasinosides **2** and **7** have not been previously reported for this family. The other aglycones have already been described mainly from *M*. *sarasinorum* (as *M. isis*) collected from Guam, but also from other specimens of this species collected from Palau and West Sulawesi, with the saponins reported in those specimens containing an additional glucose unit attached to the end of the same tetraose glycoside.^19^ We first suspected that the loss of the fifth sugar unit could have occurred during the extraction and purification process or during sonication in MeOH. However, the first isolation of sarasinoside C_1_ was accompanied by the pentaose analogues sarasinosides A_1–3_ and B_1–3_, and the authors used an alternative extraction method without sonication, which would not favour this hypothesis. Furthermore, a recent investigation into the metabolome of *Melophlus* sponges found that the tetraose sarasinoside C_1_ (**1**) had a higher relative abundance in one group of *Melophlus* sponge in comparison to another using the same sample processing method, ruling out the possibility of the tetraose metabolite being an artifact.^26^ The biosynthetic gene cluster for sarasinosides was annotated from the sponge holobiont, indicating a γ-proteobacterial origin.^26^ The Mariana Islands are located in the marine province Tropical Northwestern Pacific, with Palau and Sulawesi in the western Coral Triangle and the Bismarck Sea in the eastern Coral Triangle. The variability in the chemical diversity of sarasinosides among the different specimens collected in different maritime regions could be explained well by the geographical variation linked to slight changes in microbial diversity. This assumption would therefore justify expanding marine biodiscovery of the same sponge species from different maritime regions, but of course, more data are needed to support this hypothesis.

In terms of chemotaxonomic relevance, the phylogenetic tree indicates that *Melophlus* is monotypic and closely related to the genus *Caminus* and more distantly related to two other genera, *Penares* and *Erylus*, within the family Erylinae. The only two studies on a species of the genus *Caminus* were conducted on *Caminus sphaeroconia* from the Caribbean Sea.^27,28^ Interestingly, the new caminosides isolated from this species are tetraose lipids corresponding to another type of amphiphilic molecule with a similar carbohydrate pattern. In this case, no steroid or triterpenoid saponins were identified, but these molecules could have a similar function. From the genus *Penares*, a large diversity of oxidized triterpenoids similar to those encountered in *Melophlus* have been identified and even some diose saponins.^29−32^ From the genus *Erylus*, both glycolipids and a vast diversity of triterpenoid saponins have been isolated, suggesting that some species of this genus might be closely related to the genus *Caminus* and others to *Melophlus* or *Penares*.^33,34^ Both glycosylated lipids and triterpenoids could therefore represent chemotaxonomic markers of the closely related genera *Melophlus*, *Penares*, *Caminus*, and *Erylus*. However, sponge species from other genera such as *Pandaros* have also been found to produce a large diversity of steroid saponins.^22^ Also, species of the genera *Ectyoplasia* and *Ulosa* produce triterpenoid saponins even though they belong to two different orders of sponges.^35,36^ Of course, this assertion depends on the correct identification of these sponges, which is yet to be done. If it were to be confirmed, this would show a broader occurrence of these saponins among sponge orders, which would be linked to their microbial symbionts rather than the host itself.

## Experimental Section

### General Experimental Procedures

UV and ECD data were obtained using a Chirascan V100 instrument with a 1.0 cm quartz cuvette. Optical rotations were recorded with an Autopol V polarimeter equipped with a 10 cm cell at 20 °C and at the Na D line at 589.3 nm. NMR experiments were performed on a 600 MHz spectrometer and a Varian Inova 500 MHz spectrometer. Chemical shifts were referenced to the residual solvent signals in ppm (CD_3_OD, at δ_H_ 3.31 and δ_C_ 49.00 ppm). High-resolution mass spectra were obtained with an Agilent 6540 Q-TOF mass spectrometer equipped with an Agilent 1290 UHPLC and autosampler. Preparative and semipreparative purifications were carried out on a Jasco LC-2000 series HPLC system. Optical rotations were recorded on a Unipol L1000 polarimeter at the sodium D-line (589.3 nm) with a 10 cm cell at 20 °C. IR spectra were recorded on a PerkinElmer Spectrum 400 FT-IR spectrometer (4000–650 cm^–1^).

### Animal Material

#### Sample Collection and Identification

The specimen (MBNUIG 380) identified as *Melophlus sarasinorum* studied here was collected as part of a larger assessment of the biodiversity of the sponges of Kimbe Bay, Papua New Guinea. A voucher specimen is preserved in 99% EtOH in our repository,^43^ and DNA is isolated from a tissue subsample of ca. 10 mg. The Qiagen DNeasy blood and tissue kit was used for DNA extraction and followed the manufacturer’s protocol, but with the addition of Proteinase K and slightly extended incubation periods. The DNA extract was diluted 1:10 before PCR. The mitochondrial, COI gene was amplified using previously published primers and thermal profiles. PCR reactions (25 μL) consisted of 12.5 μL of DreamTaq Hot Start Green PCR master mix, 0.1–0.5 μM of each primer, and 1 μL of BSA and nuclease-free PCR water. PCR products were cleaned with ExoSAP-IT following the manufacturer’s recommendations and sequenced using an ABI 3730 XL sequencer at LGC Genomics. The specimen barcode can be found under the accession number OK513082 in GenBank.

The identity of the sequence was verified on BLAST (Basic Local Alignment Search Tool), and closely related taxa were added for phylogenetic comparison. The alignment was edited in Geneious 10.2.4, and an appropriate model of evolution was determined in jModelTest v. 2.0.^37^ The Bayesian inference (BI) phylogenetic tree was generated in Mr Bayes v.3.2.6,^38^ and the maximum likelihood (ML) phylogenetic tree was generated online using the server RAxML BlackBox. The BI tree was run for 1 million generations using MCMC and hot and cold chains. Tracer v. 1.5 was used to check for tree convergence, and the first 25% of trees were discarded as burn-in.^39^ The remaining trees were used to estimate posterior probability values (PP), which were indicated at the internode of well-supported clades (>0.85). The ML tree used the GTR model of evolution, and all other parameters were set as default. Support values were estimated from 100 bootstrap pseudoreplicates and indicated at the internode of well-supported clades (>75%). The tree topology was congruent for both methods, and therefore a single tree was presented and rooted with outgroup taxa following the literature. A voucher specimen (MBNUIG380) is stored at the repository of the University of Galway and has been inspected morphologically by Dr. Paco Cardenas.^40^ For the biological description of the specimen, see S1 in the Supporting Information.

### Extraction and Purification

The lyophilized and ground sponge (78.7 g) was extracted with 1:1 CH_3_OH/CH_2_Cl_2_ at room temperature under sonication (3 × 500 mL, 10 min). The resulting solution was concentrated to dryness under reduced pressure. This extract (8.7 g) was sequentially fractionated by C-18 vacuum liquid chromatography (VLC) using mixtures of H_2_O/CH_3_OH and CH_3_OH/CH_2_Cl_2_ of decreasing polarity, into seven fractions: A, H_2_O; B, H_2_O/CH_3_OH (1:1); C, H_2_O/CH_3_OH (1:3); D, CH_3_OH; E, CH_3_OH/CH_2_Cl_2_ (3:1); F, CH_3_OH/CH_2_Cl_2_ (1:1); and G, CH_2_Cl_2_.

Fraction D (0.643 g) was subjected to Sephadex LH-20 column chromatography using a mixture of CH_3_OH/CH_2_Cl_2_ (1:1) to afford three fractions: D1, D2, and D3. Fraction D2 (0.619 g) was subjected to fractionation by reversed-phase HPLC (Waters XSelect Prep C-18, 5 mm; 19 × 250 mm; flow 3.0 mL/min; detection at 210 nm), with a linear gradient of H_2_O/CH_3_CN from 20% to 80% of CH_3_CN over 26 min to allow for the acquisition of several fractions (D2A to D2E) and yielding sarasinoside C_1_ (**1**) (145 mg, *t*_R_ = 15.0 min, 1.84 × 10^–1^% w/w).

Fraction C (0.78 g) was subjected to RP-HPLC (Waters XSelect Prep C-18, 5 mm; 19 × 250 mm; flow 3.0 mL/min; detection at 210 nm), with a linear gradient of H_2_O/CH_3_CN from 35% to 45% of CH_3_CN over 25 min, yielding sarasinoside C_5_ (**3**) (6.0 mg, *t*_R_ = 14.0 min, 7.62 × 10^–3^% w/w) and sarasinoside C_6_ (**4**) (5.7 mg, *t*_R_ = 15.2 min, 7.24 × 10^–3^% w/w).

Fraction D2A (0.177 g) was subjected to fractionation by reversed-phase HPLC (Nucleodur, semipreparative HTec C18, 5 mm; 10 × 250 mm; flow 5.0 mL/min; detection at 230 nm), with a linear gradient of H_2_O/CH_3_CN from 35% to 40% of CH_3_CN over 45 min to provide eight fractions (D2A-1 to D2A-8).

Fraction D2A-4 was purified by reversed-phase HPLC (Waters Xselect Phenyl-hexyl, 5 μm; 4.6 × 250 mm; flow rate: 1 mL/min; UV detection at 230 nm), with a linear gradient of H_2_O/CH_3_CN from 30% to 35% of CH_3_CN over 35 min, yielding sarasinoside C_8_ (**6**) (0.9 mg, *t*_R_ = 27.0 min, 1.14 × 10^–3^% w/w) and sarasinoside C_4_ (**2**) (0.9 mg, *t*_R_ = 29.5 min, 1.14 × 10^–3^% w/w).

Fraction D2A-5 was purified by reversed-phase HPLC (Waters Xselect Phenyl-hexyl, 5 μm; 4.6 × 250 mm; flow rate: 1 mL/min; UV; detection at 230 nm), with a linear gradient of H_2_O/CH_3_CN from 30% to 38% of CH_3_CN over 40 min, yielding sarasinoside C_9_ (**7**) (0.9 mg, *t*_R_ = 29.0 min, 1.14 × 10^–3^% w/w). Fraction D2A-8 was purified under the same chromatographic conditions previously described for fraction D2A-5, yielding sarasinoside C_7_ (**5**) (0.9 mg, *t*_R_ = 20.2 min, 1.14 × 10^–3^% w/w).

Sarasinoside C_1_ (**1**): yellowish amorphous solid; [α]_20_^D^ −26.1 (*c* 0.1, CH_3_CN); UV (MeOH) λ_max_ (log ε) 239 nm (7.02); ECD (*c* 1.45 × 10^–5^ M, MeOH) λ_max_ (Δε) 222 (−3.1) nm; IR (ν̃ _max_) 1041, 1564, 1649, 2870, 2948, 3339 cm^–1^; ^1^H NMR and ^13^C NMR data, CD_3_OD Tables S3 and S4; ^1^H NMR in C_5_D_5_N/D_2_O δ_H_ 6.18 (1H, s, H-24), 5.46 (1H, d, *J* = 8.4), 5.25 (1H, d *J* = 8.3 Hz), 5.01 (1H, overlap), 4.68 (1H, overlap), 3.26 (1H, dd *J* = 11.8, 4.1 Hz, H-3α), 2.23 (3H, s, H_3_-25), 2.11 (3H, s, NHCOCH_3_), 2.09 (3H, s, NHCOCH_3_), 1.77 (3H, s, H_3_-25), 1.35 (3H, s), 1.23 (3H, s), 1.08 (3H, d *J* = 5.8 Hz, H_3_-21), 1.02 (3H, s), 0.67 (3H, s); ESI (+)-HRESIMS *m*/*z* 1097.6000 [M + H]^+^ (calcd for C_55_H_89_N_2_O_20_^+^, 1097.0004, Δ −0.3 ppm).

Sarasinoside C_4_ (**2**): white amorphous solid; [α]_20_^D^ 0 (*c* 0.1, CH_3_CN); UV (H_2_O) λ_max_ (log ε) 245 nm (6.09), ECD (*c* 1.98 × 10^–4^ M, H_2_O) λ_max_ (Δε) 303 (−11.6), 254 (−2.2), 234 (+4.0), 209 (−16.2); ^1^H NMR and ^13^C NMR data, Tables 2 and S4; ESI (+)-HRESIMS *m*/*z* 1111.5800 [M + H]^+^ (calcd for C_55_H_87_N_2_O_21_^+^, 1111.5796, Δ +0.4 ppm).

Sarasinoside C_5_ (**3**): white amorphous solid; [α]_20_^D^ −19.8 (*c* 0.1, CH_3_CN); UV (H_2_O) λ_max_ (log ε) 246 nm (6.35); ECD (*c* 1.11 × 10^–4^ M, H_2_O) λ_max_ (Δε) 246 (−5.4), 219 (−6.3); IR (ν̃_max_) 1036, 1069, 1545, 1641, 2871, 2941, 3311 cm^–1^; ^1^H NMR and ^13^C NMR data, Tables 2 and S4; ESI (+)-HRESIMS *m*/*z* 1143.6039 [M + H]^+^ (calcd for C_56_H_91_N_2_O_22_^+^, 1143.6058, Δ −1.7 ppm).

Sarasinoside C_6_ (**4**): white amorphous solid; [α]_D_^20^ −4.8 (*c* 0.1, CH_3_CN); UV (H_2_O) λ_max_ (log ε) 246 nm (6.35); ECD (*c* 1.11 × 10^–4^ M, H_2_O) λ_max_ (Δε) 246 (−5.4), 219 (−6.3); IR (ν̃_max_) 1036, 1069, 1545, 1641, 2871, 2941, 3311 cm^–1^; ^1^H NMR and ^13^C NMR data, Tables 2 and S4; ESI (+)-HRESIMS *m*/*z* 1143.6058 [M + H]^+^ (calcd for C_56_H_91_N_2_O_22_^+^, 1143.6058, Δ +0.1 ppm).

Sarasinoside C_7_ (**5**): white amorphous solid; [α]_D_^20^ 0 (*c* 0.1, CH_3_CN); UV (H_2_O) λ_max_ (log ε) 245 nm (6.39); ECD (*c* 9.96 × 10^–5^ M, H_2_O) λ_max_ (Δε) 242 (−5.3), 221 (−4.2); ^1^H NMR and ^13^C NMR data, Tables 3 and S5; ESI (+)-HRESIMS *m*/*z* 1133.5648 [M – H_2_O + Na]^+^ (calcd for C_55_H_86_N_2_O_21_Na^+^, 1133.5615, Δ +2.91 ppm).

Sarasinoside C_8_ (**6**): white amorphous solid; [α]_D_^20^ 0 (*c* 0.1, CH_3_CN); UV (H_2_O) λ_max_ (log ε) 245 nm (6.20); ECD (*c* 1.55 × 10^–4^ M, H_2_O) λ_max_ (Δε) 249 (−8.2), 209 (−8.5); ^1^H NMR and ^13^C NMR data, Tables 3 and S5; ESI (+)-HRESIMS *m*/*z* 1129.5866 [M + H]^+^ (calcd for C_55_H_89_N_2_O_22_^+^, 1129.5901, Δ −3.2 ppm).

Sarasinoside C_9_ (**7**): white amorphous solid; [α]_D_^20^ 0 (*c* 0.1, CH_3_CN); UV (H_2_O) λ_max_ (log ε) 244 nm (6.39); ECD (*c* 9.96 × 10^–5^ M, H_2_O) λ_max_ (Δε) 242 (+1.5), 209 (−9.7); ^1^H NMR and ^13^C NMR data, Tables 3 and S5; (+)-HRESIMS *m*/*z* 1133.5599 [M – H_2_O + Na]^+^ (calcd for C_55_H_86_N_2_O_21_Na^+^, 1133.5615, Δ +1.4 ppm).

### Computational Methods

A conformational analysis of the two relative configurations of the aglycone of **6** was performed in Schrodinger MacroModel 2018, constraining the conformations using key ROESY correlations. The conformers were then further optimized in Gaussian 16^41^ with a functional/basis set combination of B3LYP/TZVP (Empirical Dispersion = GD3BJ). At the same time, the energy of each conformer was calculated. The NMR and *J* couplings of each conformer were then calculated in Gaussian 16 with a wB97xd/6-31G* combination.^42^ Finally, the optimized structures and Boltzmann weighted *J* couplings were compared with the experimental NMR data.

## Data Availability

All raw data are deposited in NP-MRD at https://depositions.np-mrd.org/request-data/5ba68331-0137-4165-ac94-8ff1c89b054b.

## Supporting Information Available

Biological description of *Melophlus sarasinorum*; ^1^H and ^13^C NMR tables; NMR and MS spectra of compounds **1**–**7**; molecular modeling data (PDF)
