# Supplementary material for: Additional Sarasinosides from the Marine Sponge Melophlus sarasinorum Collected from the Bismarck Sea
Source: J Nat Prod. 2023 Nov 30;86(12):2730–8. doi: 10.1021/acs.jnatprod.3c01045 (PMC10749473; doi:10.1021/acs.jnatprod.3c01045)
Supplement: Supplementary file 1 — np3c01045_si_001.pdf [file np3c01045_si_001.pdf]

Supplementary information for

Additional sarasinosides from the marine sponge *Melophlus sarasinorum* collected from the Bismarck Sea.

Shauna O'Brien,[‖] *Rodney Lacret,*[‖] *Maggie M. Reddy, Laurence K. Jennings, Pilar Sánchez, Fernando Reyes, Augustine Mungkaje, Kevin Calabro, and Olivier P. Thomas**

Table of Contents

S1: Biological description of *Melophlus sarasinorum* P3

S2: Biological Assays P5

Table S3. ^{1}H and ^{13}C NMR resonances for the aglycone units of **1** P6

Table S4: ^{1}H resonances for the sugar residues of **1-4** in CD_3OD P7

Table S5: ^{1}H resonances for the sugar residues of **5-7** in CD_3OD P8

S6: (+)-HRESIMS analysis of Sarasinoside C_1 (**1**) P9

S7: ^{1}H NMR spectrum of **1** (CD_3OD, 500 MHz) P9

S8: ^{1}H NMR spectrum of **1** (C_5D_5N/D_2O, 500 MHz) P10

S9: ^{13}C NMR spectrum of **1** (CD_3OD, 125 MHz) P10

S10: COSY spectrum of **1** (CD_3OD, 500 MHz) P11

S11: HSQC spectrum of **1** (CD_3OD, 500 MHz) P11

S12: HMBC spectrum of **1** (CD_3OD, 500 MHz) P12

S13: ROESY spectrum of **1** (CD_3OD, 500 MHz) P12

S14: IR spectrum of **1** P13

S15: (+)-HRESIMS analysis of Sarasinoside C_4 (**2**) P14

S16: ^{1}H NMR spectrum of **2** (CD_3OD, 600 MHz) P14

S17: ^{13}C NMR spectrum of **2** (CD_3OD, 150 MHz) P15

S18: COSY spectrum of **2** (CD_3OD, 600 MHz) P15

S19: TOCSY spectrum of **2** (CD_3OD, 600 MHz) P16

S20: HSQC spectrum of **2** (CD_3OD, 600 MHz) P16

S21: HMBC spectrum of **2** (CD_3OD, 600 MHz) P17

S22: ROESY spectrum of **2** (CD_3OD, 600 MHz) P17

S23: (+)-HRESIMS analysis of Sarasinoside C_5 (**3**) P18

S24: ^{1}H NMR spectrum of **3** (CD_3OD, 500 MHz) P18

S25: ^{13}C NMR spectrum of **3** (CD_3OD, 125 MHz) P19

S26: COSY spectrum of **3** (CD_3OD, 500 MHz) P19

S27: HSQC spectrum of **3** (CD_3OD, 500 MHz) P20

S28: TOCSY spectrum of **3** (CD_3OD, 500 MHz) P20

S29: HMBC spectrum of **3** (CD_3OD, 500 MHz) P21

S30: ROESY spectrum of **3** (CD_3OD, 500 MHz) P21

S31: IR spectrum of **3** P22

S32: (+)-HRESIMS analysis of Sarasinoside C_6 (**4**) P23

S33: ^{1}H NMR spectrum of **4** (CD_3OD, 500 MHz) P23

S34: ^{13}C NMR spectrum of **4** (CD_3OD, 125 MHz) P24

S35: COSY spectrum of **4** (CD_3OD, 500 MHz) P24

S36: TOCSY spectrum of **4** (CD_3OD, 500 MHz) P25

S37: HSQC spectrum of **4** (CD_3OD, 500 MHz) P25

S38: HMBC spectrum of **4** (CD_3OD, 500 MHz) P26

S39: ROESY spectrum of **4** (CD_3OD, 500 MHz) P26

S40: IR spectrum of **4** P27

S41: (+)-HRESIMS analysis of Sarasinoside C_8 (**5**) P28

S42: ^{1}H NMR spectrum of **5** (CD_3OD, 600 MHz) P28

S43: ^{13}C NMR spectrum of **5** (CD_3OD, 150 MHz) P29
S44: COSY spectrum of **5** (CD_3OD, 600 MHz) P29
S45: HSQC spectrum of **5** (CD_3OD, 600 MHz) P30
S46: HMBC spectrum of **5** (CD_3OD, 600 MHz) P30
S47: ROESY spectrum of **5** (CD_3OD, 600 MHz) P31
S48: (+)-HRESIMS analysis of Sarasinoside C_9 (**6**) P32
S49: ^{1}H NMR spectrum of **6** (CD_3OD, 600 MHz) P32
S50: ^{13}C NMR spectrum of **6** (CD_3OD, 150 MHz) P33
S51: COSY spectrum of **6** (CD_3OD, 600 MHz) P33
S52: TOCSY spectrum of **6** (CD_3OD, 600 MHz) P34
S53: HSQC spectrum of **6** (CD_3OD, 600 MHz) P34
S54: HMBC spectrum of **6** (CD_3OD, 600 MHz) P35
S55: ROESY spectrum of **6** (CD_3OD, 600 MHz) P35
S56: Expansion of the ROESY spectrum of **6** (CD_3OD, 600 MHz) P36
S57: (+)-HRESIMS analysis of Sarasinoside C_9 (**7**) P37
S58: ^{1}H NMR spectrum of **7** (CD_3OD, 600 MHz) P37
S59: ^{13}C NMR spectrum of **7** (CD_3OD, 150 MHz) P38
S60: COSY spectrum of **7** (CD_3OD, 600 MHz) P38
S61: TOCSY spectrum of **7** (CD_3OD, 600 MHz) P39
S62: HSQC spectrum of **7** (CD_3OD, 600 MHz) P39
S63: HMBC spectrum of **7** (CD_3OD, 600 MHz) P40
S64: ROESY spectrum of **7** (CD_3OD, 600 MHz) P40
S65: Overlaid 3D structures, cartesian coordinates, number of imaginary frequencies, and energy for Sarasinoside R (**8**) *cis*-8*S**,9*S** aglycone conformers P41
S66: Overlaid 3D structures, cartesian coordinates, number of imaginary frequencies, and energy for Sarasinoside R (**8**) *trans*-8*R**,9*S** aglycone conformers P48

S1: Biological description of *Melophlus sarasinorum*.

Our specimen of *M. sarasinorum* was closely related to *Melophlus* sp., a specimen that was also collected from PNG. These taxa formed a well-supported clade (BI:1/PP:98%), sister to species currently assigned to the genus *Caminus* (Figure S1). These taxa combined were resolved in a larger well-supported clade (BI:1/PP:92%) and were firmly placed within the subfamily Erylinae. Our DNA analyses confirms *M. sarasinorum* as a member of the Geodiidae based on DNA. These results

are consistent with those of Morrow and Cardenas (2015).[1] Our specimen, collected from PNG in the southwestern Pacific, is in close proximity to the type locality of the species further supporting our findings.

[1] Morrow, C. C. and P. Cárdenas (2015). "Proposal for a revised classification of the Demospongiae (Porifera)." Frontiers in Zoology **12**(1): 7.

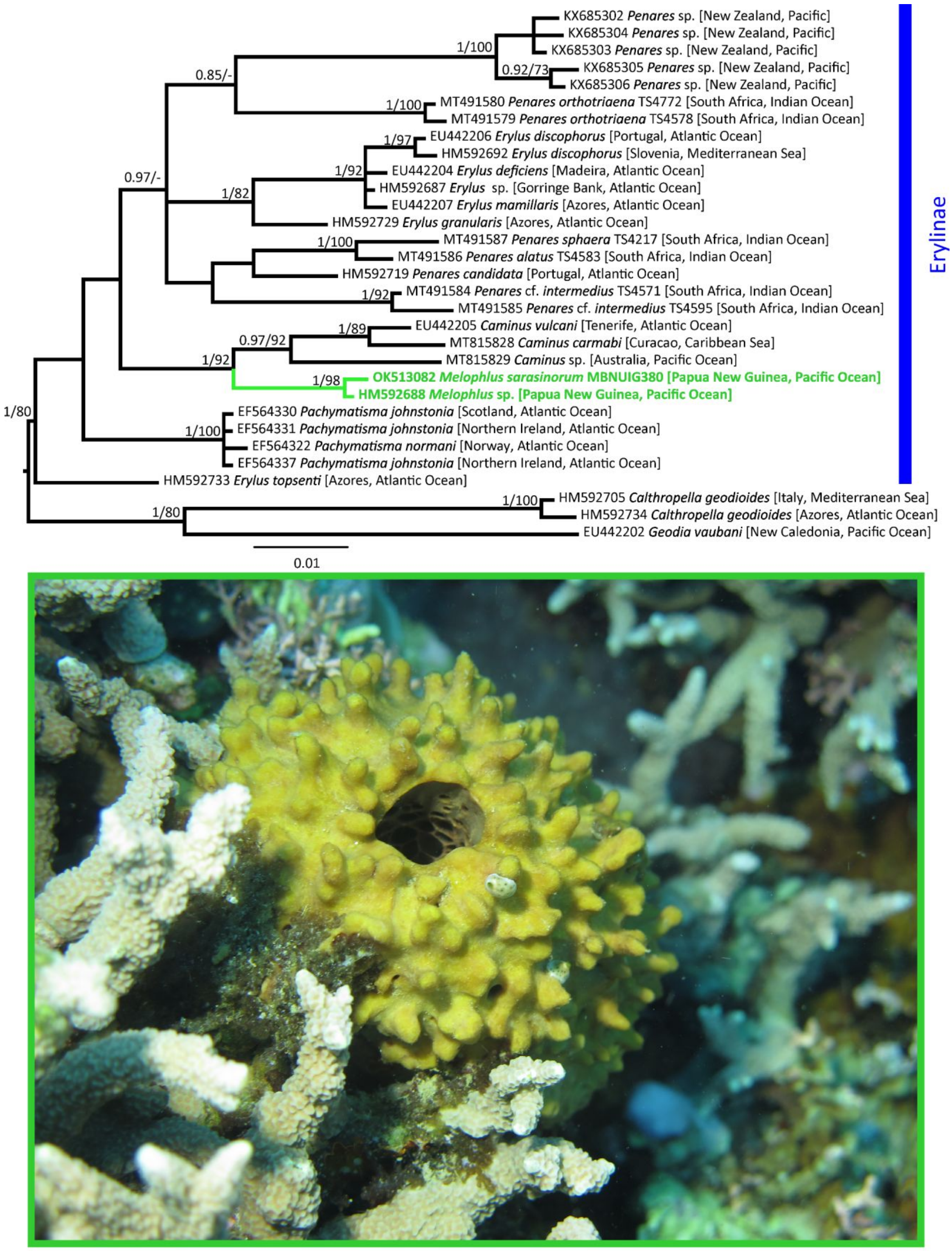

Figure S1. Molecular identification of MBNUIG 380 based on the COI gene and combined BI and ML phylogenetic analyses. PP and BP are presented along well-supported internodes.

S2: Biological Assays

Sarasinosides C_1 (**1**), C_4 (**3**), and C_5 (**4**) were tested in an MTT assay against a panel of cell lines (A549 lung carcinoma, A 2058 metastatic melanoma, HepG2 hepatocyte carcinoma, MCF-7 breast adenocarcinoma and MiaPaCa 2 pancreatic carcinoma).[2] Ten concentrations of each compound ranging between 20-0.039 and µg/mL were tested in triplicate, with **1**, **3** and **4** having no effects on cell viability at the highest concentration tested.

Antimicrobial testing of the compounds was testes using established procedures[3] against Gram-positive bacteria (methicillin sensitive (MSSA) *Staphylococcus aureus*), Gram-negative bacteria (*Escherichia coli*, and *Acinetobacter baumannii*) and fungi (*Candida glabrata*, *Candida albicans*, *Candida krusei*, *Candida parapsilosis*, *Candida tropicalis* and *Aspergillus fumigatus*). Only sarasinoside C_1 exhibited an MIC value of 64 µg/mL against *C. glabrata*, with MIC values > 64 µg/mL against the remaining pathogens. Sarasinosides $C_{3\text{-}4}$ were inactive against all pathogens tested with MIC values higher than 64 µg/mL.

[2] Koagne, R. R.; Annang, F.; Cautain, B.; Martín, J.; Pérez-Moreno, G.;Bitchagno, G. T. M; González-Pacanowska, D.; Vicente, F.; Simo, I. K.; Reyes, F.; Tane, P.. *BMC Complement. Med. Ther.*, **2020**, *20*, 8.

[3] Audoin, C.; Bonhomme, D.; Ivanisevic, J.; de la Cruz, M.; Cautain, B.; Monteiro, M. C.; Reyes, F.; Rios, L.; Perez, T.; Thomas, O. P. Mar. Drugs, **2013**, 11, 1477-1489.

Table S3. ^{1}H and ^{13}C NMR resonances for the aglycone unit of **1** in CD_3OD (^{1}H 500 MHz and ^{13}C 125 MHz).

**No.**	**1**	
	δ_C	δ_H, **Mult. (*J* in Hz)**
**1 α**<br>**1 β**	37.1	1.21, [a]<br>1.72, [a]
**2 α**<br>**2 β**	27.9	1.84, dd (10.0, 3.5)<br>1.72, [a]
**3 α**	91.1	3.09, dd (11.2, 3.9)
**4**	40.6	-
**5 α**	52.0	1.10, [a]
**6 α**<br>**6 β**	19.5	1.70, m<br>1.52, qd (12.3, 6.6)
**7 α**<br>**7 β**	29.5	2.06, [a]<br>1.91, [a]
**8**	128.8	-
**9**	137.3	-
**10**	37.9	-
**11 α**<br>**11 β**	23.0	2.09, br d (4.5) [a]
**12 α**<br>**12 β**	30.0	1.37, [a]<br>1.94, [a]
**13**	43.4	-
**14 α**	53.2	2.06, [a]
**15 α**<br>**15 β**	24.8	1.62, [a]<br>1.34, [a]
**16 α**<br>**16 β**	30.0	1.91, [a]<br>1.34, [a]
**17 α**	56.3	1.21, d (9.9)
**18 β**	11.7	0.68, s
**19 β**	20.1	1.02, s
**20**	35.0	1.99, [a]
**21**	20.1	0.93, d (6.5)
**22**	52.4	2.09, m<br>2.52, dd (14.1, 3.0)
**23**	204.0	
**24**	125.2	6.16, br s
**25**	157.0	
**26**	27.7	1.91, s
**27**	20.9	2.12, s
**28**	17.1	0.91, s
**29**	28.5	1.08, s

[a] overlapping signals

Table S4: NMR data for the glycosides of **1-4** in CD_3OD ([a] ^1H NMR 500 MHz and ^{13}C NMR 125 MHz. [b] ^1H NMR 600 MHz and ^{13}C NMR 150 MHz).

**No.**	**1** [a]		**2** [b]		**3** [b]		**4** [b]	
	δ_C	δ_H, Mult. (*J* in Hz)	δ_C	δ_H, Mult. (*J* in Hz)	δ_C	δ_H, Mult. (*J* in Hz)	δ_C	δ_H, Mult. (*J* in Hz)
***β*-D-Xylose**								
**1'**	105.7	4.35, d (7.5)	105.7	4.34, d (7.5)	105.9	4.36, d (7.3)	105.9	4.36, d (7.4)
**2'**	79.4	3.68, [c]	79.4	3.69, (9.0, 7.5)	79.4	3.68, dd (9.1, 7.3)	79.4	3.68, dd (8.7, 7.3)
**3'**	77.1	3.54, [c]	77.1	3.53, t (9.0)	77.2	3.54, t (9.0)	77.2	3.54, t (8.7)
**4'**	79.4	3.65, [c]	79.4	3.64, mc	79.4	3.65, m [d]	79.4	3.65, m
**5'**	64.0	3.18, [c]<br>3.87, [c]	64.0	3.18, dd (11.5, 10.0)<br>3.86, dd (11.5, 5.5)	64.0	3.18, dd (11.5, 10.0)<br>3.87, dd (11.0, 5.5)	64.0	3.18, dd (11.5, 10.0)<br>3.87, dd(11.5,5.5)
**β-D-2NAc-glucosamine**								
**1''**	102.1	4.88, d (7.5) c	102.1	4.89 (8.0)	102.1	4.88, d (8.2) c	102.1	4.88, d (8.3) c
**2''**	58.0	3.66, [c]	58.0	3.66, dd (10.5, 8.0)	58.0	3.66, dd (10.4, 8.2)	58.0	3.68, t (8.5)
**3''**	76.7	3.43, [c]	76.7	3.41, dd (10.5, 8.5)	76.7	3.42, dd (10.3, 9.0)	76.7	3.42, dd (10.1, 8.8)
**4''**	72.5	3.25, t (9.1)	72.5	3.26, dd (8.5, 10.0)	72.5	3.26, t (9.0)	72.5	3.26, dd (10.0, 9.1)
**5''**	77.1	3.42, [c]	77.2	3.41, [c]	77.1	3.42, ddd (9.1, 6.3, 2.1)	77.1	3.42, [c]
**6''**	69.9	3.68, dd (11.5, 6.5) d<br>4.10, dd (11.5, 1.8)	69.9	3.69, dd (11.6, 6.5)<br>4.09, dd (11.5 2.0)	70.0	3.69, dd (11.4, 6.5)<br>4.10, dd (11.4, 2.0)	70.0	3.68, dd (11.6, 6.5)<br>4.09, br dd (11.6, 2.0)
**N-CO**	174.0		174.0		174.0		174.0	
**CO-CH$_3$**	23.0	1.99, s	23.0	1.99, s	23.0	1.99, s	23.0	2.00, s
**β-D-Xylose**								
**1'''**	105.2	4.42, d (7.5)	105.2	4.44, m	105.2	4.42, d (7.4)	105.2	4.42, d (7.4)
**2'''**	75.1	3.20, dd (9.0, 7.4)	75.1	3.18, dd (8.9, 7.5)	75.1	3.19, dd (9.0, 7.4)	75.1	3.19, dd (7.4, 8.9)
**3'''**	77.6	3.36, t (9.0)	77.6	3.36, t (8.9)	77.6	3.36, t (8.9)	77.6	3.36, t (8.9)
**4'''**	71.2	3.48, ddd (10.1, 8.7, 5.3)	71.3	3.48, ddd (10.1, 8.8, 5.2)	71.3	3.48, ddd (10.0, 8.8, 5.4)	71.3	3.48, ddd (9.9, 8.9, 5.3)
**5'''**	66.9	3.24, dd (11.6, 10.1) c<br>3.82, d (11.6) c	66.9	3.24, dd (11.6, 10.2)<br>3.83, dd (11.5, 5.4)	66.9	3.23, dd (11.7, 10.1)<br>3.83, dd (11.7, 5.4)	66.9	3.24, dd (11.4, 10.2)<br>3.83, dd (11.4, 5.4)
***β*-D-2*N*Ac-galactosamine**								
**1''''**	102.4	4.47, d (8.0)	102.4	4.47, d (8.3)	102.4	4.47, d (8.4)	102.4	4.47, d (8.4)
**2''''**	54.4	3.89, dd (10.7, 8.3)	54.4	3.89, dd (10.7, 8.3)	54.4	3.90, dd (10.6, 8.4)	54.4	3.89, dd (10.5, 8.4)
**3''''**	72.8	3.62, [c]	72.9	3.62, c	72.9	3.62, dd (10.6, 3.2)	72.9	3.62, dd (10.5, 3.2)
**4''''**	69.6	3.82, [c]	69.7	3.82, c	69.6	3.82, d (3.2)	69.6	3.82, d (3.3)
**5''''**	77.2	3.55, [c]	77.3	3.55, c	77.3	3.55, m	77.3	3.55, [d]
**6''''**	62.7	3.73, dd (11.6, 4.5)<br>3.81, dd (11.5, 7.5)	62.7	3.72, dd (11.6, 4.5)<br>3.80, dd (11.6, 7.5)	62.7	3.73, dd (11.6, 4.6)<br>3.81, dd (11.6, 7.5)	62.7	3.73, dd (11.5, 4.4)<br>3.81, dd (11.0, 7.6)
**N-CO**	174.1		174.1		174.1		174.1	
**CO-CH$_3$**	23.0	1.99, s	23.0	1.98, s	23.0	1.99, s	23.0	1.99, s

[c] **overlapping signals**

Table S5: NMR data for the glycosides of **5-7** in CD_3OD (^1H 600 MHz and ^{13}C 150 MHz).

No.	5		6		7	
	δ_C	δ_H, Mult. (*J* in Hz)	δ_C	δ_H, Mult. (*J* in Hz)	δ_C	δ_H, Mult. (*J* in Hz)
***β*-D-Xylose**						
**1’**	105.6	4.33, d (7.4)	105.8	4.34, d (7.3)	105.8	4.35, d (7.4)
**2’**	79.7	3.65, *a*	79.3	3.66, *a*	79.3	3.66, *a*
**3’**	77.1	3.55, *a*	77.2	3.56, *a*	77.3	3.54, d (8.9)
**4’**	79.4	3.64, *a*	79.4	3.66, *a*	79.4	3.66, *a*
**5’**	64.0	3.16, dd (11.3, 10..0)<br>3.86, dd (11.2, 5.2)	64.0	3.17, *a*<br>3.84, t (5.2)	66.9	3.19, *a*<br>3.82, *a*
**β-D-2NAc-glucosamine**						
**1’’**	102.3	4.86, a	102.1	4.88, a	102.3	4.86, a
**2’’**	58.0	3.64, a	58.0	3.63, a	58.0	3.64, a
**3’’**	77.2	3.41, a	76.7	3.43, a	77.2	3.41, a
**4’’**	72.4	3.29, d (9.5)	72.5	3.25, d (9.1)	72.6	3.26, t (9.7)
**5’’**	77.2	3.42, a	76.7	3.43, a	77.2	3.41, a
**6’’**	69.9	3.68, dd (11.6, 6.3)<br>4.11, br dd (11.5, 1.8)	69.9	3.68, dd (11.5, 6.5), a<br>4.08, dd (11.5, 1.8)	69.9	3.67, a<br>4.10, dd (11.2, 1.8)
**N-CO**	174.0		174.0		174.0	
**CO-CH3**	23.0	2.00, s	23.0	1.98, s	23.0	3.64, a
**β-D-Xylose**						
**1’’’**	105.3	4.42, t (9.6)	105.3	4.42, d (7.4)	105.2	4.42, d (7.4)
**2’’’**	75.1	3.19, dd (8.9, 7.4)	75.1	3.19, a	75.1	3.19, a
**3’’’**	77.6	3.36, t (8.9)	77.6	3.35, a	77.6	3.36, t (8.9)
**4’’’**	71.3	3.49, ddd (10.0, 8.8, 5.1) a	71.3	3.48, ddd (10.1, 8.7, 5.3)	71.3	3.48, ddd (10.1, 8.8, 5.3)
**5’’’**	66.9	3.24, d (10.3)<br>3.84, a	66.9	3.24, d (11.6)<br>3.82, d (11.6) a	66.9	3.24, d (11.7)<br>3.85, a
***β*-D-2*N*Ac-galactosamine**						
**1’’’’**	102.3	4.47, d (8.3)	102.3	4.47, d (8.3)	102.1	4.47, d (8.4)
**2’’’’**	54.4	3.88, dd (10.7, 8.3)	54.4	3.89, dd (10.7, 8.3)	54.4	3.89, dd (10.7, 8.4)
**3’’’’**	72.8	3.61 dd (10.7, 3.2)	72.8	3.62, *a*	72.9	3.62, *a*
**4’’’’**	69.7	3.82, *a*	69.7	3.82, *a*	69.7	3.82, *a*
**5’’’’**	77.2	3.55, *a*	77.2	3.55, *a*	77.3	3.54, dd (7.6, 4.5)
**6’’’’**	62.7	3.73, dd (11.6, 4.5)<br>3.80, dd (11.6, 7.5)	62.7	3.72, dd (11.6, 4.5)<br>3.80, dd (11.5, 7.5)	62.7	3.72, dd (11.5, 4.4)<br>3.81, dd (11.5, 7.5)
**N-CO**	174.1		174.1		174.1	
**$CO-CH_3$**	23.0	1.98, s	23.0	1.98, s	23.0	1.99, s

[a] overlapping signals

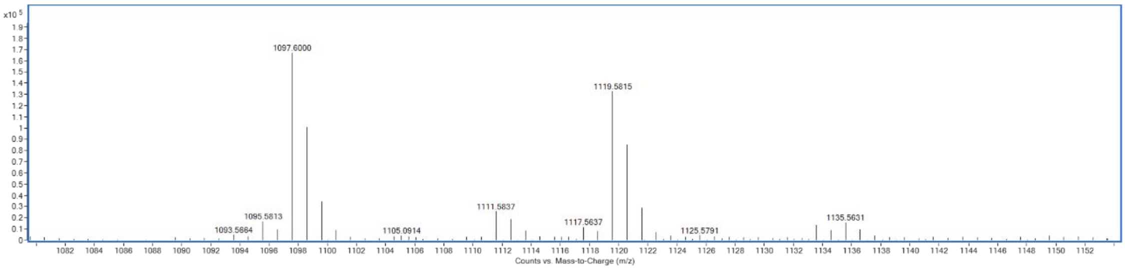

S6: (+)-HRESIMS analysis of Sarasinoside C_1 (**1**).

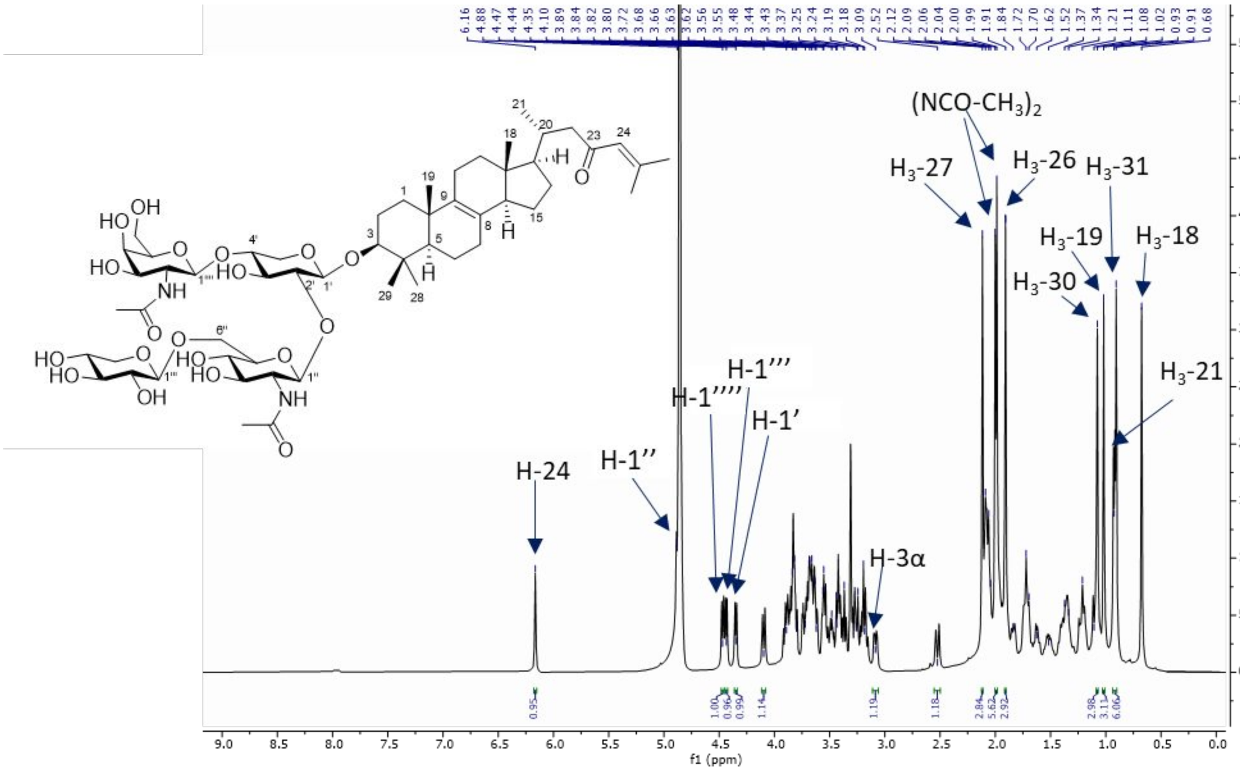

S7: ^1H NMR spectrum of **1** (CD_3OD, 500 MHz).

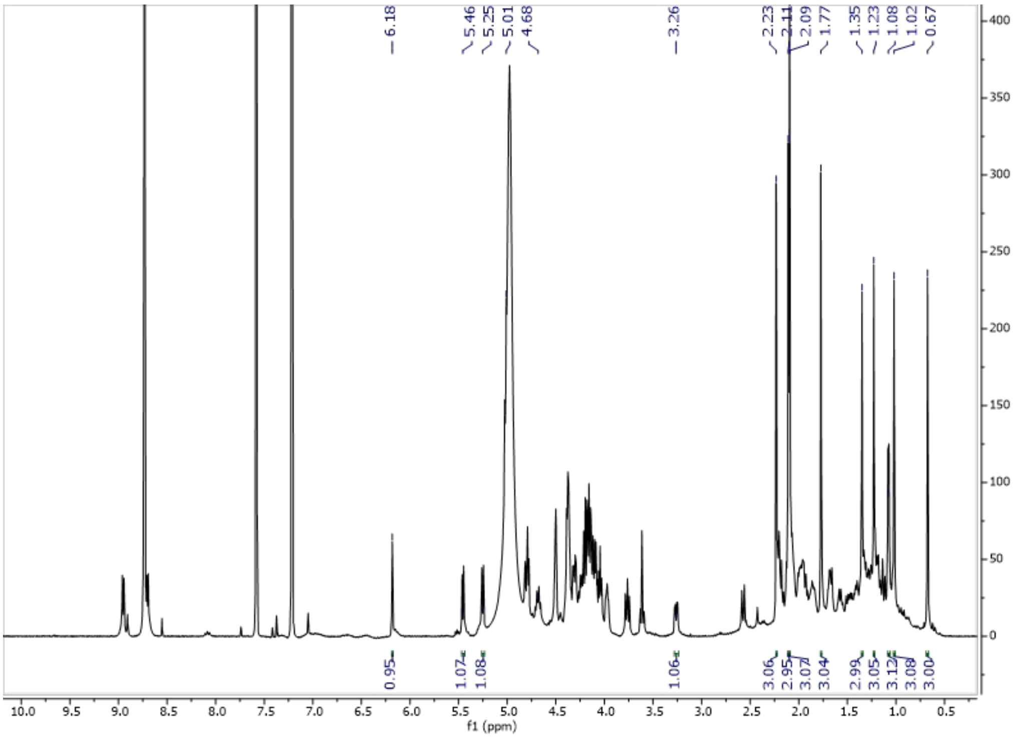

S8: ^{1}H NMR spectrum of **1** (C_5D_5N/D_2O, 500 MHz).

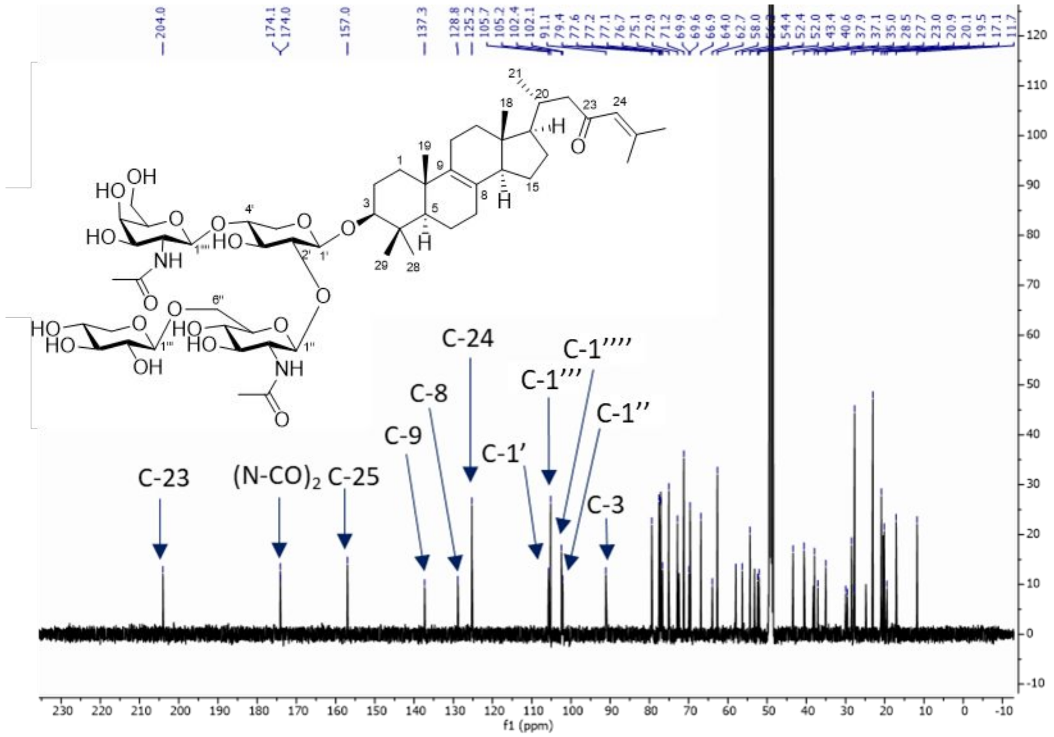

S9: ^{13}C NMR spectrum of **1** (CD_3OD, 125 MHz).

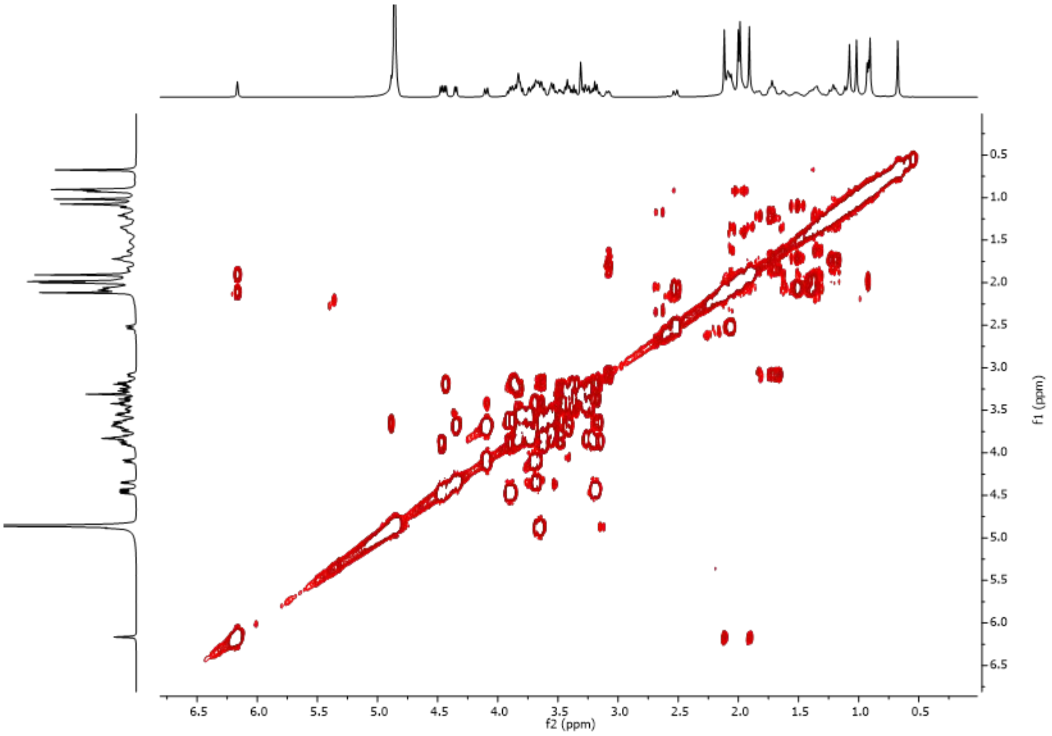

S10: COSY spectrum of **1** (CD_3OD, 500 MHz).

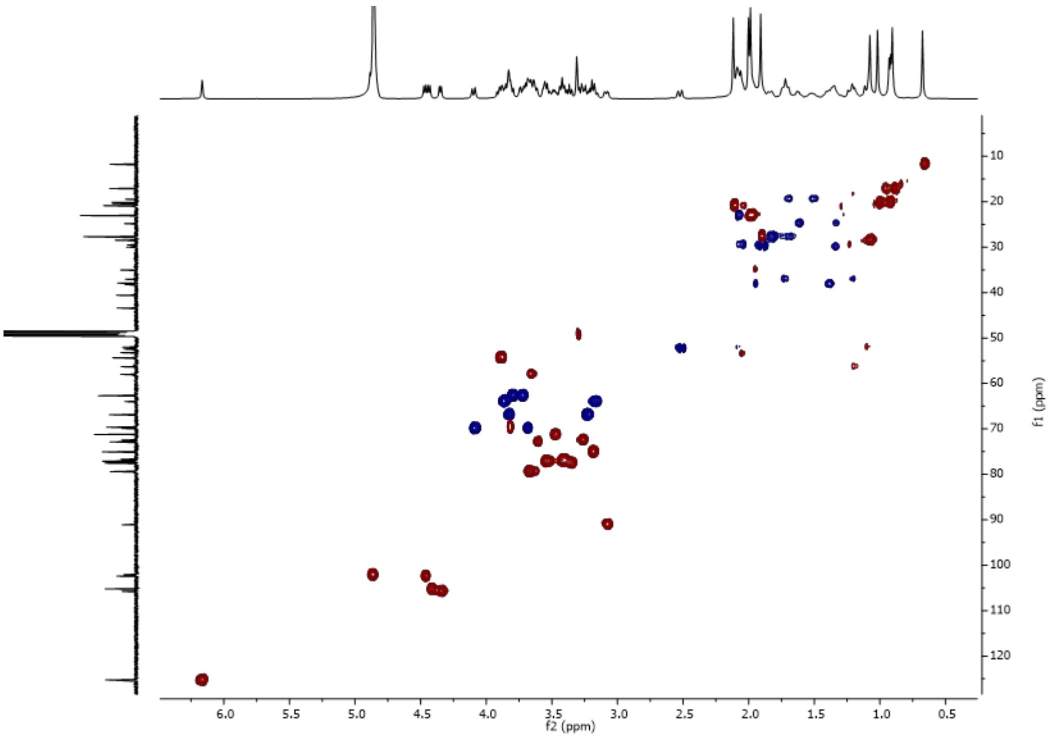

S11: HSQC spectrum of **1** (CD_3OD, 500 MHz).

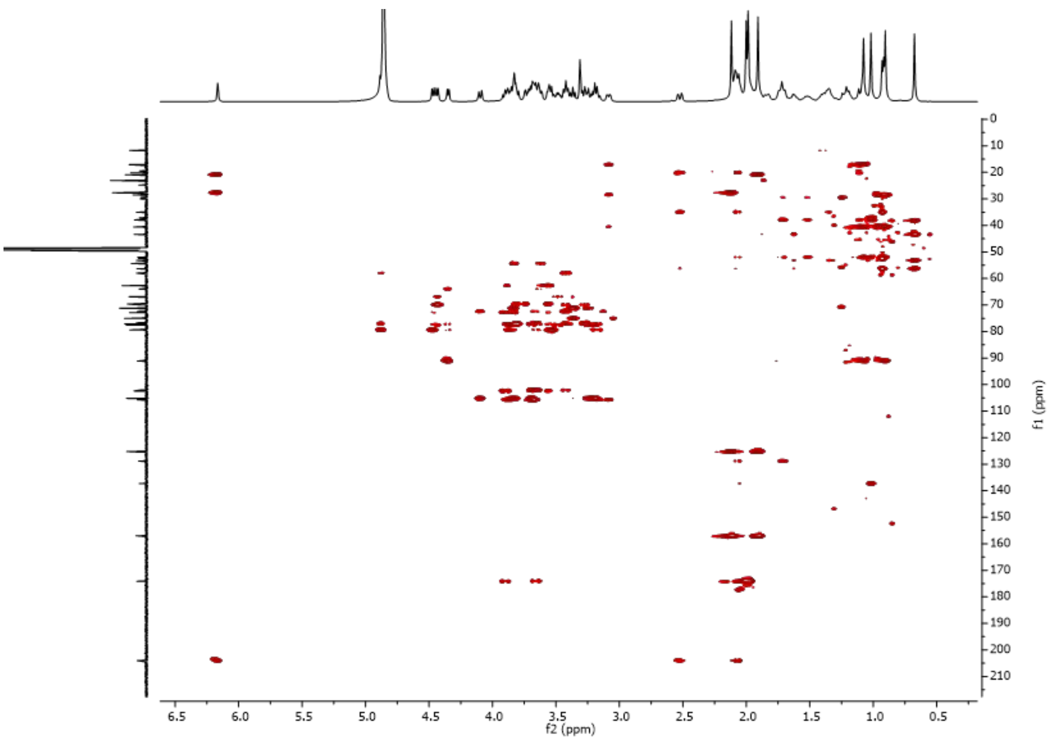

S12: HMBC spectrum of **1** (CD_3OD, 500 MHz).

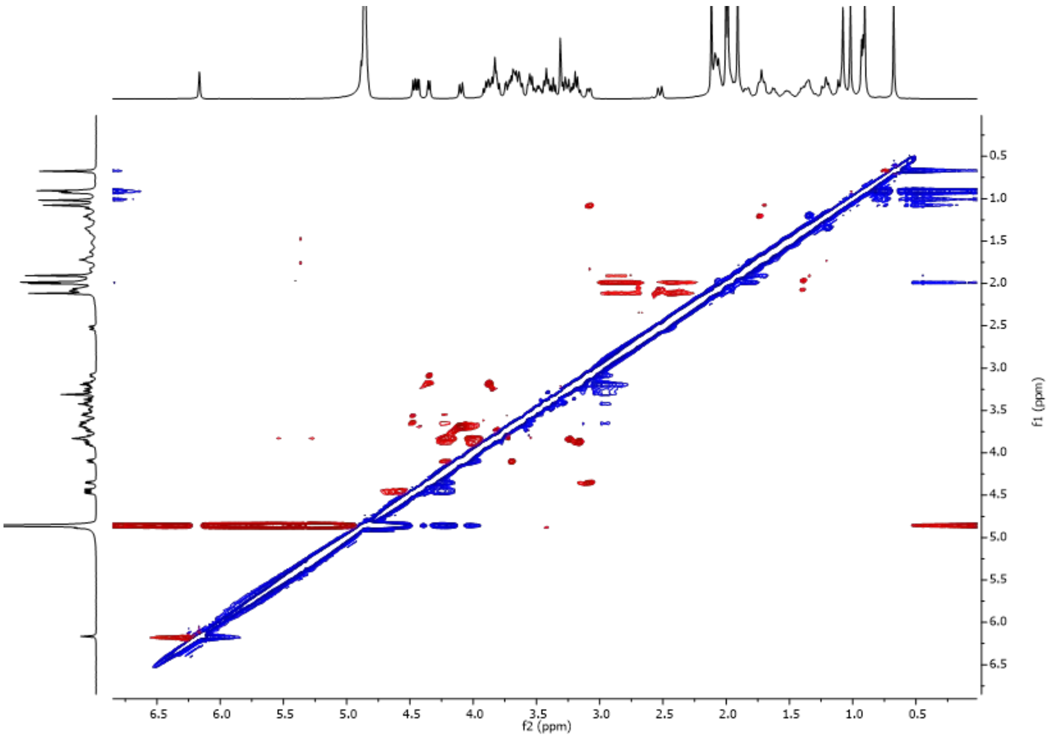

S13: ROESY spectrum of **1** (CD_3OD, 500 MHz).

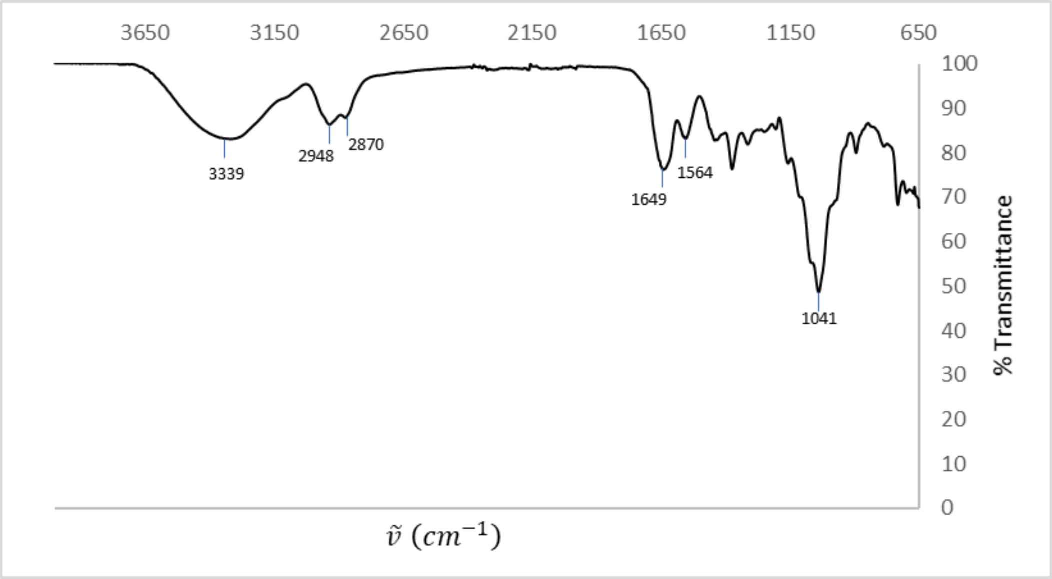

S14: IR spectrum of **1** (Film in a ATR instrument).

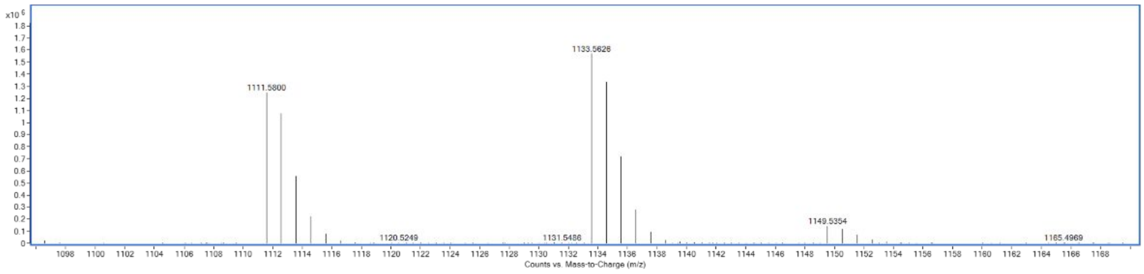

S15: (+)-HRESIMS analysis of Sarasinoside C_4 (**2**).

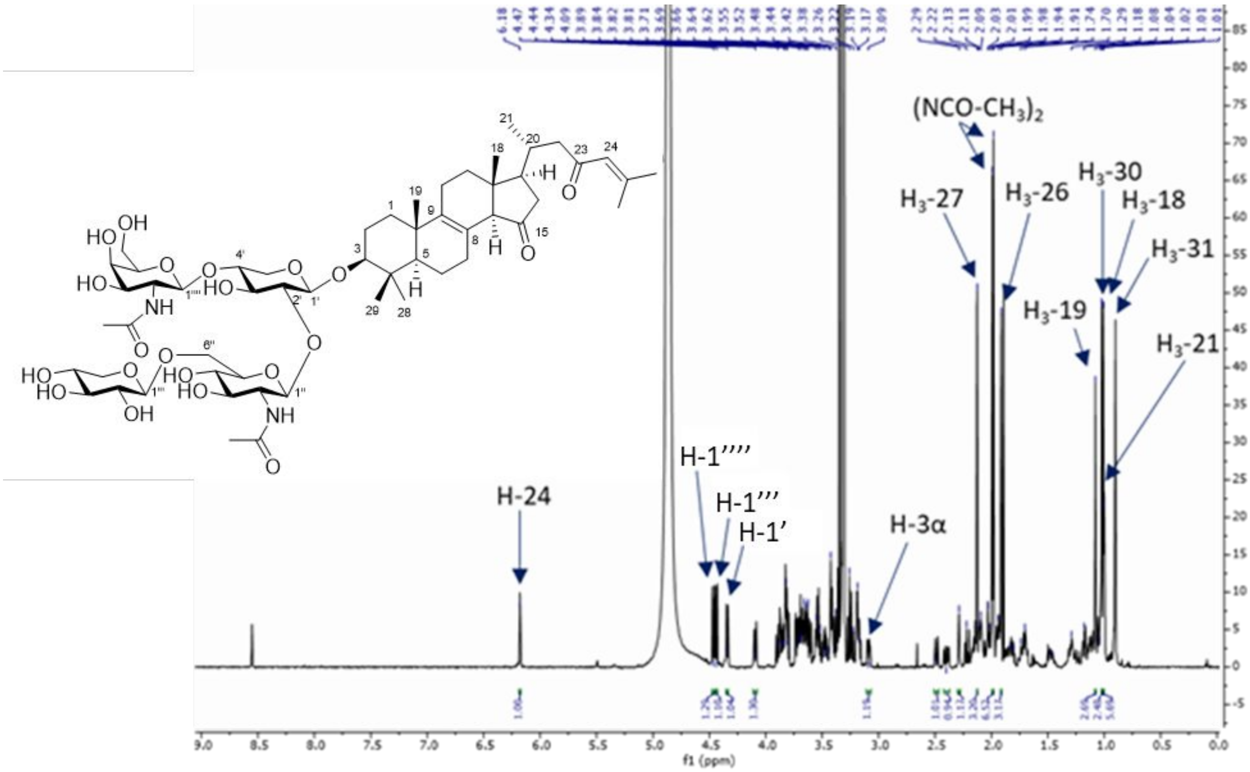

S16: ^{1}H NMR spectrum of **2** (CD_3OD, 600 MHz).

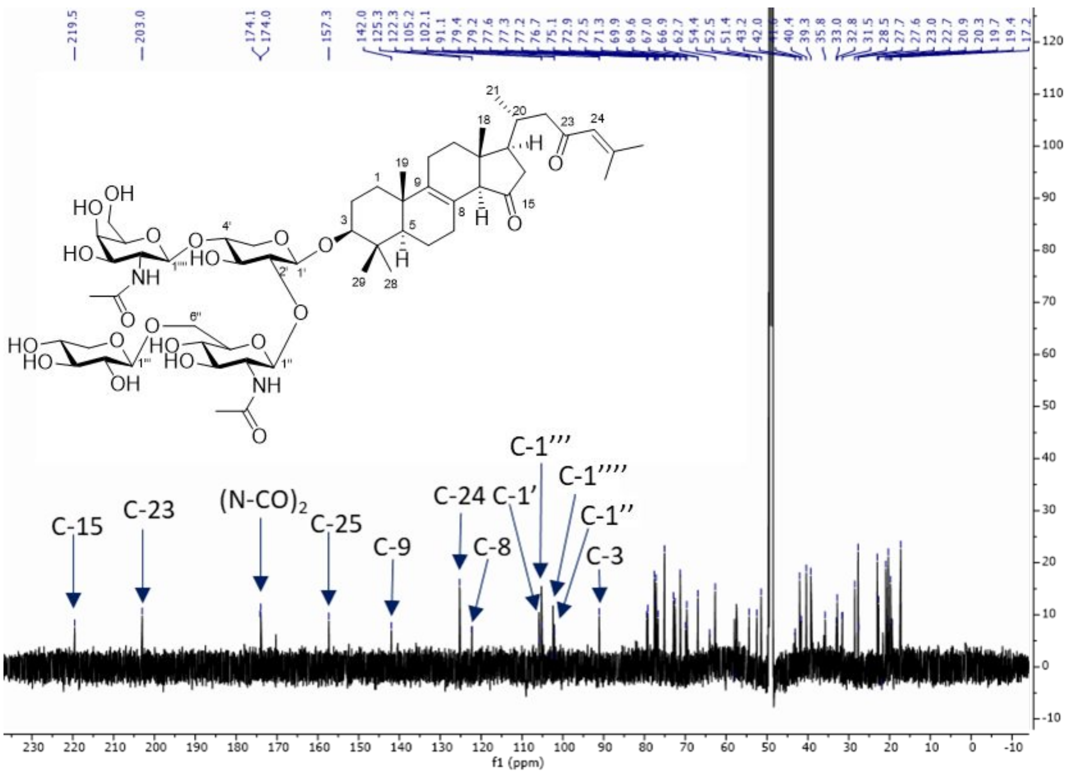

S17: ^{13}C NMR spectrum of **2** (CD_3OD, 150 MHz).

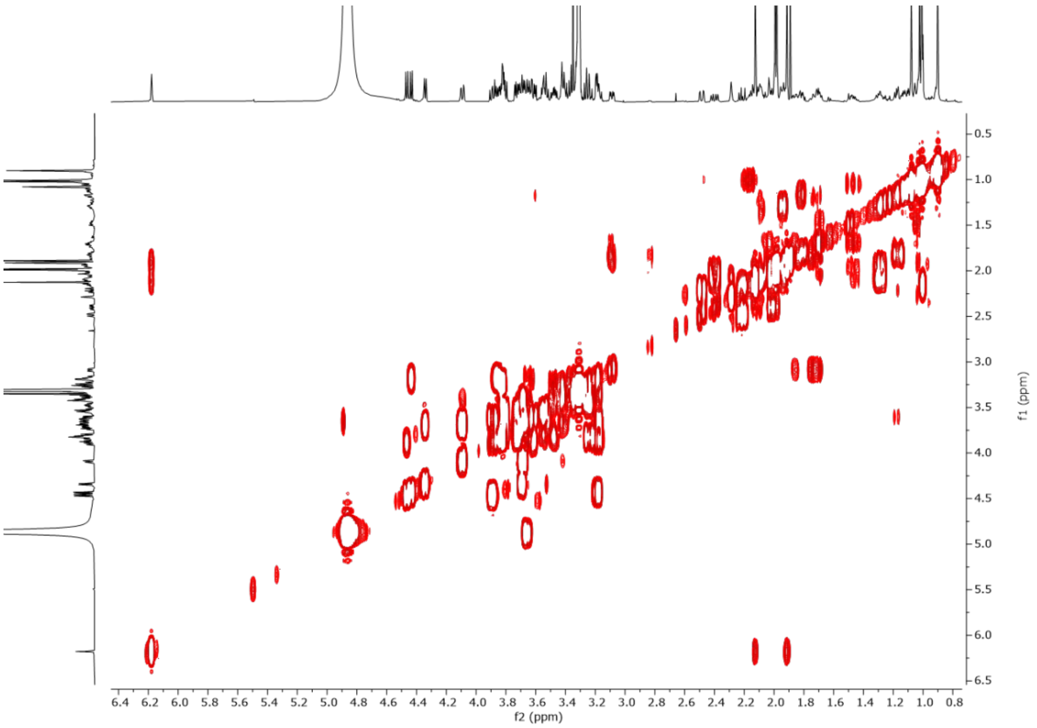

S18: COSY spectrum of **2** (CD_3OD, 600 MHz).

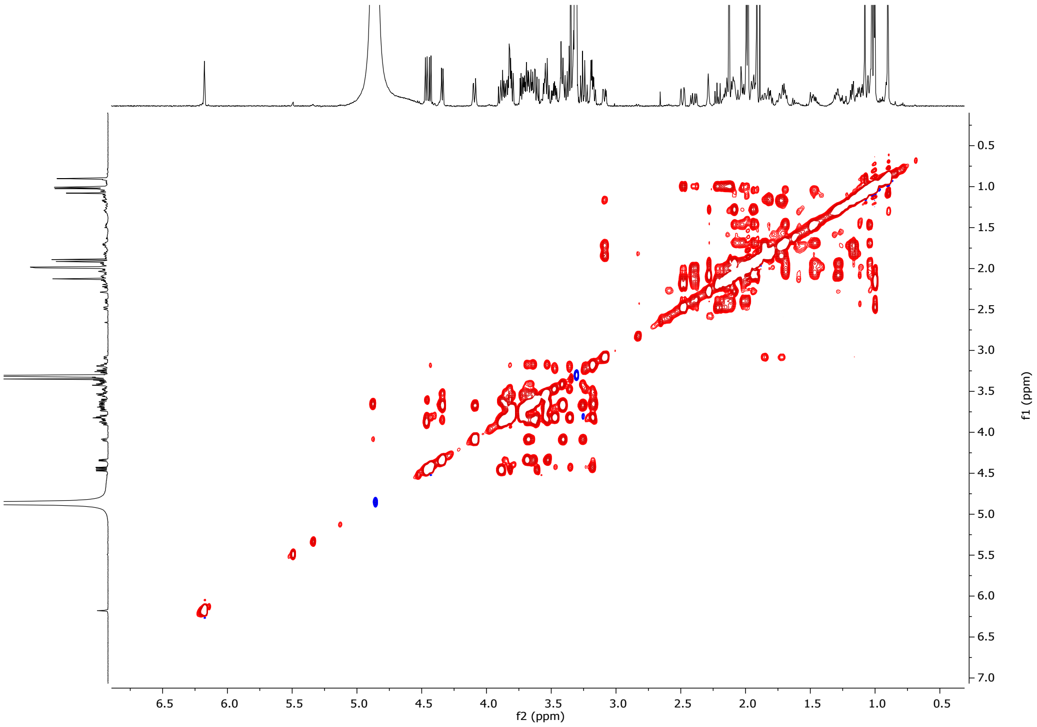

S19: TOCSY spectrum of **2** (CD_3OD, 600 MHz).

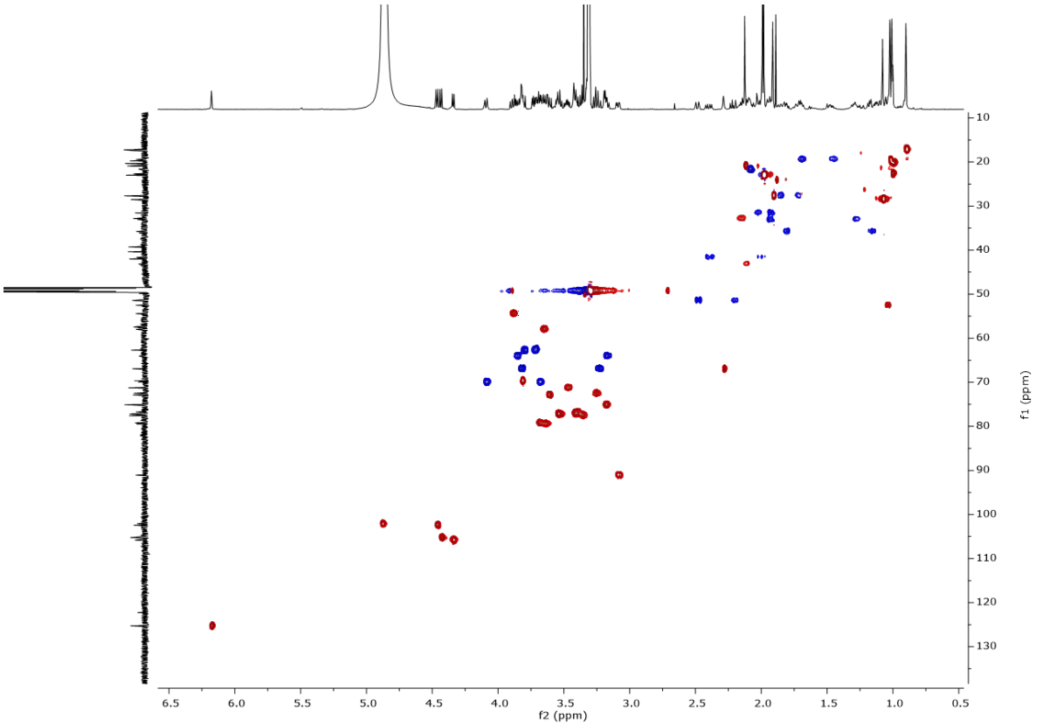

S20: HSQC spectrum of **2** (CD_3OD, 600 MHz).

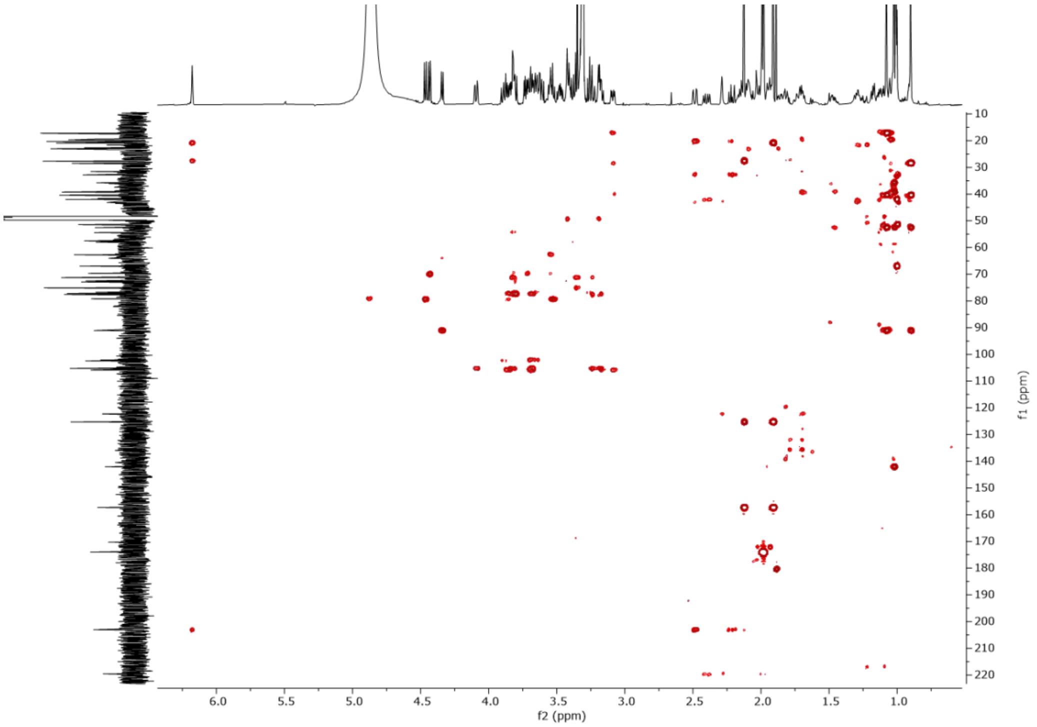

S21: HMBC spectrum of **2** (CD_3OD, 600 MHz).

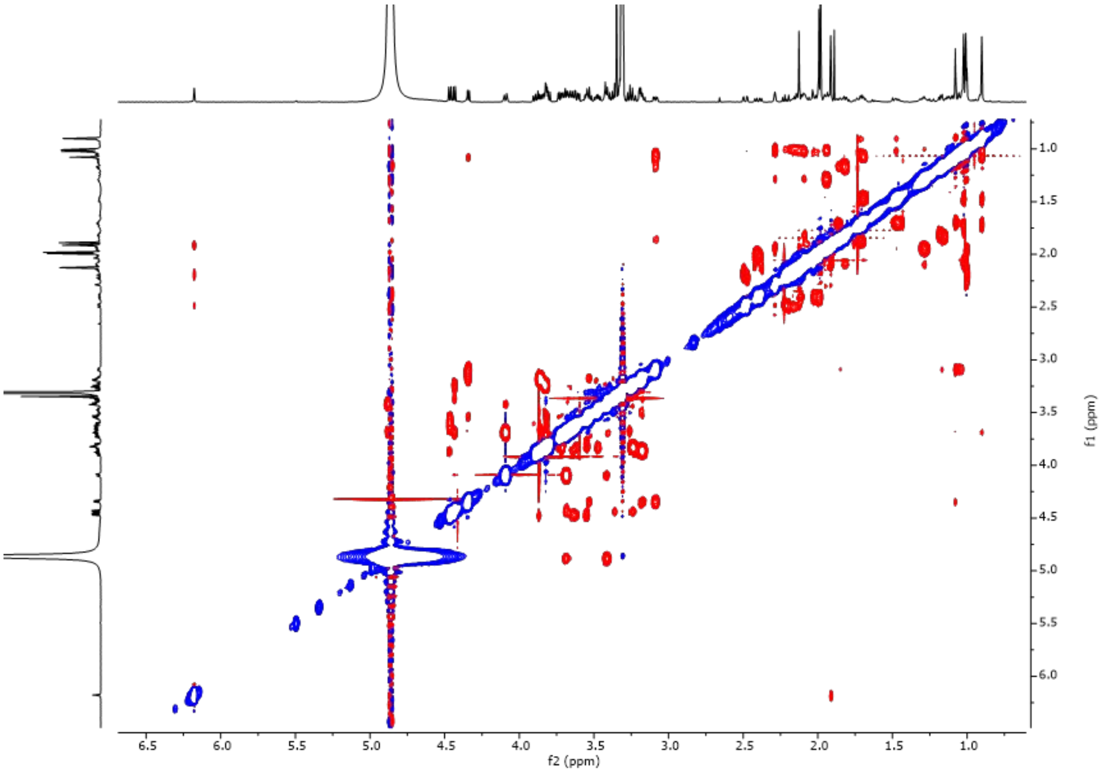

S22: ROESY spectrum of **2** (CD_3OD, 600 MHz).

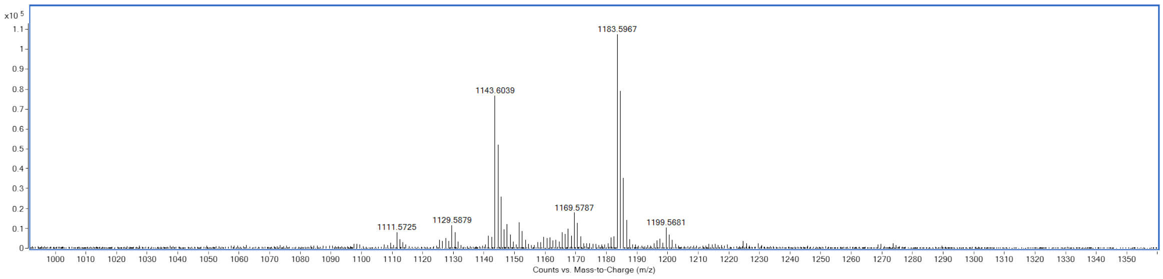

S23: (+)-HRESIMS analysis of Sarasinoside C_5 (**3**).

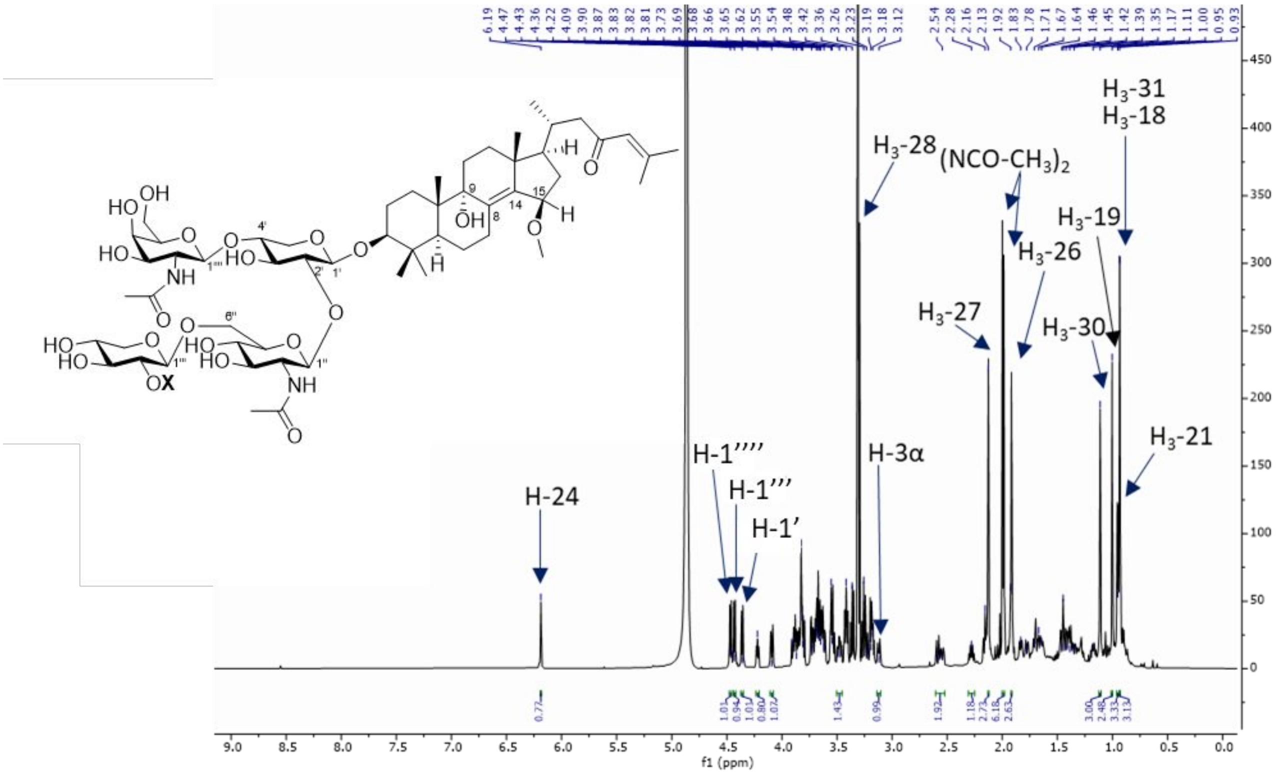

S24: ^{1}H NMR spectrum of **3** (CD_3OD, 500 MHz).

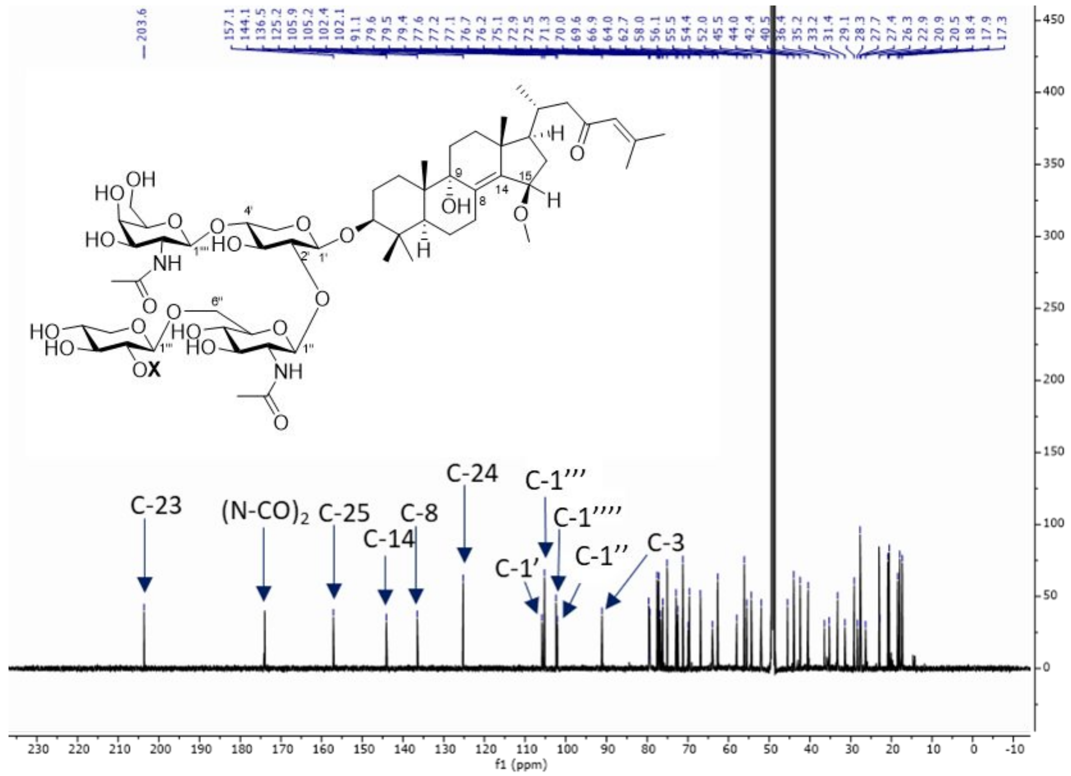

S25: ^{13}C NMR spectrum of **3** (CD_3OD, 125 MHz).

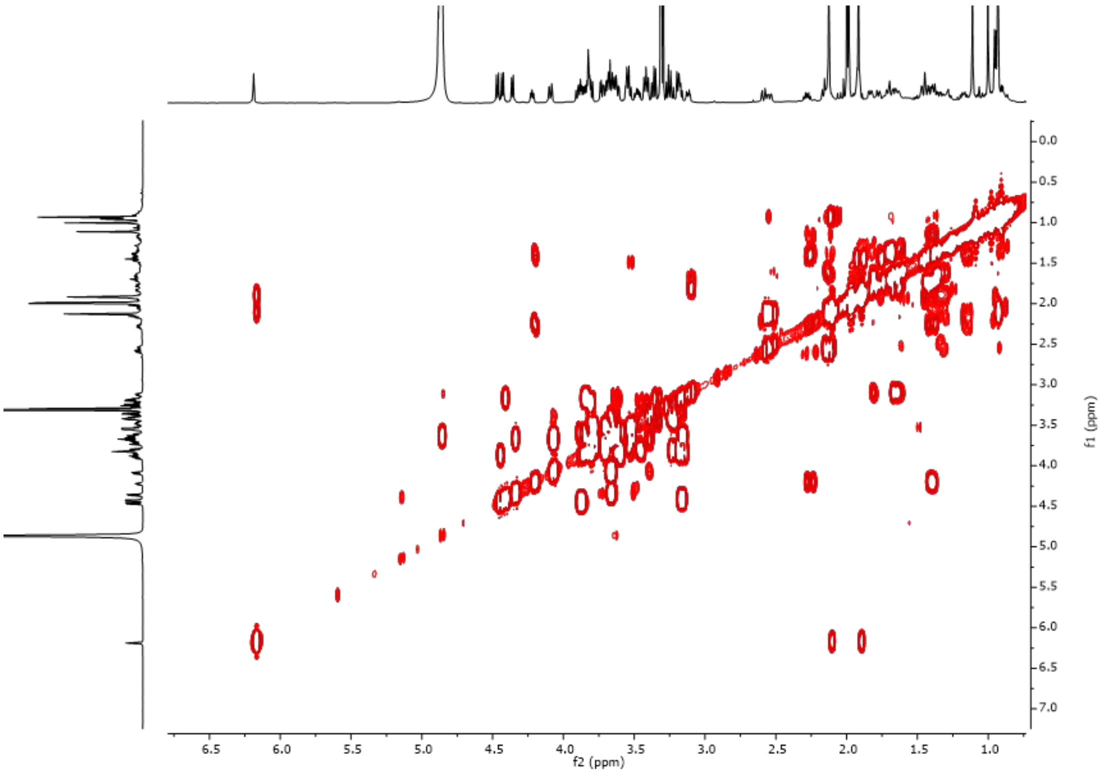

S26: COSY spectrum of **3** (CD_3OD, 500 MHz).

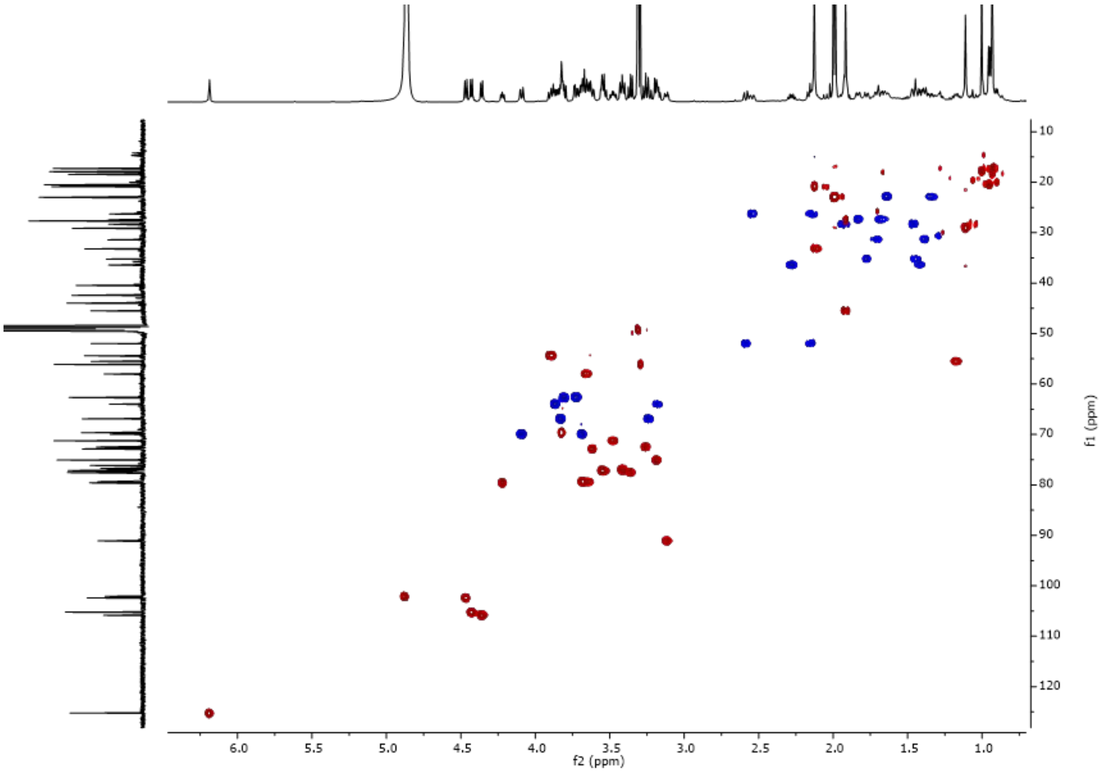

S27: HSQC spectrum of **3** (CD_3OD, 500 MHz).

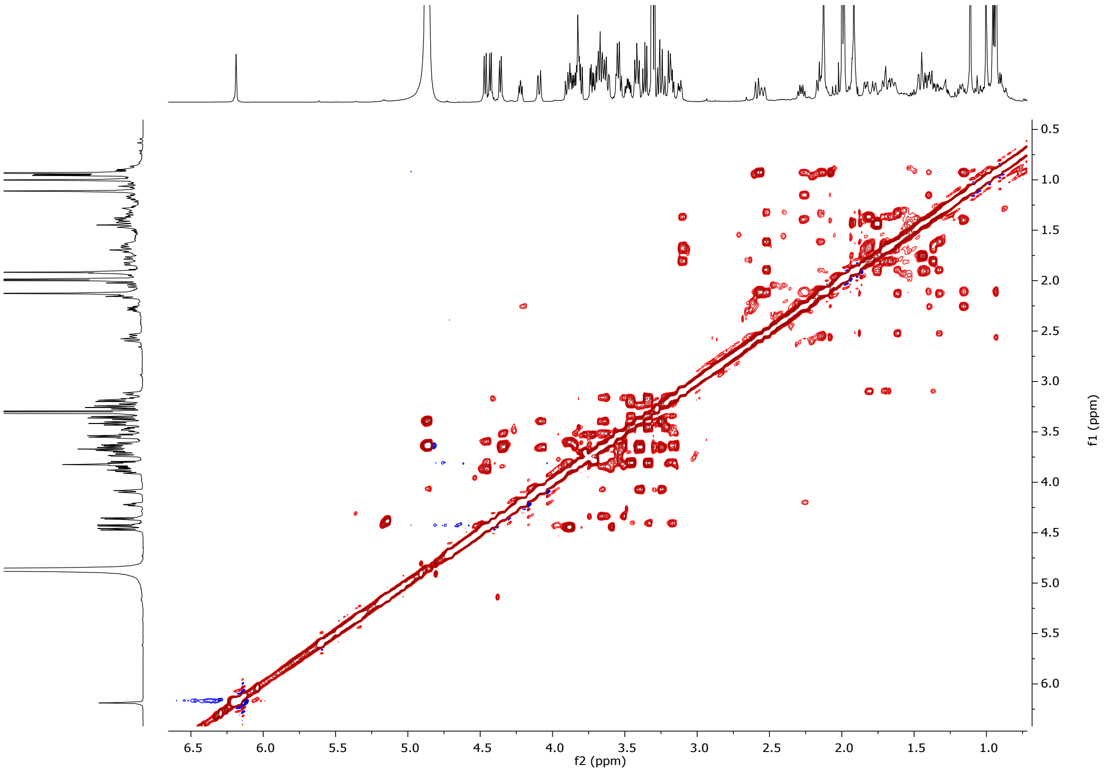

S28: TOCSY spectrum of **3** (CD_3OD, 500 MHz).

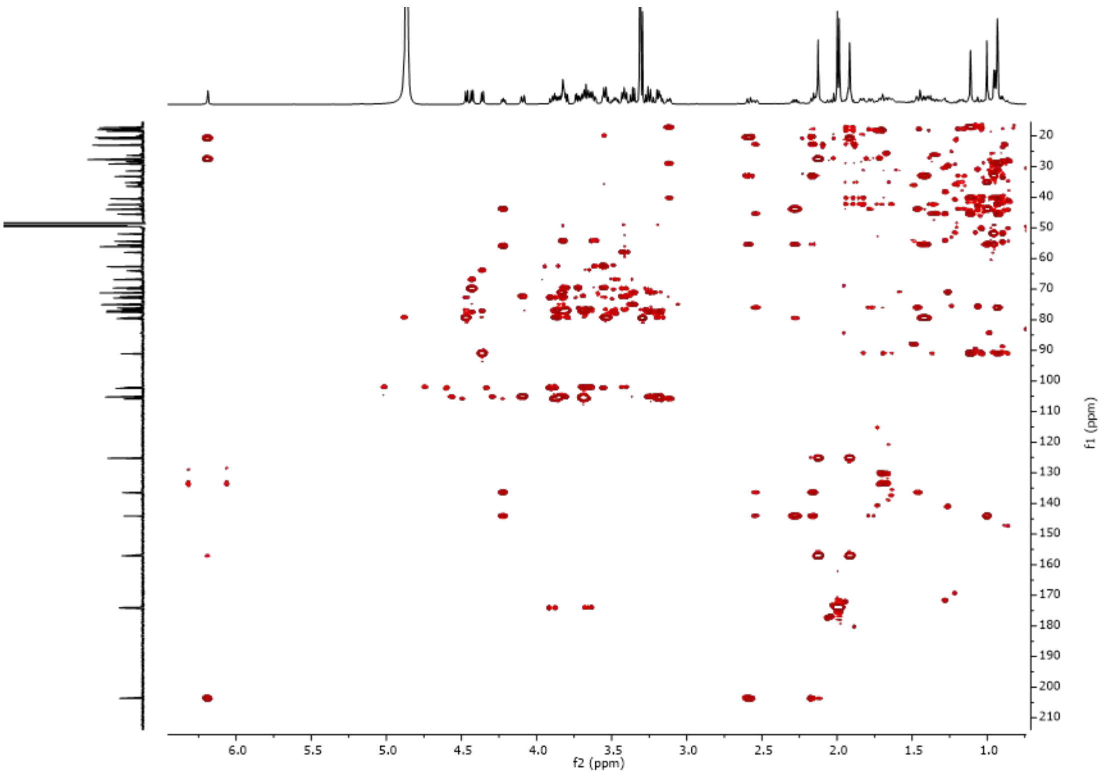

S29: HMBC spectrum of **3** (CD_3OD, 500 MHz).

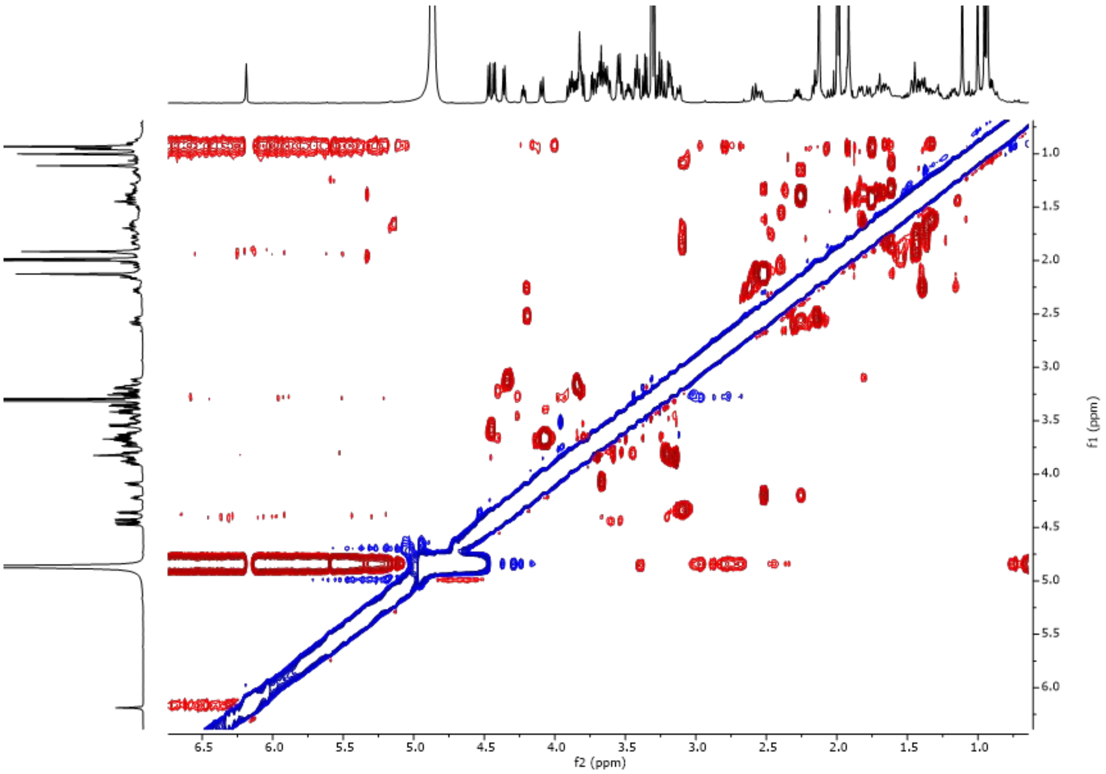

S30: ROESY spectrum of **3** (CD_3OD, 500 MHz).

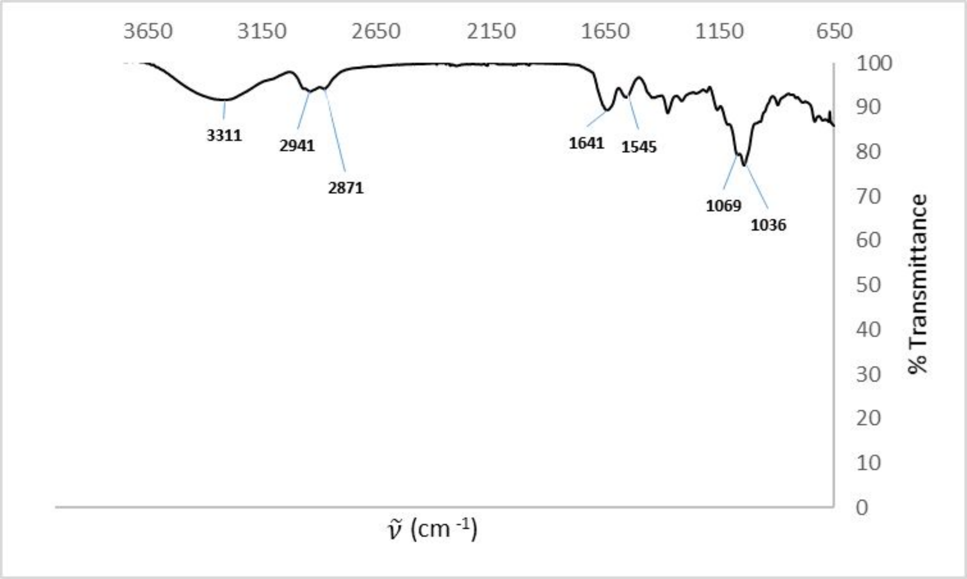

S31: IR spectrum of **3** (Film in a ATR instrument).

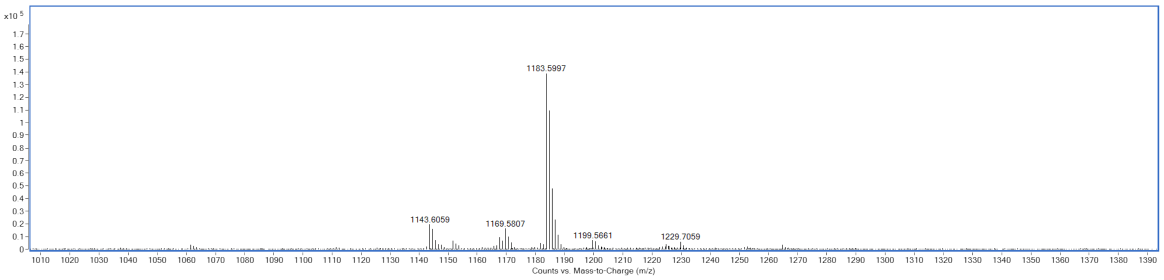

S32: (+)-HRESIMS analysis of sarasinoside C_6 (**4**).

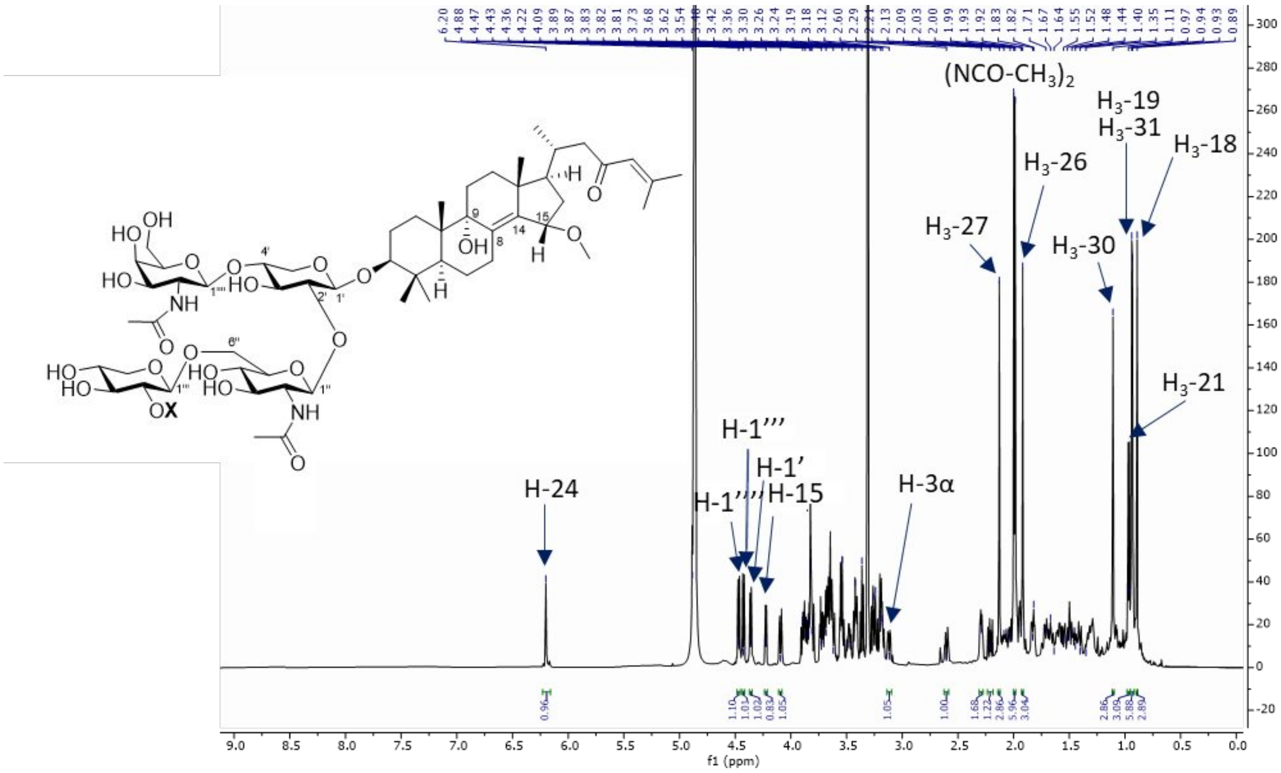

S33: ^{1}H NMR spectrum of **4** (CD_3OD, 500 MHz).

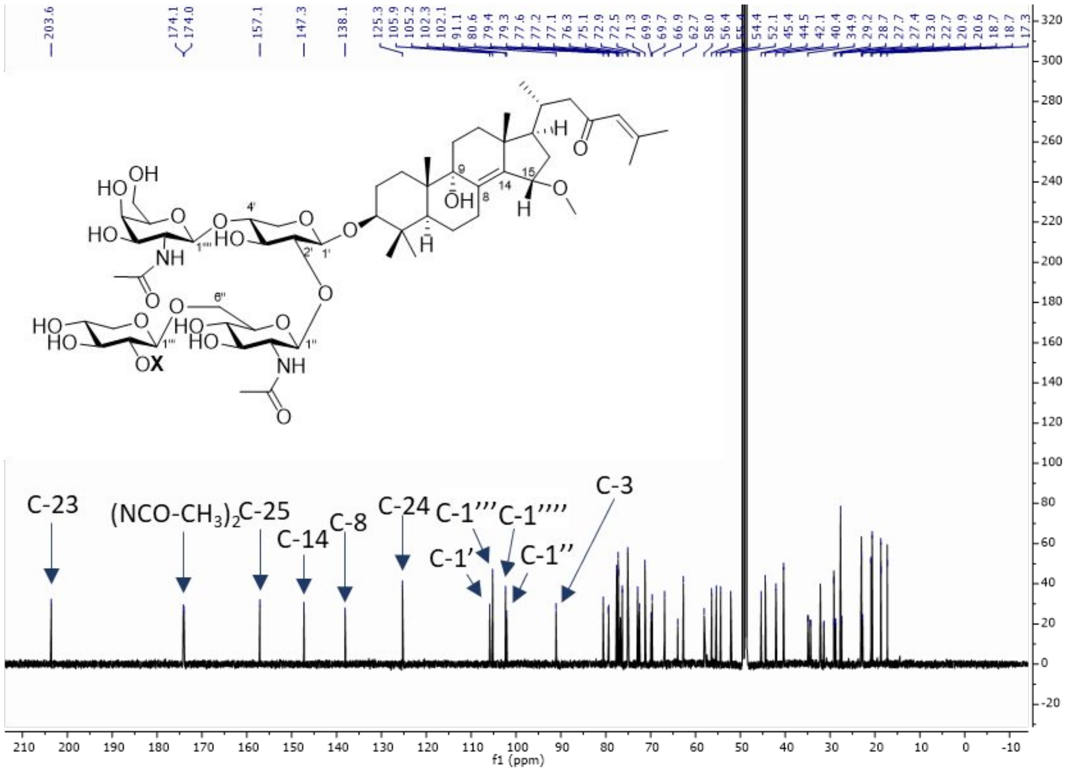

S34: ^{13}C NMR spectrum of **4** (CD_3OD, 125 MHz).

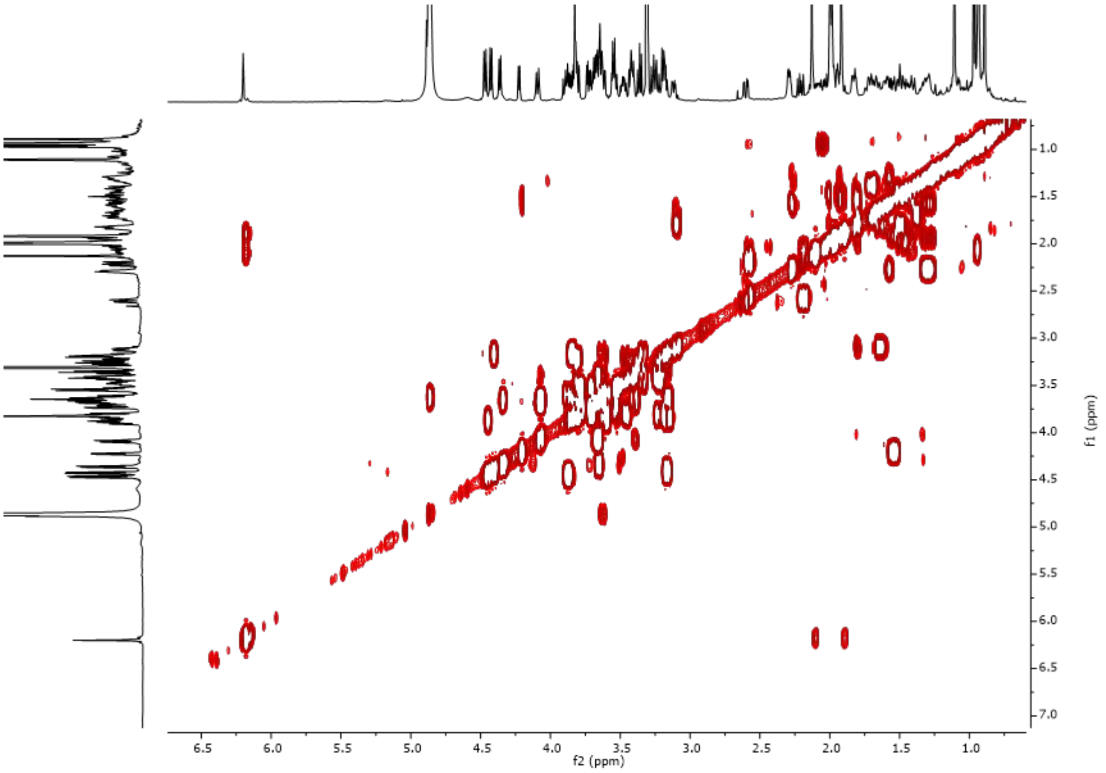

S35: COSY spectrum of **4** (CD_3OD, 500 MHz).

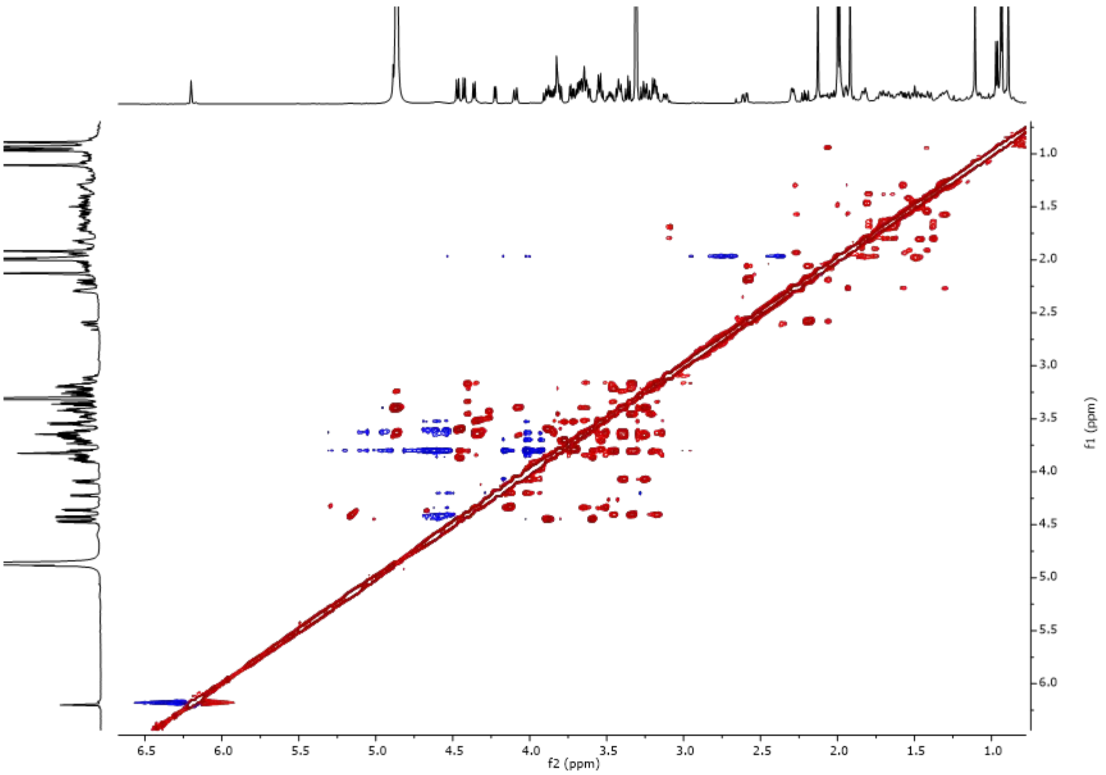

S36: TOCSY spectrum of **4** (CD_3OD, 500 MHz).

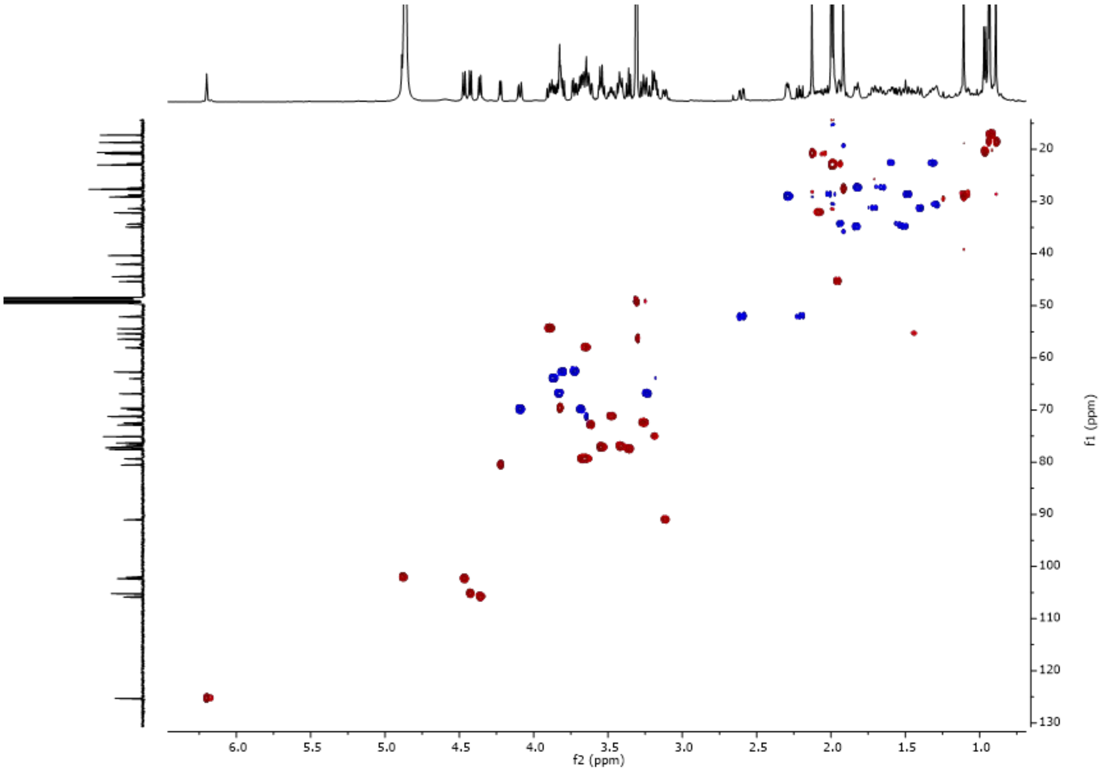

S37: HSQC spectrum of **4** (CD_3OD, 500 MHz).

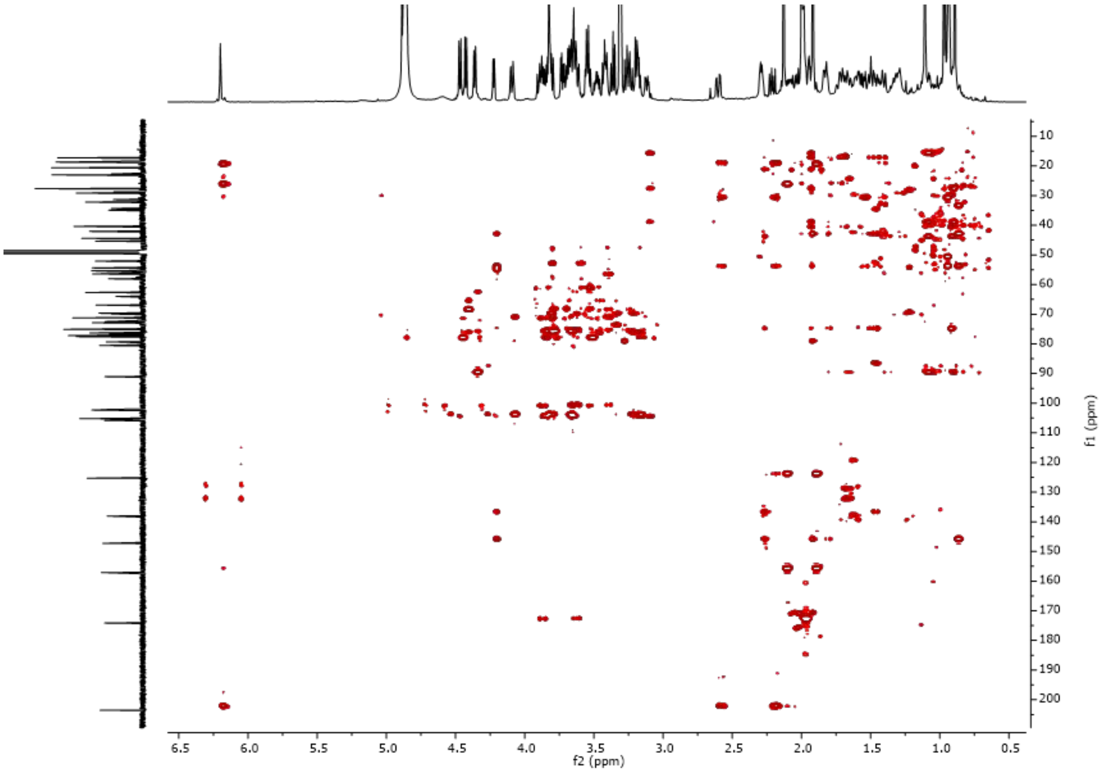

S38: HMBC spectrum of **4** (CD_3OD, 500 MHz).

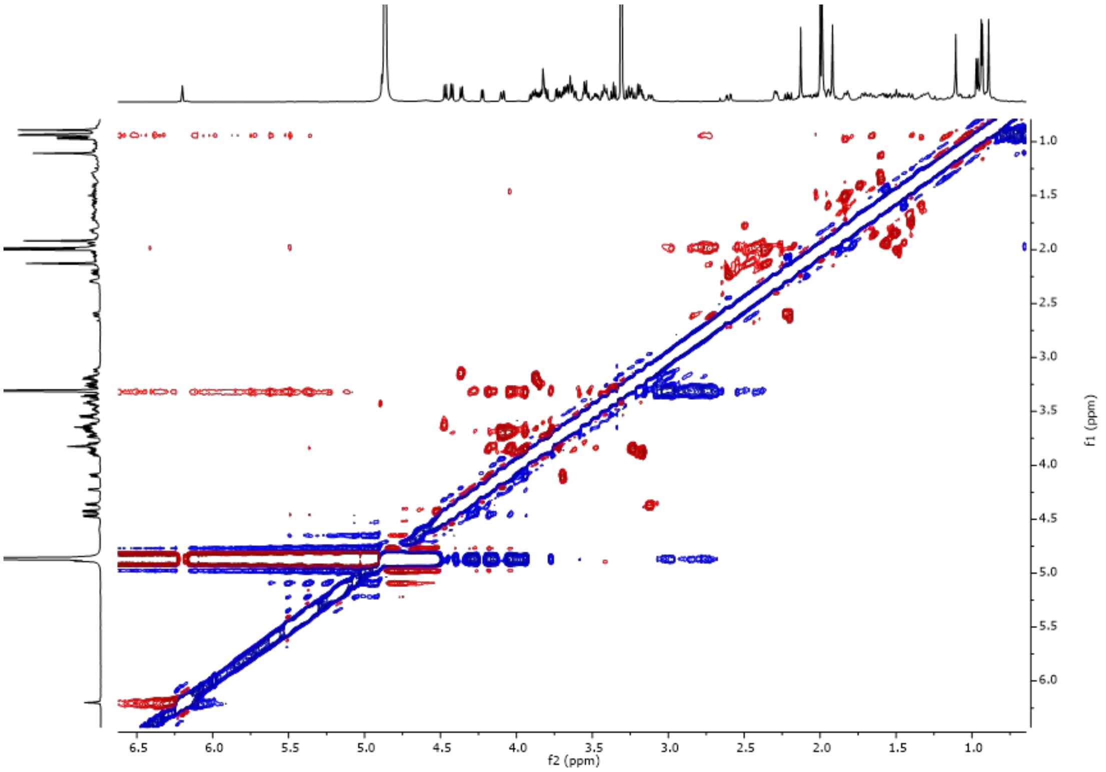

S39: ROESY spectrum of **4** (CD_3OD, 500 MHz).

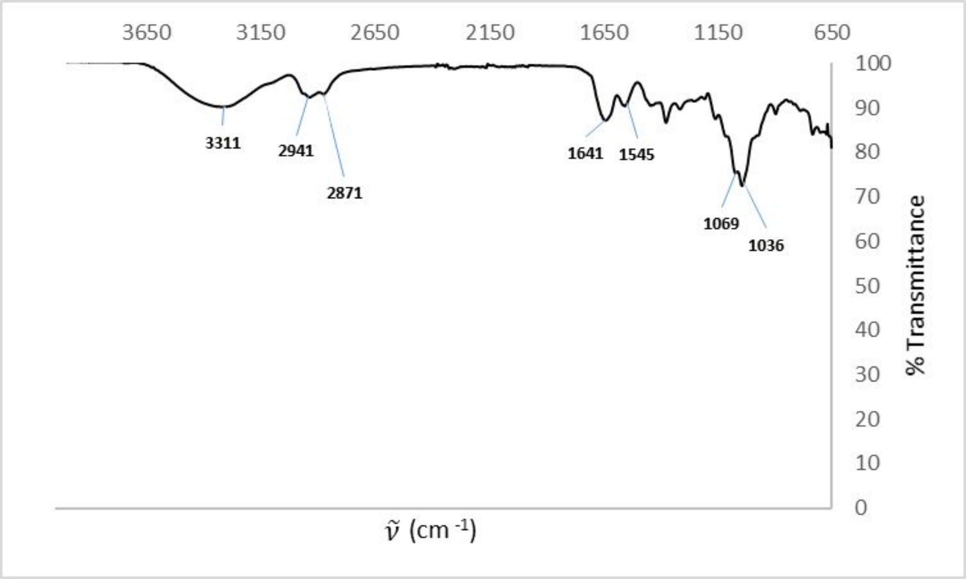

S40: IR spectrum of **4** (Film in a ATR instrument).

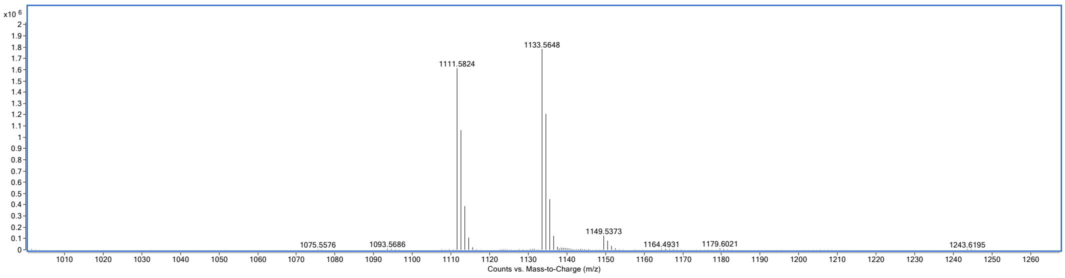

S41: (+)-HRESIMS analysis of sarasinoside C_8 (**5**).

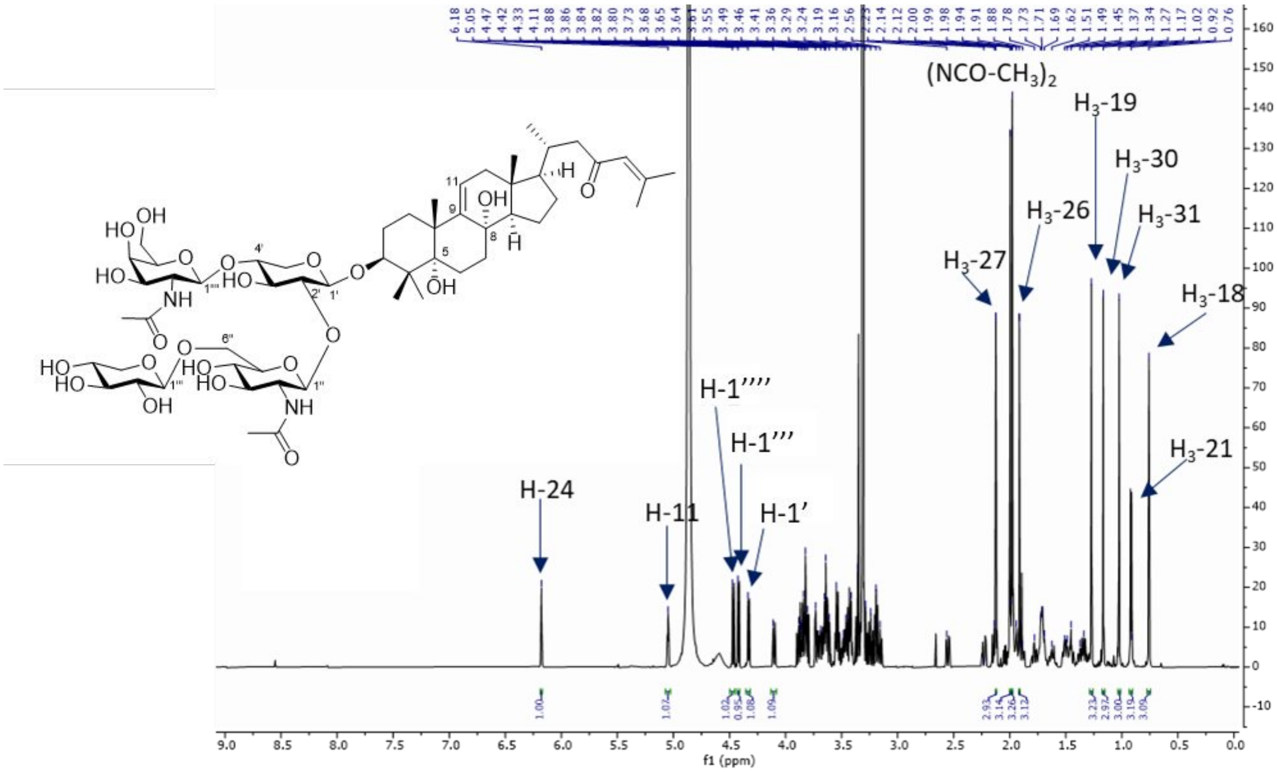

S42: ^{1}H NMR spectrum of **5** (CD_3OD, 600 MHz).

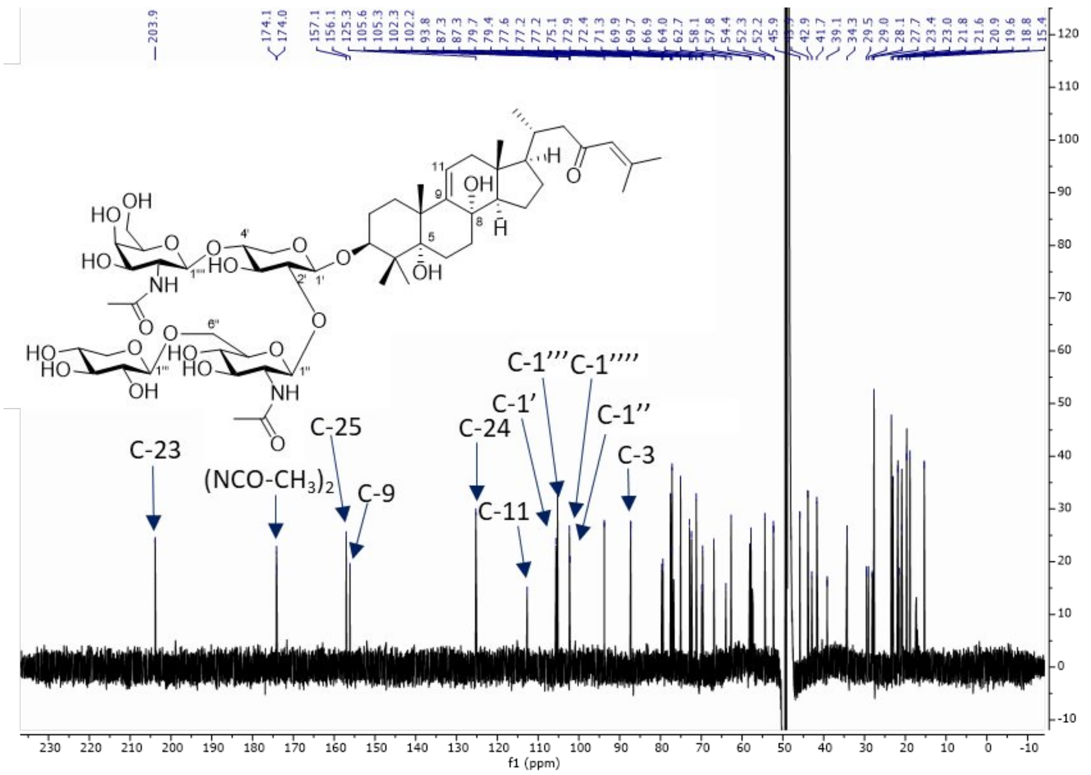

S43: ^{13}C NMR spectrum of **5** (CD_3OD, 150 MHz).

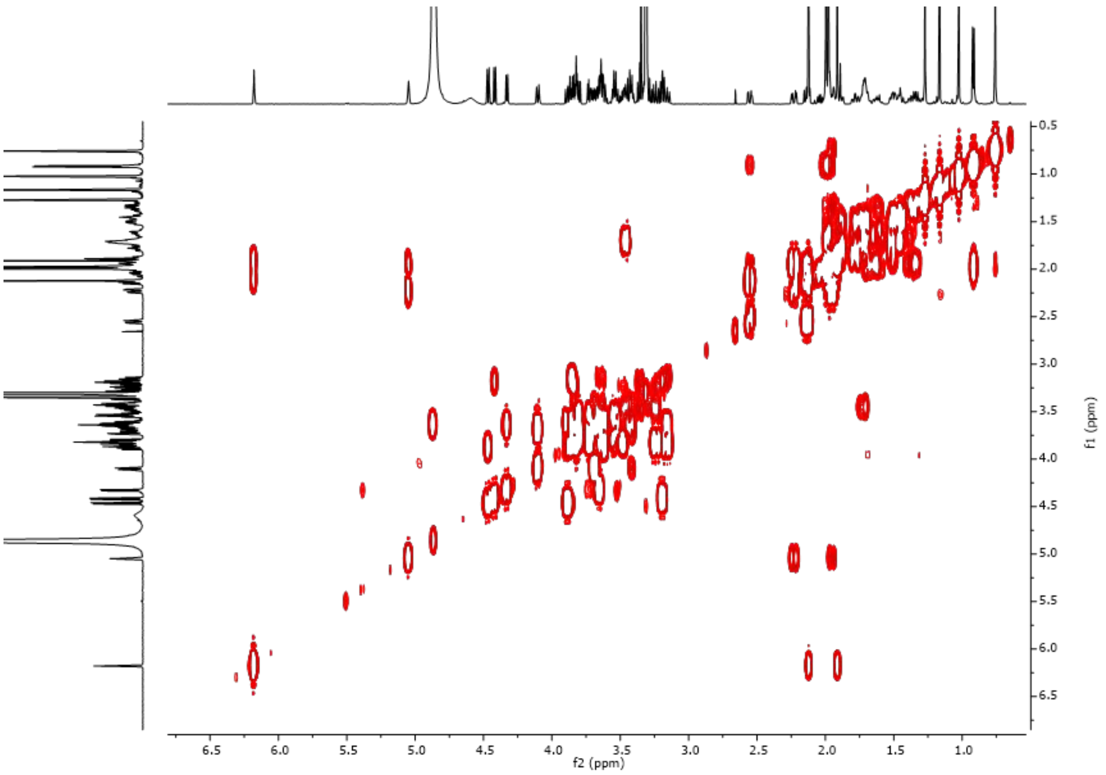

S44: COSY spectrum of **5** (CD_3OD, 600 MHz).

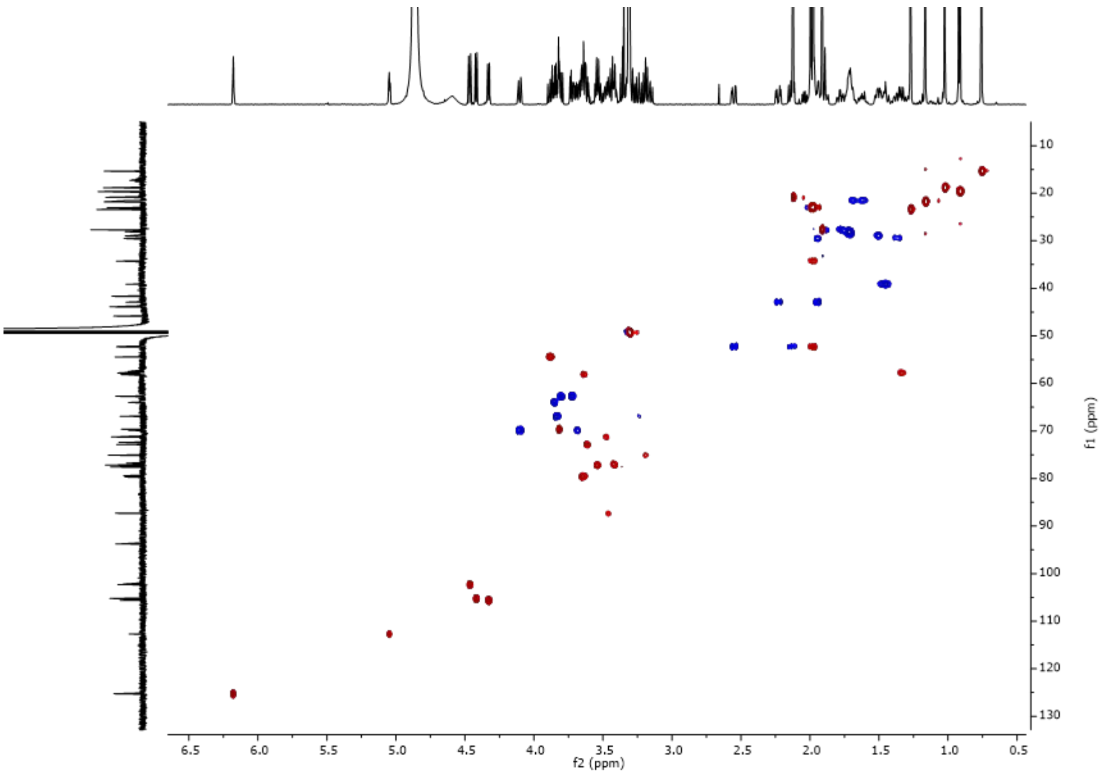

S45: HSQC spectrum of **5** (CD_3OD, 600 MHz).

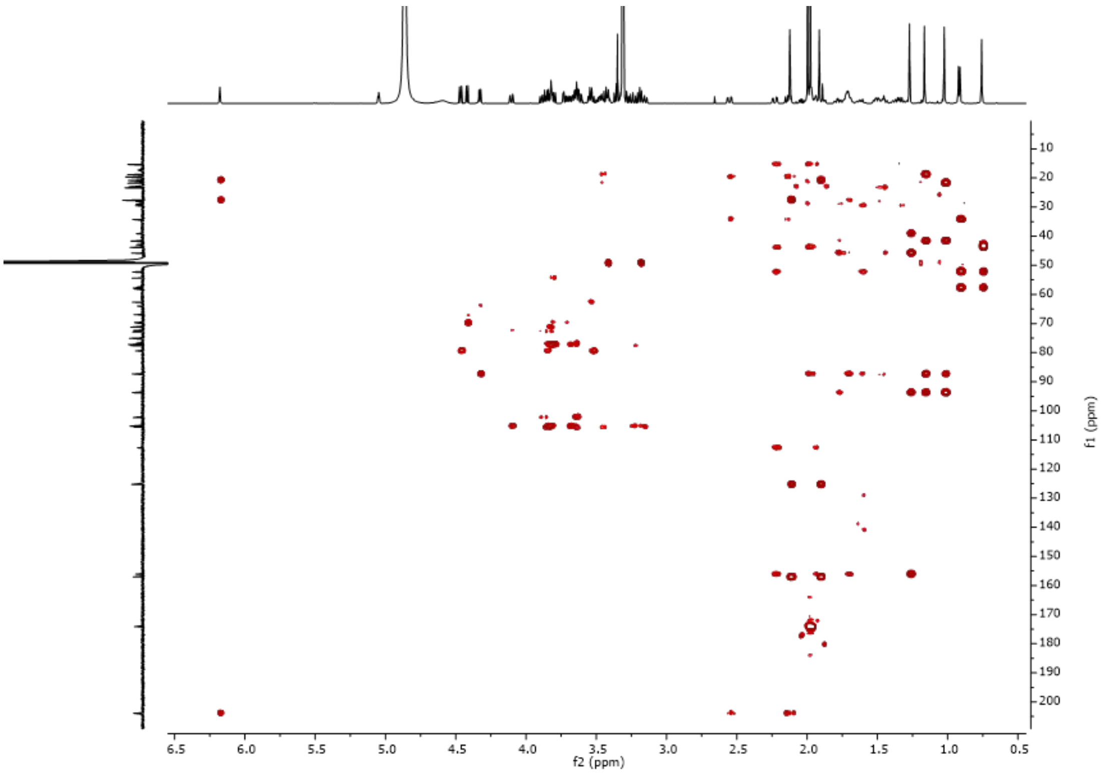

S46: HMBC spectrum of **5** (CD_3OD, 600 MHz).

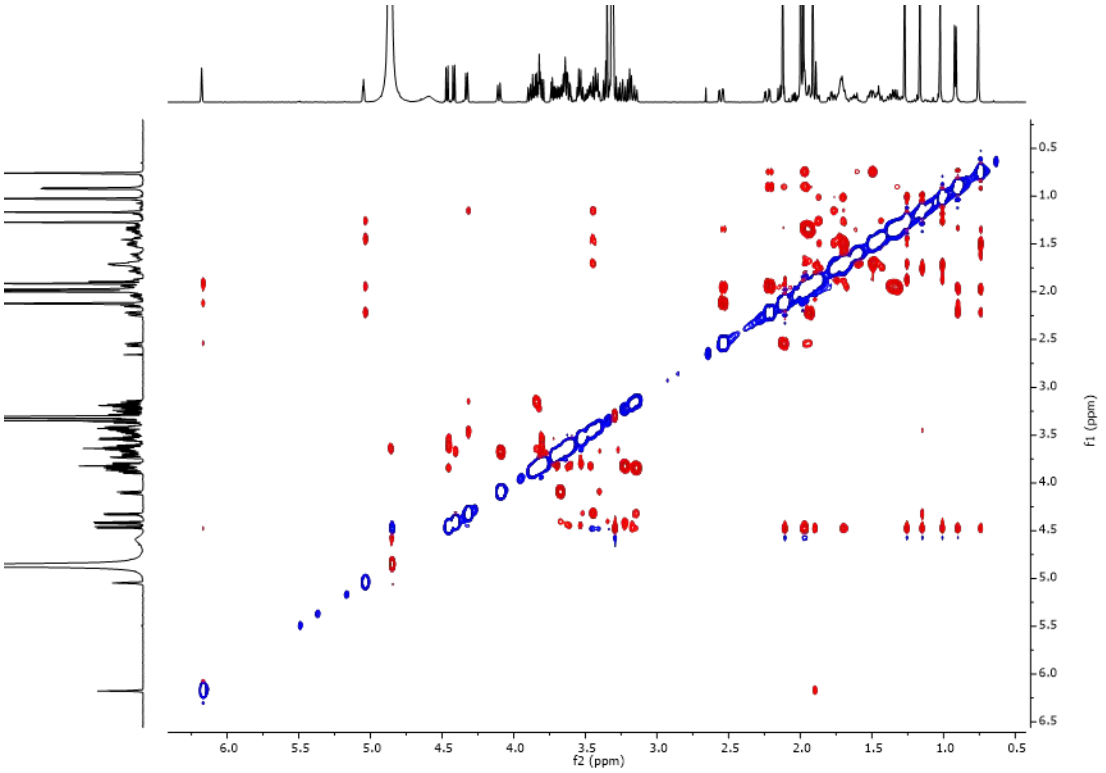

S47: ROESY spectrum of **5** (CD_3OD, 600 MHz).

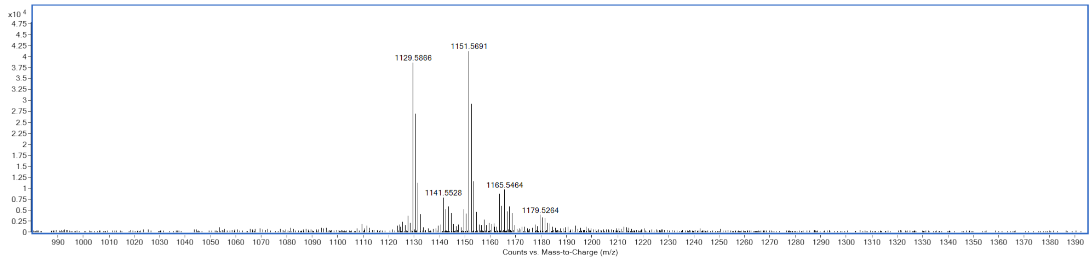

S48: (+)-HRESIMS analysis of Sarasinoside C_9 (**6**).

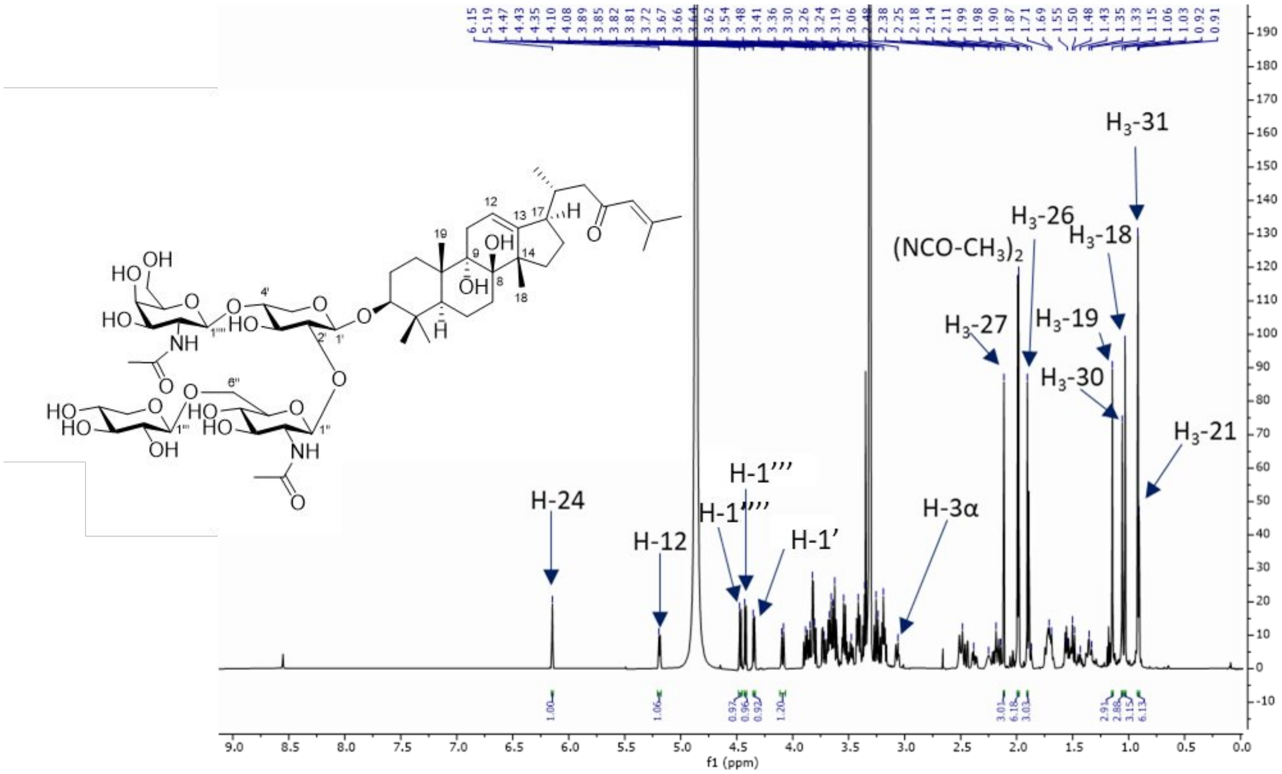

S49: ^{1}H NMR spectrum of **6** (CD_3OD, 600 MHz).

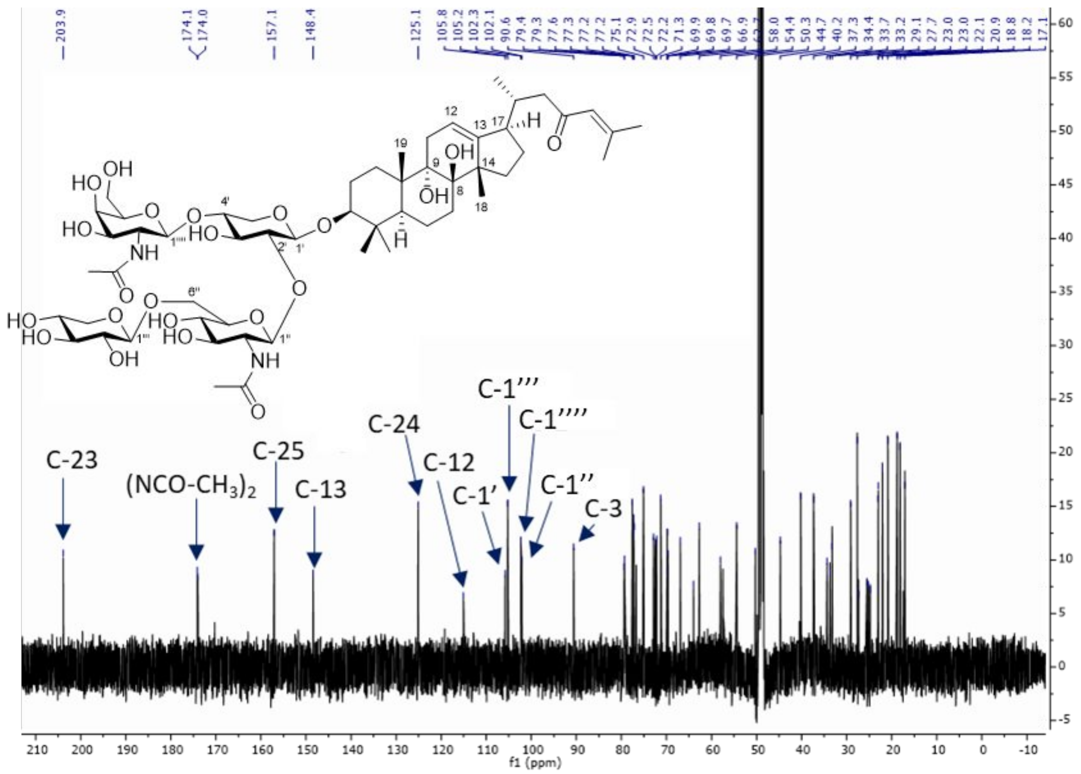

S50: ^{13}C NMR spectrum of **6** (CD_3OD, 150 MHz).

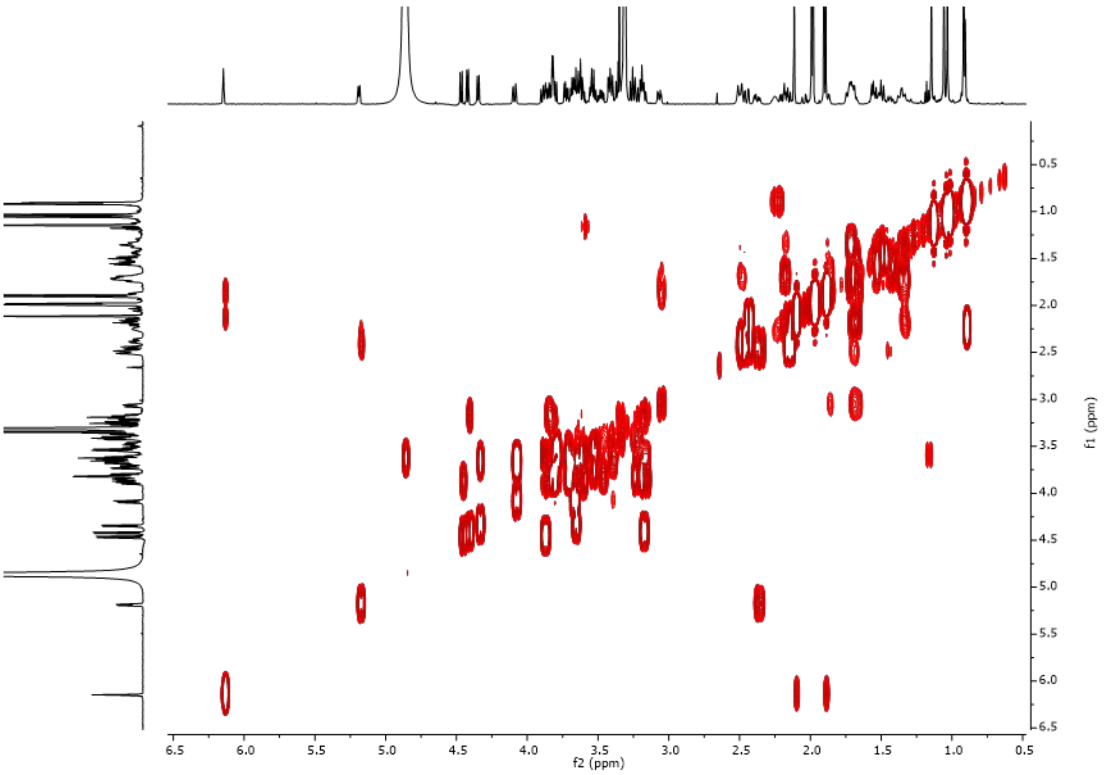

S51: COSY spectrum of **6** (CD_3OD, 600 MHz).

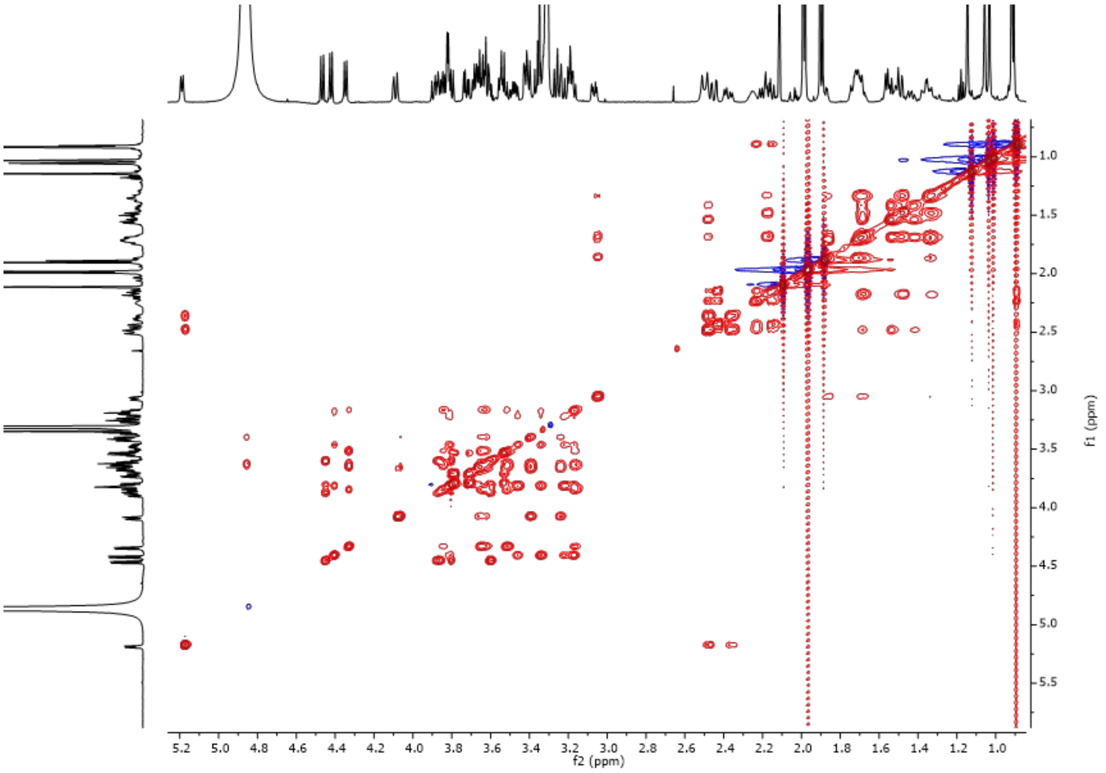

S52: TOCSY spectrum of **6** (CD_3OD, 600 MHz).

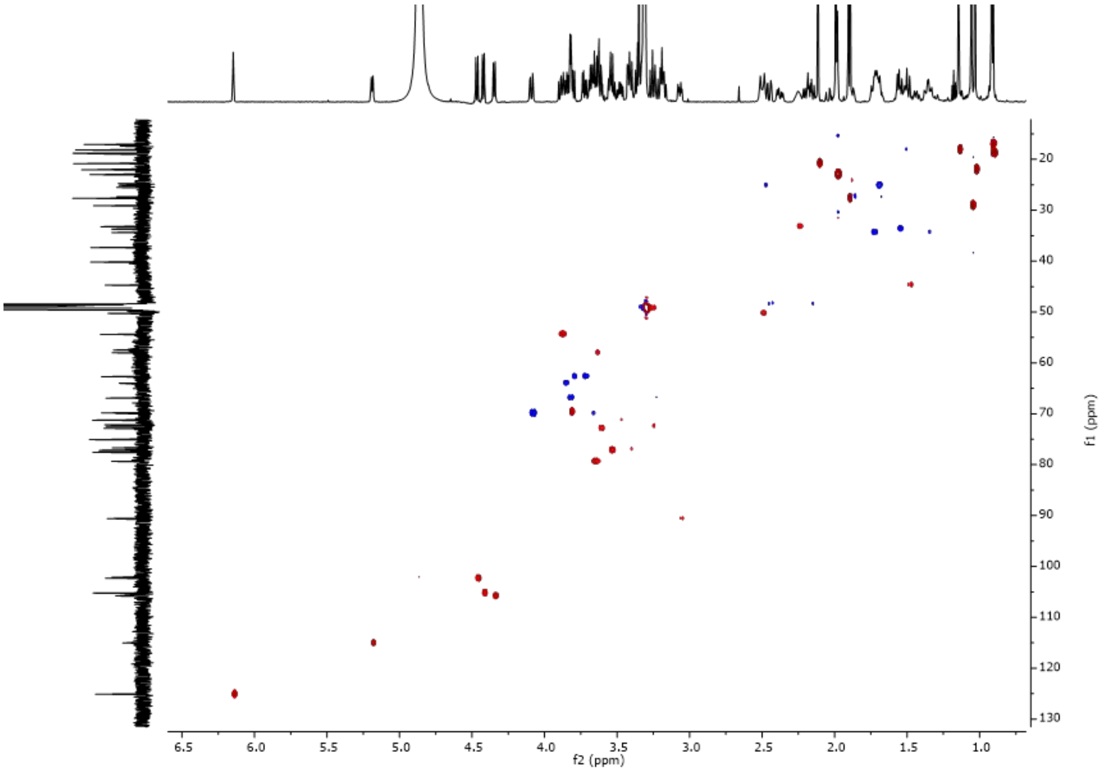

S53: HSQC spectrum of **6** (CD_3OD, 600 MHz).

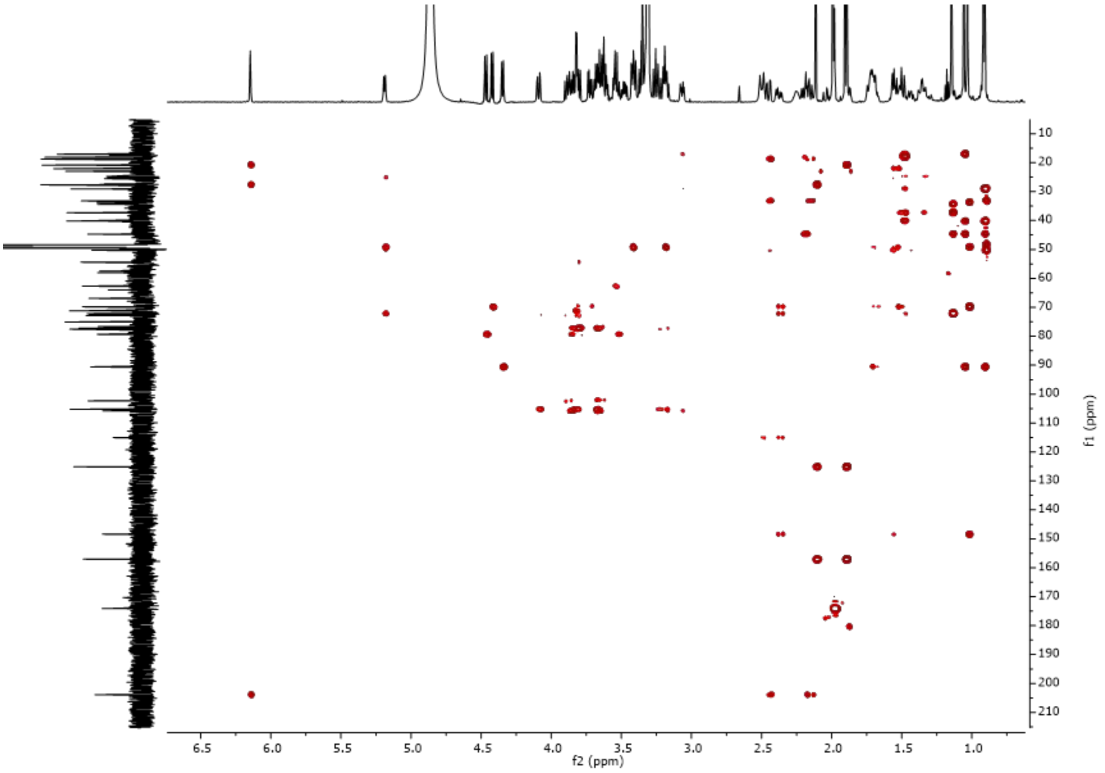

S54: HMBC spectrum of **6** (CD_3OD, 600 MHz).

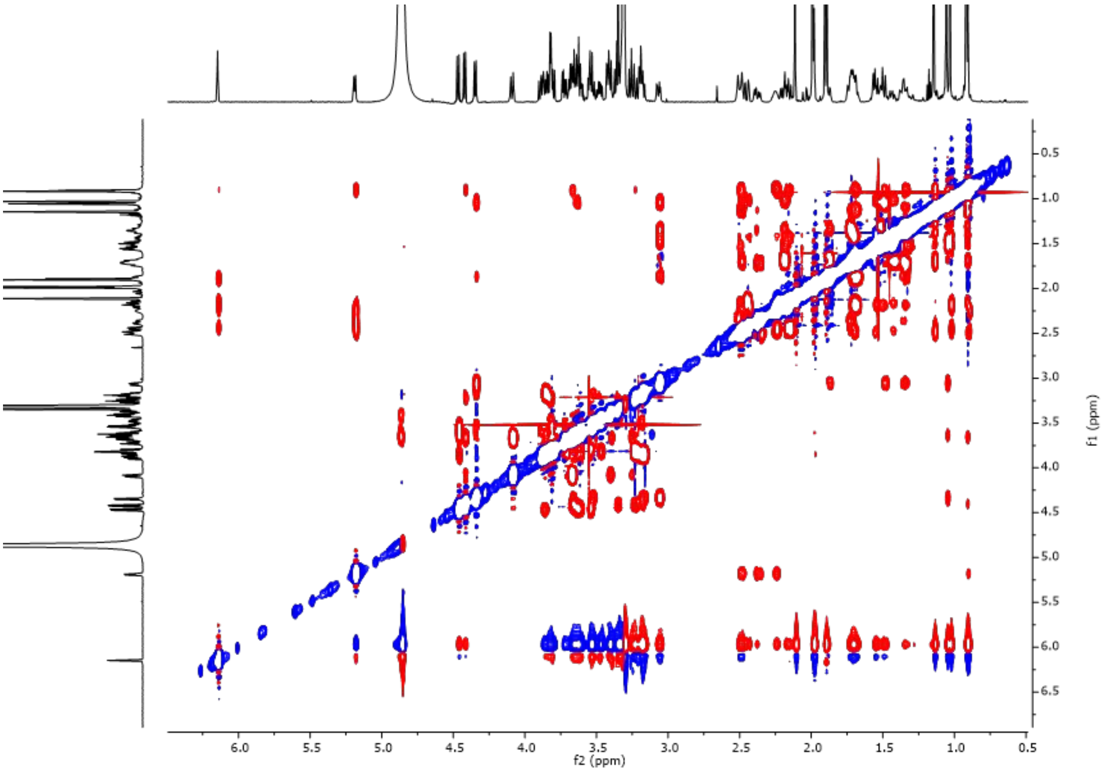

S55: ROESY spectrum of **6** (CD_3OD, 600 MHz).

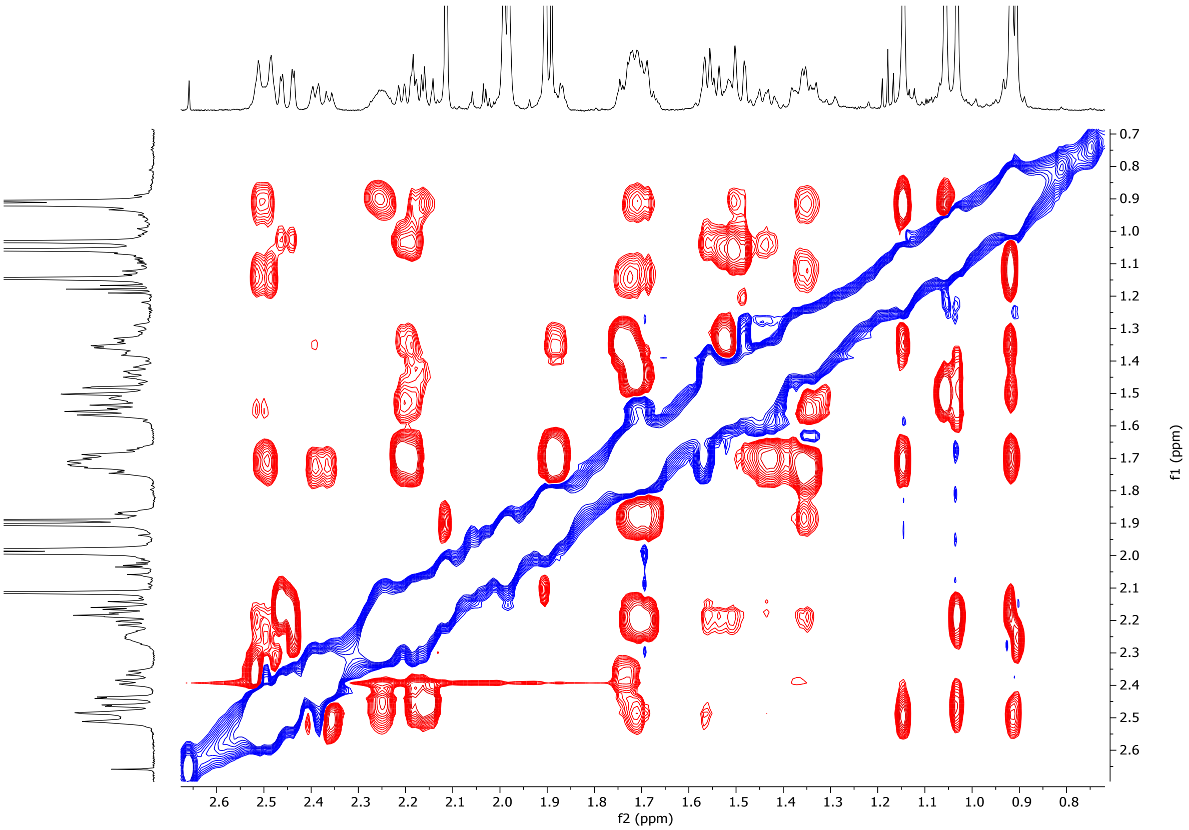

S56: Expansion of the ROESY spectrum of **6** (CD_3OD, 600 MHz) between δ_H 0.7-2.7 ppm.

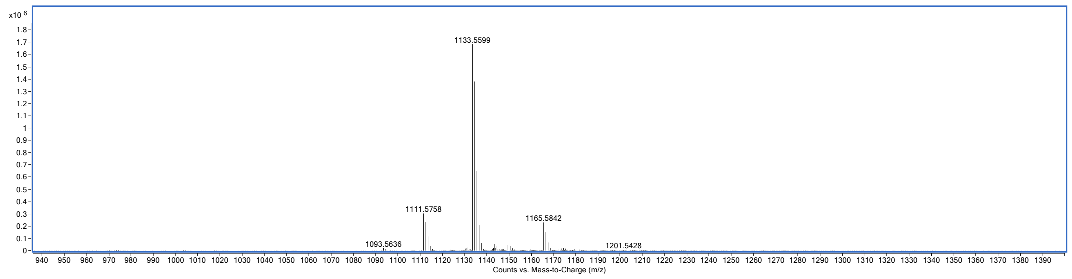

S57: (+)-HRESIMS analysis of Sarasinoside C_9 (**7**).

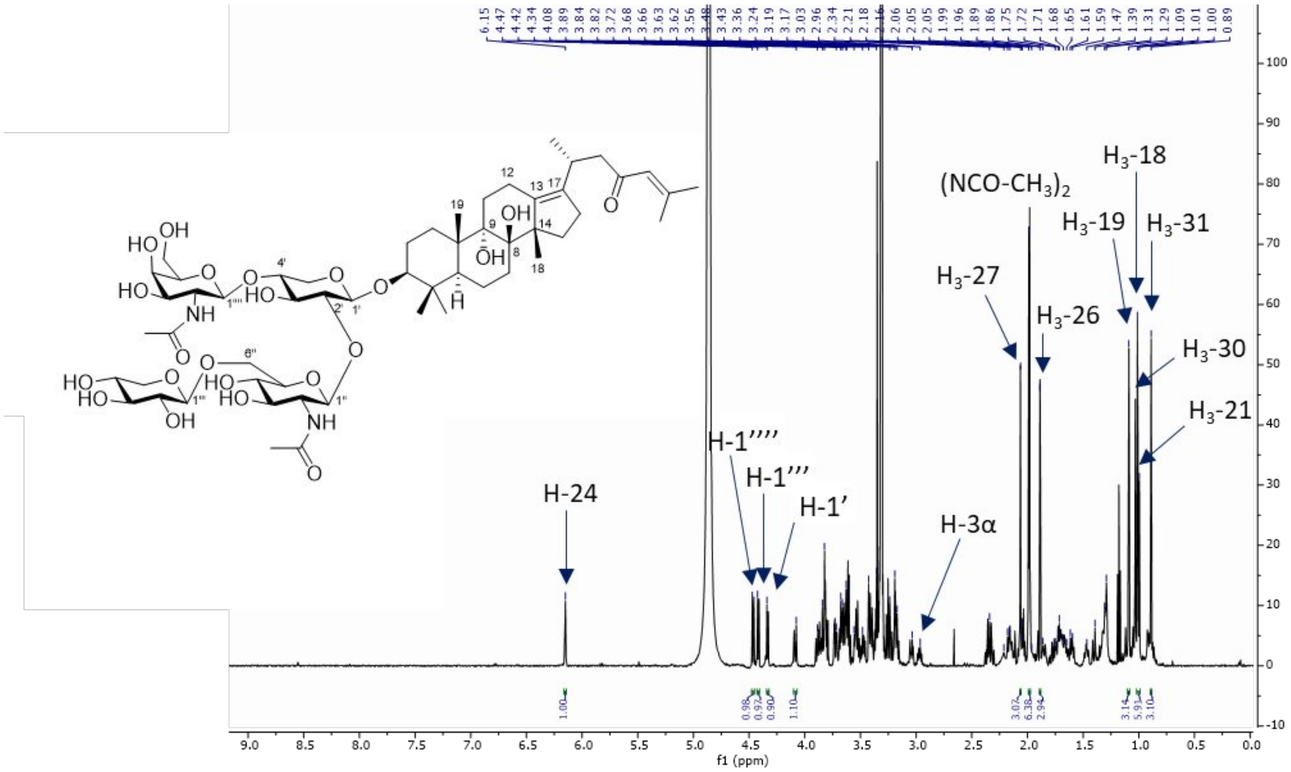

S58: ^{1}H NMR spectrum of **7** (CD_3OD, 600 MHz).

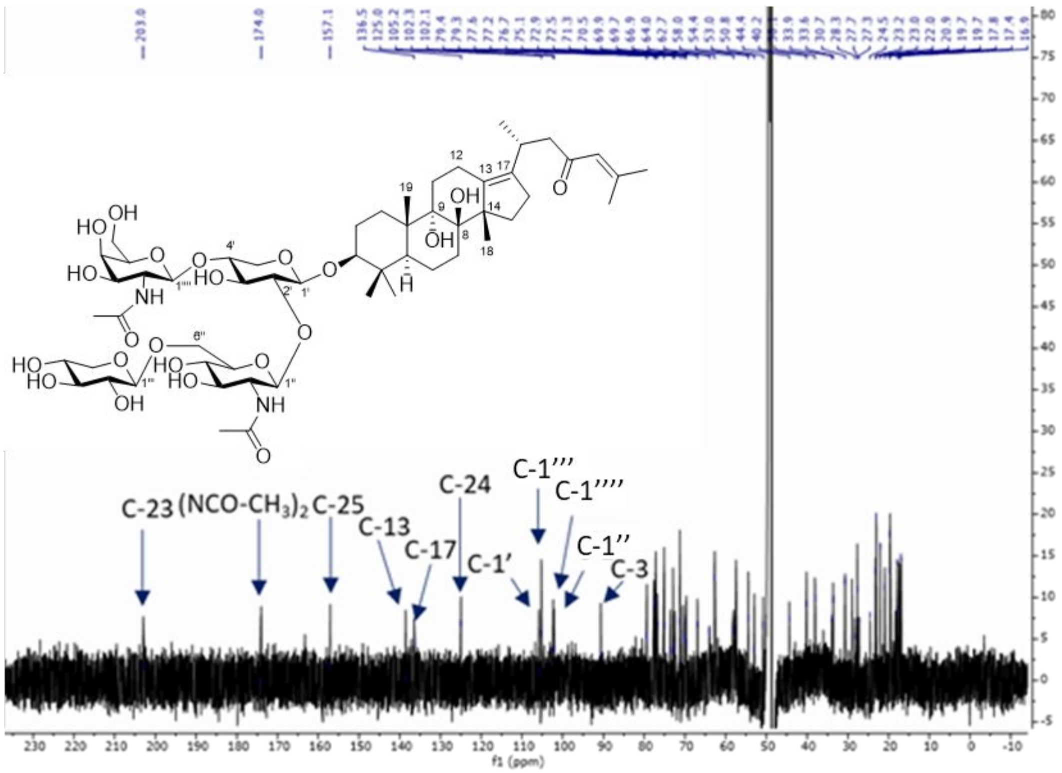

S59: ^{13}C NMR spectrum of 7 (CD_3OD, 150 MHz).

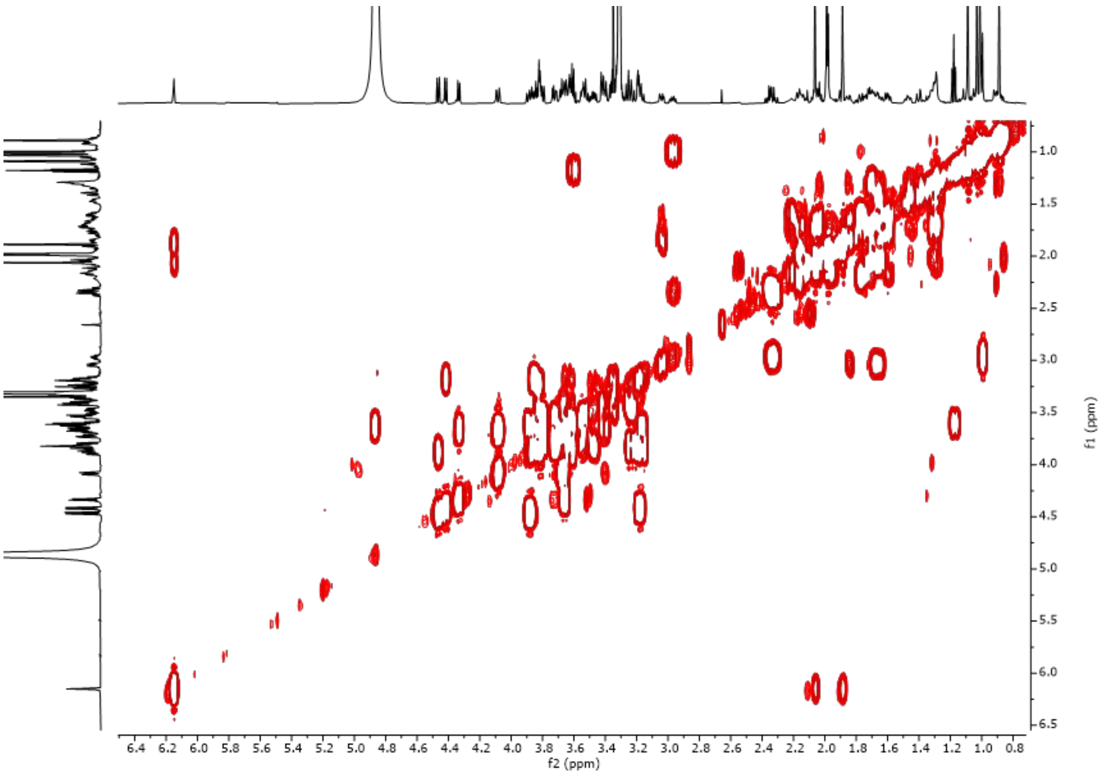

S60: COSY spectrum of 7 (CD_3OD, 600 MHz).

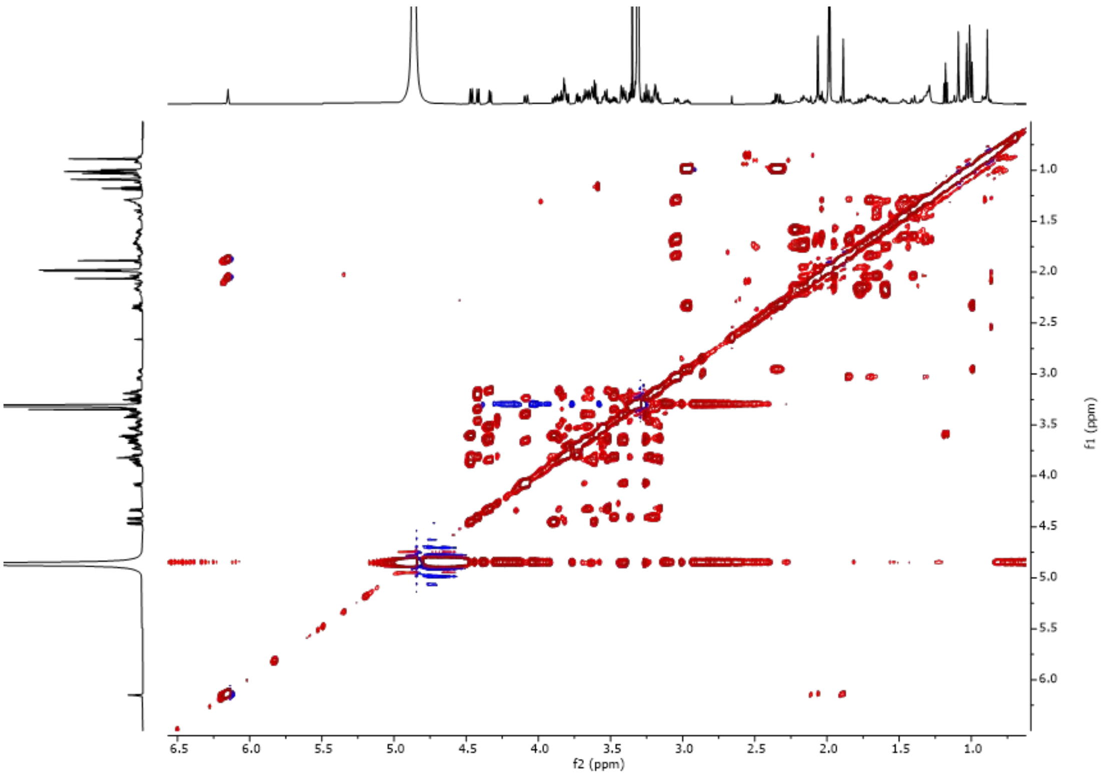

S61: TOCSY spectrum of 7 (CD_3OD, 600 MHz).

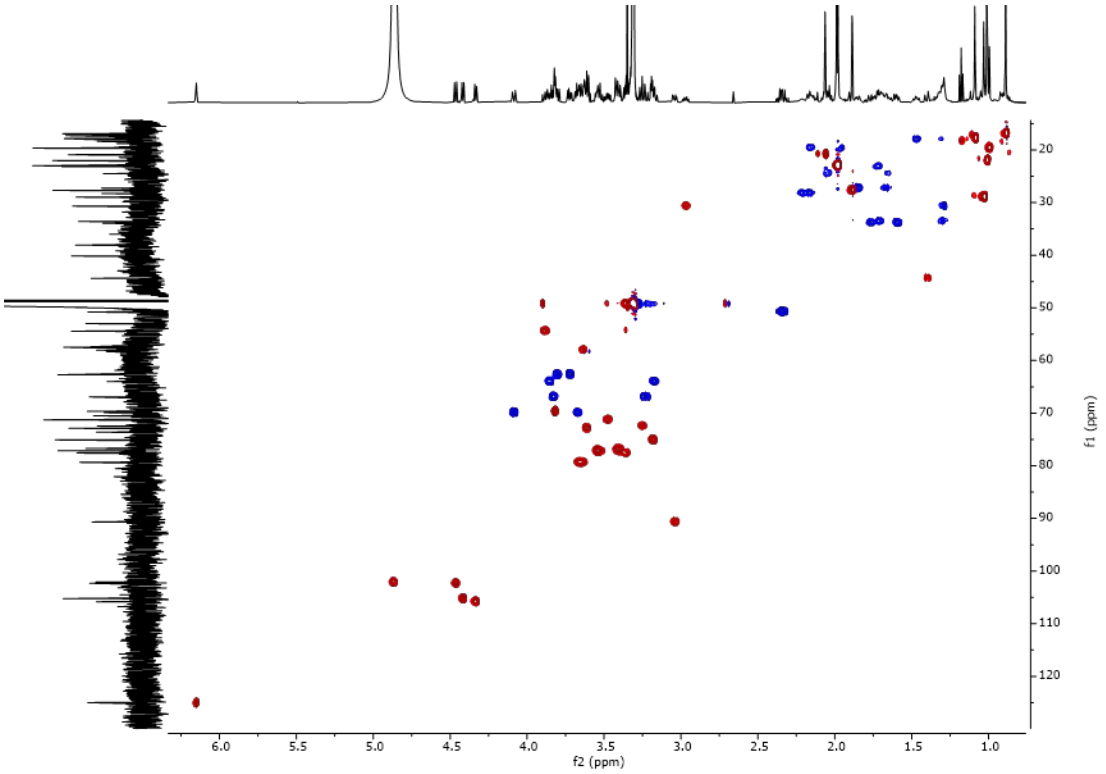

S62: HSQC spectrum of 7 (CD_3OD, 600 MHz).

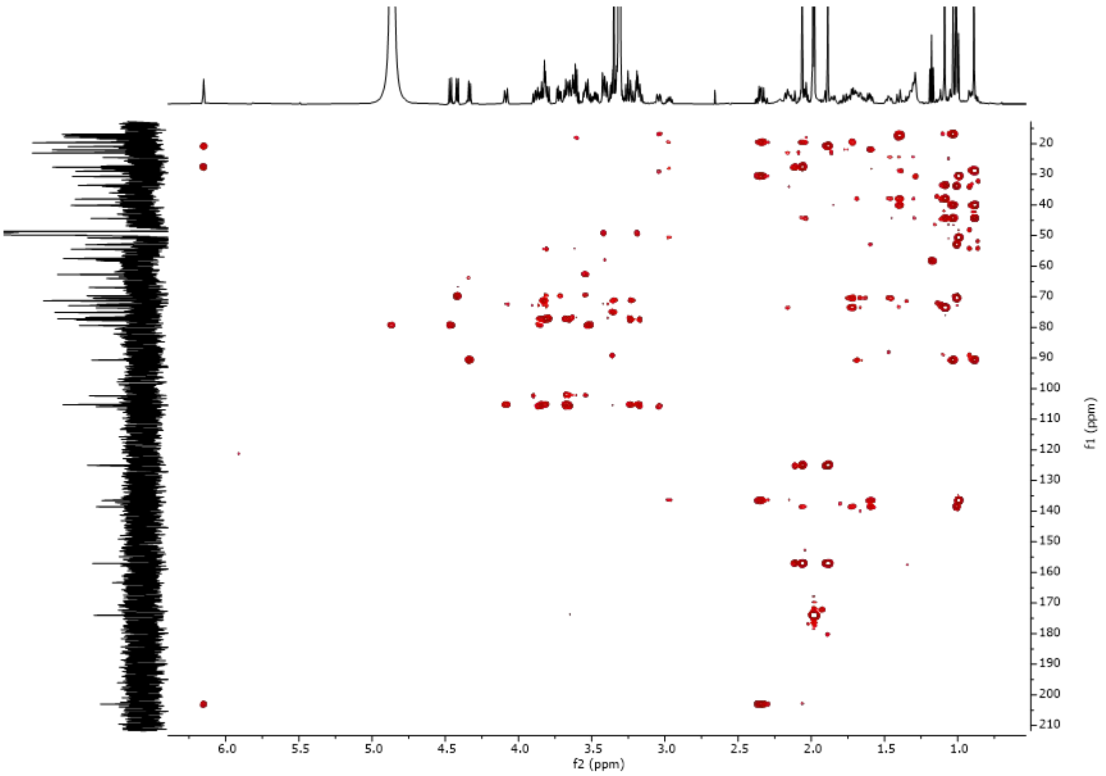

S63: HMBC spectrum of 7 (CD_3OD, 600 MHz).

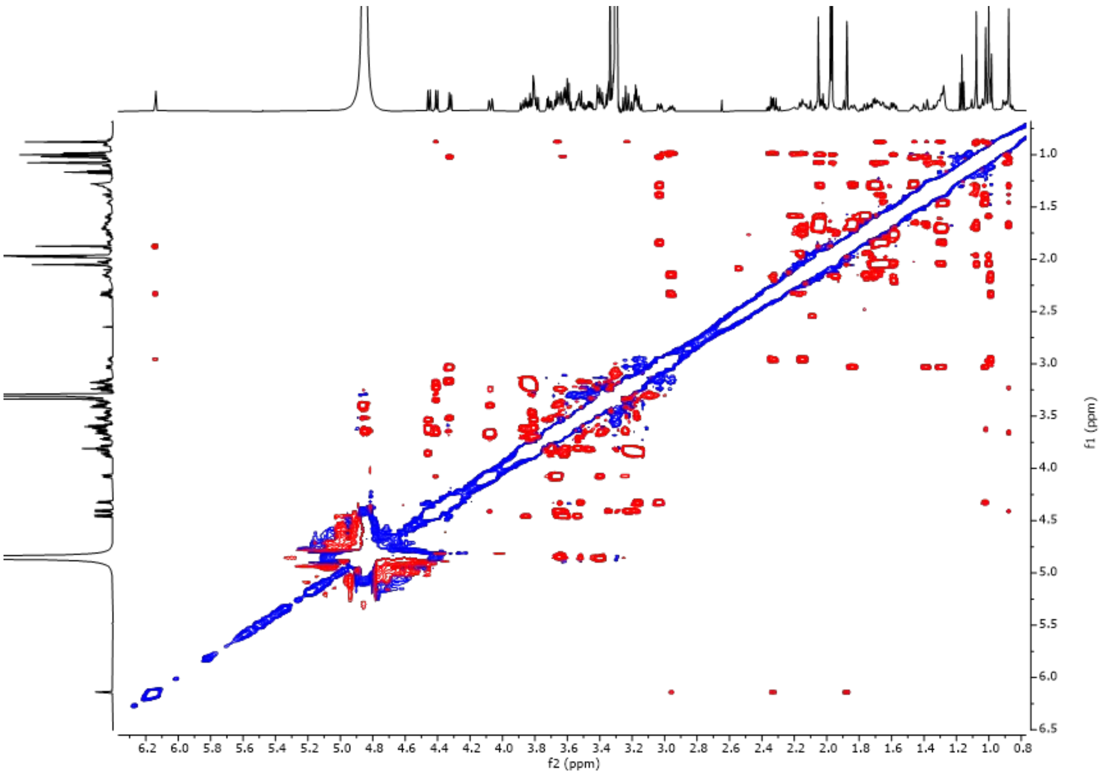

S64: ROESY spectrum of 7 (CD_3OD, 600 MHz).

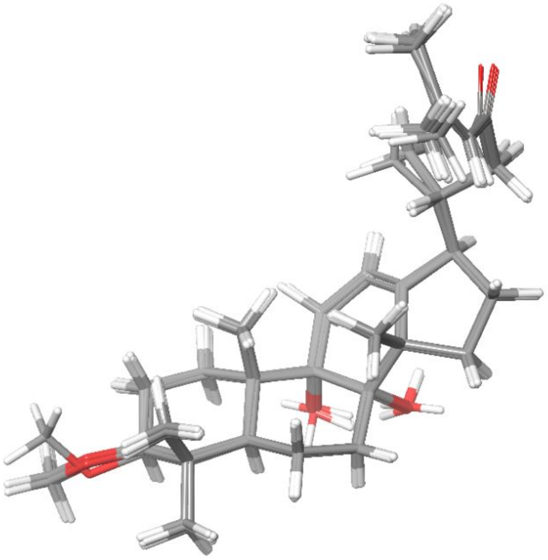

S65: Overlaid 3D structures, cartesian coordinates, number of imaginary frequencies, and energy for Sarasinoside R (8a) cis-8*S,9*S** aglycone conformers.**

cis-8*S,9*S** aglycone - Conformer 1**

Atom	X	Y	Z	Atom	X	Y	Z
C	-	1.194198	0.832669	H	-	0.520395	2.046034
C	-	-	0.680918	H	-	-	1.895334
C	-	-	-	H	-1.22908	-1.81688	2.776674
C	-	0.110276	-	H	1.241376	-	-
C	-	1.314653	-	H	-	-	-
C	-	1.252371	-	H	-	-	-
C	-	-	1.925449	H	3.075742	-	-
C	-	-	1.882057	H	3.289311	-1.47417	1.82526
C	-	-	0.642876	H	3.402886	-	1.35042
C	-	-	-	H	1.057616	-	1.499199
C	0.516649	-	0.812223	H	1.225143	-	2.750109
C	1.254275	-	-	H	-	0.796621	-
C	0.645463	-	-	H	-	1.935297	-
C	-	-	-	H	-	1.563023	0.284777
C	2.738964	-	-	H	3.377251	0.212649	0.137144
C	2.809154	-2.24831	1.223744	H	5.437052	-1.73148	-1.01311
C	1.355272	-2.46325	1.683408	H	5.218862	-	0.708446
C	-1.42351	1.12729	-	H	3.733826	-0.80764	-
C	3.648825	-	-	H	2.468922	0.291686	-
C	5.121119	-	-	H	4.154789	0.807121	-
C	3.489343	-	-	H	5.240822	0.994288	1.428309
C	6.066797	0.210489	-	H	6.892612	3.948736	2.031082
C	5.859793	1.275026	0.581748	H	5.371427	4.331152	1.247992
C	6.304709	2.54425	0.525569	H	5.434764	3.024292	2.446644
C	5.976088	3.503553	1.631768	H	8.16938	3.195289	-
C	7.123264	3.121511	-	H	7.096259	2.510347	-
C	0.732761	-0.0931	1.460192	H	6.789777	4.1382	-
O	-	-	0.521589	H	0.255941	-	2.438242
O	-	-2.35728	-	H	1.796754	0.067327	1.620688
O	-	2.368339	-	H	0.378638	0.726563	0.852266
C	-5.28931	0.898357	1.996302	H	-	-	-
C	-	2.548167	1.114207	H	-	-	-
C	-	2.196987	-	H	-	1.60372	1.970463
O	6.929906	0.242909	-	H	-	-	1.922298
H	-	-	0.569483	H	-4.79973	0.997693	2.965255
H	-	-	-	H	-	3.293013	1.337891
H	-	0.192157	-	H	-	2.4774	1.978708
H	-	1.332014	-	H	-	2.918171	0.274604
H	-	2.257005	-	H	-	3.084715	-
H	-	0.330419	-	H	-	2.095966	-2.19316
H	-	-	2.819244	H	-	1.314482	-
**NImag**						0	
**Σ Electronic and thermal Free Energy**						-924305.615715283	
**mol Fraction**						0.4327	

cis-8*S,9*S** aglycone - Conformer 2**

Atom	X	Y	Z	Atom	X	Y	Z
C	-	1.30455	0.810504	H	-1.62826	0.565872	2.014887
C	-3.27	0.046915	0.710018	H	-2.64254	-	2.024642
C	-	-	-	H	-	-	2.85065
C	-3.39787	0.014791	-	H	1.15007	-	-
C	-	1.273042	-	H	-	-	-
C	-	1.331786	-	H	-	-	-2.22857
C	-	-	1.949854	H	3.046111	-	-
C	-	-	1.95628	H	3.31236	-	1.76345
C	-	-1.98826	0.717387	H	3.431605	-	1.220219
C	-	-	-	H	1.078872	-	1.377548
C	0.515744	-	0.823784	H	1.276563	-	2.706358
C	1.210944	-	-	H	-	0.580964	-
C	0.574816	-	-	H	-	1.761635	-
C	-	-	-	H	-	1.487377	0.17126
C	2.707994	-	-	H	3.318727	0.1971	0.125433
C	2.824315	-	1.144833	H	5.379984	-	-
C	1.383311	-	1.627545	H	5.205743	-	0.580758
C	-	0.988846	-	H	3.603725	-0.72483	-
C	3.581856	-	-	H	2.325199	0.3151	-
C	5.069741	-	-	H	3.995241	0.884328	-
C	3.361427	0.010675	-	H	5.207041	0.943488	1.409647
C	5.984392	0.272241	-	H	6.832361	3.884536	2.124286
C	5.79321	1.280068	0.560103	H	5.282885	4.287655	1.41009
C	6.216903	2.55774	0.560222	H	5.400343	2.916778	2.530576
C	5.909897	3.448894	1.728101	H	6.951077	2.643666	-
C	6.990963	3.209153	-	H	6.629694	4.228022	-
C	0.76279	-	1.498109	H	8.04375	3.29098	-
O	-	-	0.650747	H	0.332828	-	2.49861
O	-	-	-	H	1.831783	-	1.614822
O	-	2.497139	-	H	0.380272	0.649769	0.931642
C	-	1.133461	2.015074	H	-	-	0.218209
C	-	2.633791	0.991396	H	-	-	-0.14621
C	-	2.35783	-	H	-	1.880184	1.963999
O	6.811584	0.368976	-	H	-	0.142611	2.016843
H	-	-	0.690933	H	-	1.266733	2.962528
H	-	-	-1.83241	H	-	3.427448	1.196243
H	-	0.012142	-	H	-	2.577289	1.837088
H	-	1.274972	-	H	-	2.926231	0.113403
H	-3.65829	2.179925	-	H	-	3.282767	-
H	-	0.443822	-	H	-	2.202331	-
H	-	-	2.85019	H	-	1.521091	-

**NImag**	0
**Σ Electronic and thermal Free Energy**	-924304.126007989
**mol Fraction**	0.0349

cis-8*S,9*S** aglycone - Conformer 3**

Atom	X	Y	Z	Atom	X	Y	Z
C	-	1.279243	0.82747	H	-	0.511417	2.037881
C	-	0.031006	0.693449	H	-2.67846	-	1.937087
C	-	-	-	H	-	-	2.805516
C	-	0.085425	-	H	1.1689	-	-
C	-	1.336393	-	H	-	-	-
C	-5.06829	1.345111	-	H	-	-2.7215	-
C	-	-	1.936576	H	3.046518	-	-
C	-	-	1.908716	H	3.278484	-	1.765204
C	-	-	0.66488	H	3.420445	-	1.17813
C	-	-	-0.64593	H	1.083599	-	1.263159
C	0.501537	-	0.796697	H	1.239531	-	2.652618
C	1.209161	-	-	H	-	0.633154	-
C	0.583308	-	-	H	-	1.825819	-
C	-	-	-	H	-	1.49395	0.226516
C	2.704436	-	-	H	3.315815	0.194105	0.163516
C	2.807604	-	1.117924	H	5.377875	-	-
C	1.361597	-	1.570092	H	5.196969	-	0.613677
C	-	1.02757	-	H	3.632005	-	-2.75168
C	3.584656	-0.55946	-	H	2.34568	0.36086	-
C	5.068859	-	-	H	4.015339	0.930777	-
C	3.380274	0.053438	-1.96621	H	5.205319	0.958516	1.433342
C	5.993949	0.266775	-	H	6.832348	3.906636	2.124199
C	5.798619	1.284603	0.584648	H	5.287334	4.302055	1.396163
C	6.224884	2.561366	0.572964	H	5.397198	2.944916	2.534013
C	5.911843	3.46682	1.728089	H	6.652218	4.217734	-
C	7.007034	3.198384	-	H	8.059191	3.276571	-
C	0.746733	-	1.520542	H	6.96786	2.624786	-
O	-	-	0.486452	H	0.319249	-	2.522282
O	-	-	-	H	1.815089	-	1.638621
O	-	2.503045	-	H	0.356582	0.616725	0.984766
C	-	1.061433	2.011632	H	-	-	1.354725
C	-	2.606061	1.063887	H	-	-	-
C	-	2.367095	-	H	-	1.803911	1.97596
O	6.8327	0.349951	-	H	-	0.067469	1.972122
H	-	-	0.611635	H	-	1.165407	2.970922
H	-	-	-	H	-4.19314	3.392096	1.279043
H	-	0.119615	-	H	-	2.525684	1.920395
H	-4.90283	1.365896	-	H	-	2.926178	0.206607
H	-	2.249348	-	H	-	3.286194	-
H	-	0.451703	-0.44892	H	-	2.228431	-
H	-	-	2.833809	H	-	1.520746	-

**NImag**	0
**Σ Electronic and thermal Free Energy**	-924304.309868241
**mol Fraction**	0.0476

cis-8*S,9*S** aglycone - Conformer 4**

Atom	X	Y	Z	Atom	X	Y	Z
C	-	1.303509	0.811639	H	-	0.573105	2.017946
C	-	0.047938	0.709296	H	-	-	2.030595
C	-	-	-	H	-1.20435	-	2.858276
C	-	0.026622	-1.79571	H	1.135087	-	-
C	-	1.287611	-	H	-	-	-
C	-	1.339864	-	H	-	-	-
C	-	-0.1814	1.951463	H	3.039613	-	-
C	-	-	1.965399	H	3.316426	-	1.750787
C	-0.99314	-	0.726742	H	3.430865	-	1.197823
C	-	-	-	H	1.07919	-	1.365393
C	0.514843	-	0.822148	H	1.282548	-	2.697526
C	1.204038	-	-	H	-	0.581766	-
C	0.564724	-	-	H	-1.65975	1.769596	-
C	-	-1.81797	-	H	-	1.48886	0.171289
C	2.702335	-	-	H	3.310271	0.194386	0.121344
C	2.824512	-2.3278	1.130314	H	5.370626	-	-
C	1.385242	-	1.617891	H	5.201867	-1.38876	0.559911
C	-	0.992926	-	H	3.583641	-	-
C	3.571873	-0.57029	-	H	2.306502	0.321544	-
C	5.061275	-	-	H	3.975501	0.893432	-
C	3.343844	0.018049	-	H	5.199556	0.932696	1.40639
C	5.972847	0.278648	-	H	6.824514	3.867289	2.144499
C	5.783433	1.277208	0.55844	H	5.273833	4.277023	1.436719
C	6.206398	2.555091	0.569219	H	5.393181	2.895794	2.544211
C	5.901369	3.435345	1.745851	H	6.614137	4.23631	-
C	6.977894	3.217015	-	H	8.030721	3.299038	-
C	0.769237	-	1.499504	H	6.938373	2.658826	-
O	-	-	0.665095	H	0.338531	-	2.499472
O	-	-	-0.58966	H	1.838858	-	1.616796
O	-	2.505879	-	H	0.389858	0.643014	0.935671
C	-	1.124	2.008139	H	-	-	0.310113
C	-	2.63295	1.004898	H	-	-2.68824	-
C	-	2.365998	-	H	-	1.869752	1.957593
O	6.79642	0.384269	-	H	-	0.131909	1.999354
H	-3.96085	-	0.663702	H	-	1.251282	2.960291
H	-	-	-	H	-	3.428173	1.202758
H	-	0.017237	-2.7348	H	-	2.573401	1.858288
H	-	1.295642	-2.60625	H	-	2.926077	0.134632
H	-	2.192132	-	H	-	3.291278	-
H	-5.70835	0.45229	-	H	-	2.208628	-
H	-3.00765	-	2.850897	H	-	1.530049	-

**NImag**	0
**Σ Electronic and thermal Free Energy**	-924304.353166389
**mol Fraction**	0.0512

cis-8*S,9*S** aglycone - Conformer 5**

Atom	X	Y	Z	Atom	X	Y	Z
C	-	1.091701	0.82391	H	-1.77692	0.421264	2.036354
C	-	-	0.663865	H	-2.64084	-2.49856	1.854303
C	-	-	-	H	-1.25077	-1.91751	2.74354
C	-	0.027667	-	H	1.224445	-1.84347	-2.61002
C	-	1.227877	-	H	-1.06699	-0.82687	-2.65229
C	-	1.148161	-	H	-1.15176	-2.5427	-2.47367
C	-	-	1.906477	H	3.072693	-2.60878	-0.96158
C	-	-	1.849899	H	3.26591	-1.51184	1.798261
C	-	-1.99702	0.609195	H	3.403101	-3.18145	1.296538
C	-	-	-	H	1.06336	-3.55529	1.439197
C	0.493631	-	0.782772	H	1.212283	-2.35505	2.708927
C	1.232529	-	-0.53726	H	-0.52888	0.742113	-1.13504
C	0.625671	-	-1.71625	H	-1.85177	1.869216	-1.31153
C	-	-	-	H	-1.25691	1.487044	0.27988
C	2.720597	-	-	H	3.319278	0.206327	0.135122
C	2.79682	-	1.1841	H	5.423283	-1.67102	-1.04455
C	1.345851	-	1.639531	H	5.189625	-1.36775	0.683916
C	-	1.058472	-	H	3.699865	-0.76591	-2.73683
C	3.607712	-0.57494	-	H	2.414305	0.30205	-2.16392
C	5.086974	-	-	H	4.090566	0.847929	-2.14171
C	3.440403	-	-	H	5.158188	0.982865	1.466118
C	6.005087	0.270372	-	H	6.738898	3.955271	2.158248
C	5.77171	1.30194	0.629203	H	5.211087	4.325648	1.382309
C	6.186765	2.582348	0.610546	H	5.301762	2.985985	2.542334
C	5.83373	3.501175	1.743375	H	6.631377	4.223026	-0.68263
C	6.993159	3.210963	-	H	8.035236	3.307001	-0.16085
C	0.692455	-	1.449765	H	6.987387	2.622401	-1.39671
O	-0.97695	-	0.476175	H	0.216402	-0.1347	2.428861
O	-	-	-	H	1.754226	0.011693	1.611613
O	-	2.138856	-	H	0.326737	0.662487	0.853751
C	-	0.776261	1.981603	H	-0.28286	-3.69437	-0.15821
C	-	2.438668	1.138804	H	-2.51076	-3.28885	-0.54685
C	-	3.414741	-	H	-6.16435	1.479335	1.964854
O	6.867967	0.346845	-	H	-5.73623	-0.23527	1.891348
H	-	-	0.544644	H	-4.84305	0.861216	2.952374
H	-	-	-	H	-4.45483	3.170005	1.423335
H	-	0.116058	-	H	-3.01386	2.339215	1.983337
H	-	1.233983	-	H	-3.13377	2.846054	0.302869
H	-	2.163116	-	H	-6.90749	4.014346	-0.74492
H	-	0.2027	-	H	-5.1479	3.914793	-0.57346
H	-	-0.34044	2.799847	H	-5.93054	3.362539	-2.07071
**NImag**						0	
**Σ Electronic and thermal Free Energy**						-924301.442777833	
**mol Fraction**						0.0004	

cis-8*S,9*S** aglycone - Conformer 6**

Atom	X	Y	Z	Atom	X	Y	Z
C	-4.32408	1.194257	0.832631	H	-1.73626	0.520459	2.046068
C	-3.33281	-0.00252	0.680929	H	-2.62573	-2.39419	1.895447
C	-2.45052	-0.02208	-0.60413	H	-1.22908	-1.81676	2.776748
C	-3.39487	0.110242	-1.82711	H	1.241326	-1.83014	-2.57658
C	-4.32758	1.314628	-1.72183	H	-1.03344	-0.77343	-2.62526
C	-5.17069	1.252389	-0.45675	H	-1.14393	-2.48703	-2.43896
C	-2.48387	-0.26158	1.925475	H	3.075752	-2.61347	-0.91638
C	-1.83163	-1.64544	1.882128	H	3.289316	-1.47404	1.825302
C	-0.97879	-1.92049	0.642955	H	3.402904	-3.15335	1.350563
C	-1.74729	-1.43429	-0.6712	H	1.057627	-3.49147	1.499451
C	0.516644	-1.47552	0.812275	H	1.225198	-2.27389	2.750225
C	1.254266	-1.57856	-0.50649	H	-0.48331	0.796609	-1.12201
C	0.645437	-1.65708	-1.68435	H	-1.79469	1.935329	-1.31136
C	-0.83189	-1.57859	-1.9155	H	-1.20559	1.562889	0.284813
C	2.738973	-1.80134	-0.26426	H	3.377232	0.212678	0.137022
C	2.809167	-2.24822	1.223825	H	5.437035	-1.73148	-1.01321
C	1.355284	-2.46313	1.683541	H	5.218856	-1.38139	0.708357
C	-1.42352	1.127261	-0.68397	H	4.154747	0.806802	-2.15024
C	3.648795	-0.58422	-0.56118	H	3.733412	-0.80797	-2.72146
C	5.121115	-0.96821	-0.29943	H	2.468795	0.291655	-2.16182
C	3.489192	-0.04166	-1.98043	H	5.240946	0.994189	1.428253
C	6.06676	0.210514	-0.41389	H	6.892968	3.948463	2.031223
C	5.859874	1.274976	0.581676	H	5.37175	4.331057	1.248283
C	6.304873	2.544175	0.525607	H	5.435091	3.024066	2.446792
C	5.976387	3.503381	1.63193	H	6.789866	4.138217	-0.81083
C	7.123377	3.121513	-0.58902	H	8.169503	3.195289	-0.27214
C	0.732783	-0.09301	1.460122	H	7.096331	2.510414	-1.4854
O	-0.97303	-3.37502	0.521675	H	0.255946	-0.0466	2.438154
O	-2.84569	-2.35729	-0.82511	H	1.796781	0.067376	1.620626
O	-6.06651	2.368355	-0.37719	H	0.378711	0.726602	0.852087
C	-5.28931	0.898518	1.996283	H	-0.28018	-3.63046	-0.1098
C	-3.65485	2.548221	1.114082	H	-2.50451	-3.20786	-0.50232
C	-7.25974	2.196952	-1.11847	H	-6.11981	1.603915	1.970412
O	6.929711	0.243011	-1.28596	H	-5.69495	-0.11382	1.922336
H	-3.98216	-0.87481	0.569507	H	-4.79972	0.997885	2.965225
H	-3.9953	-0.79368	-1.91197	H	-4.41856	3.293104	1.337707
H	-2.8113	0.192103	-2.74646	H	-2.99292	2.477484	1.978598
H	-4.98245	1.331962	-2.59628	H	-3.07187	2.918146	0.274463
H	-3.77595	2.256982	-1.73384	H	-7.87016	3.084744	-0.95522
H	-5.77039	0.330431	-0.48545	H	-7.07104	2.095745	-2.19318
H	-3.10639	-0.22232	2.819254	H	-7.81525	1.314534	-0.77689
**NImag**						0	
**Σ Electronic and thermal Free Energy**						-924305.616342793	
**mol Fraction**						0.4331	

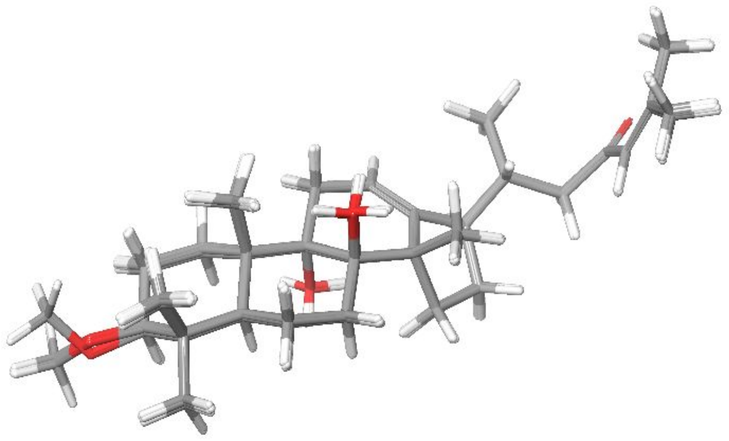

S66: Overlaid 3D structures, cartesian coordinates, number of imaginary frequencies, and energy for Sarasinoside R (8) trans-8*R,9*S** aglycone conformers.**

trans-8*R,9*S** aglycone - Conformer 1**

Atom	X	Y	Z	Atom	X	Y	Z
C	-5.21975	0.802416	0.244642	H	-3.01272	2.534923	-0.71693
C	-3.66197	0.698121	0.24869	H	-1.4684	1.513873	1.703565
C	-3.00507	-0.25484	-0.80164	H	-0.98112	2.855634	0.685772
C	-3.70294	-1.63293	-0.72177	H	1.111339	-2.561	-0.87588
C	-5.22535	-1.53535	-0.80164	H	-0.61997	-0.8427	-2.30712
C	-5.77984	-0.63493	0.292463	H	-1.19723	-2.27382	-1.46708
C	-2.952	2.054816	0.26088	H	2.446412	-1.98054	1.221319
C	-1.49388	1.887863	0.680996	H	3.119972	0.781345	2.071849
C	-0.72181	0.922875	-0.21779	H	2.367703	-0.47885	3.024009
C	-1.49154	-0.42863	-0.37132	H	0.167668	0.146386	2.334928
C	0.753234	0.707104	0.310619	H	0.934553	1.728349	2.263744
C	1.317772	-0.66535	-0.01416	H	-2.47133	-0.30924	-2.91243
C	0.685186	-1.5693	-0.75888	H	-4.14872	0.071541	-2.61903
C	-0.68334	-1.3223	-1.33113	H	-2.90791	1.294411	-2.36858
C	2.521797	-0.94694	0.870431	H	4.053226	0.230183	-0.08459
C	2.323566	0.034839	2.063755	H	4.906162	-2.24749	1.48808
C	0.956507	0.719328	1.849421	H	4.887841	-0.59357	2.119314
C	-3.13982	0.241902	-2.25346	H	3.894931	-2.72783	-0.83518
C	3.907527	-0.81576	0.196824	H	3.283167	-1.3837	-1.80389
C	5.005073	-1.19545	1.213543	H	5.017041	-1.55174	-1.52113
C	4.031772	-1.6675	-1.06552	H	6.074041	1.16079	0.825495
C	6.40671	-0.97386	0.682406	H	9.711529	0.022607	-0.50735
C	6.752323	0.428494	0.398512	H	8.442638	-1.01086	-1.15103
C	7.768872	0.883373	-0.35774	H	8.987887	0.434048	-2.05328
C	8.768947	0.020617	-1.06576	H	8.972788	2.651166	-0.26114
C	7.958157	2.359612	-0.54968	H	7.849545	2.621367	-1.60676
C	1.66142	1.807358	-0.28421	H	7.246358	2.945938	0.030246
O	-0.66463	1.449427	-1.55729	H	1.238964	2.7966	-0.09088
O	-1.53728	-1.0537	0.928421	H	2.646081	1.790601	0.176708
O	-7.21144	-0.57902	0.240875	H	1.786187	1.685947	-1.35776
C	-5.67016	1.504919	1.540509	H	-0.52438	2.405123	-1.51718
C	-5.80435	1.593909	-0.93528	H	-0.68692	-1.49359	1.070908
C	-7.84934	-1.66299	0.889071	H	-6.74867	1.3996	1.658179
O	7.171532	-1.91883	0.51319	H	-5.18391	1.064822	2.414952
H	-3.43515	0.231694	1.211777	H	-5.44035	2.570472	1.525496
H	-3.42834	-2.11564	0.21571	H	-6.87266	1.746042	-0.78079
H	-3.34477	-2.27595	-1.52892	H	-5.33188	2.57451	-1.00772
H	-5.65101	-2.53648	-0.69807	H	-5.67926	1.093616	-1.89164
H	-5.55567	-1.16126	-1.77259	H	-8.92363	-1.49697	0.812335
H	-5.48701	-1.05659	1.265752	H	-7.61074	-2.62554	0.422589
H	-3.43543	2.730584	0.966529	H	-7.57079	-1.71048	1.949352
**NImag**						0	
**Σ Electronic and thermal Free Energy**						-924310.321408206	
**mol Fraction**						0.2271	

trans-8*R,9*S** aglycone - Conformer 2**

Atom	X	Y	Z	Atom	X	Y	Z
C	-5.20699	0.850605	0.219391	H	-2.96175	2.507055	-0.79658
C	-3.65109	0.717849	0.231748	H	-1.44003	1.536469	1.65421
C	-3.01029	-0.28522	-0.78116	H	-0.929	2.836257	0.585625
C	-3.73454	-1.64584	-0.6568	H	1.085064	-2.65439	-0.77741
C	-5.25441	-1.52157	-0.74525	H	-0.68554	-1.07216	-2.27546
C	-5.79399	-0.5735	0.3156	H	-1.24311	-2.39408	-1.28998
C	-2.91445	2.060036	0.196671	H	2.467275	-2.01025	1.241651
C	-1.45933	1.88197	0.621997	H	3.1417	0.758586	2.049306
C	-0.70682	0.877086	-0.2456	H	2.370347	-0.47816	3.016708
C	-1.50155	-0.4707	-0.3402	H	0.182822	0.170383	2.32538
C	0.766664	0.662925	0.281338	H	0.969872	1.738639	2.200881
C	1.314023	-0.72549	-0.00118	H	-2.49293	-0.44116	-2.89268
C	0.662965	-1.65767	-0.69369	H	-4.14544	0.02768	-2.60682
C	-0.71901	-1.43948	-1.24709	H	-2.85141	1.202826	-2.39975
C	2.530447	-0.98252	0.872212	H	4.045173	0.193002	-0.10999
C	2.336959	0.021275	2.048601	H	4.943953	-2.27994	1.442875
C	0.976936	0.716966	1.819011	H	4.893922	-0.63598	2.098563
C	-3.12912	0.165841	-2.24922	H	3.879147	-2.7691	-0.84201
C	3.906429	-0.85145	0.179107	H	3.260741	-1.42967	-1.81241
C	5.018392	-1.2221	1.183587	H	4.997165	-1.59969	-1.54539
C	4.016153	-1.71043	-1.07961	H	6.03019	1.160955	0.847623
C	6.410527	-0.96035	0.645189	H	9.710844	0.146444	-0.45753
C	6.729023	0.456829	0.406693	H	8.477869	-0.89376	-1.15842
C	7.746965	0.958037	-0.31801	H	9.007239	0.591366	-2.0033
C	8.77918	0.141247	-1.03406	H	8.908401	2.748137	-0.14379
C	7.905699	2.443518	-0.45927	H	7.809143	2.736537	-1.5093
C	1.682845	1.743455	-0.34247	H	7.170952	2.994907	0.126245
O	-0.66348	1.482549	-1.55096	H	1.229282	2.728028	-0.23322
O	-1.55972	-1.04001	0.985443	H	2.648257	1.763891	0.15822
O	-7.2242	-0.49393	0.258419	H	1.862901	1.571704	-1.40236
C	-5.64608	1.605777	1.489331	H	-0.17371	0.916983	-2.16319
C	-5.77543	1.612506	-0.98779	H	-0.71871	-1.48921	1.151193
C	-7.88226	-1.54224	0.944286	H	-6.72647	1.524095	1.608665
O	7.187897	-1.88673	0.435053	H	-5.16874	1.187864	2.379404
H	-3.43618	0.28165	1.211487	H	-5.3968	2.665695	1.437292
H	-3.4717	-2.10103	0.297866	H	-6.83898	1.797596	-0.83628
H	-3.38757	-2.32336	-1.44041	H	-5.27801	2.576981	-1.09824
H	-5.69994	-2.51005	-0.6092	H	-5.66781	1.073824	-1.9254
H	-5.57408	-1.17463	-1.72976	H	-8.95333	-1.36084	0.858154
H	-5.51062	-0.96651	1.303552	H	-7.65903	-2.52528	0.514373
H	-3.38545	2.767583	0.879456	H	-7.60712	-1.55517	2.006415
**NImag**						0	
**Σ Electronic and thermal Free Energy**						-924310.047186603	
**mol Fraction**						0.1429	

trans-8*R,9*S** aglycone - Conformer 3**

Atom	X	Y	Z	Atom	X	Y	Z
C	-5.20167	0.912651	0.155638	H	-2.8807	2.46227	-0.8708
C	-3.64916	0.738591	0.204835	H	-1.42973	1.553448	1.650296
C	-3.01523	-0.32998	-0.74375	H	-0.87212	2.787551	0.541298
C	-3.77856	-1.66265	-0.56861	H	1.085068	-2.73531	-0.73148
C	-5.29306	-1.50445	-0.69177	H	-0.76182	-1.24956	-2.19131
C	-5.82742	-0.4903	0.309478	H	-1.26677	-2.50914	-1.10145
C	-2.86926	2.054552	0.13969	H	2.530484	-2.08743	1.198496
C	-1.42749	1.849767	0.599675	H	3.149178	0.668377	2.083123
C	-0.68745	0.794296	-0.21919	H	2.398433	-0.60902	3.013507
C	-1.50783	-0.54057	-0.2732	H	0.202697	0.006981	2.327777
C	0.778192	0.580741	0.312818	H	0.957208	1.604443	2.26615
C	1.331819	-0.79789	0.00488	H	-2.48751	-0.58725	-2.84235
C	0.659374	-1.74002	-0.64487	H	-4.12776	-0.06261	-2.59194
C	-0.74586	-1.55246	-1.14259	H	-2.80353	1.084294	-2.42111
C	2.565548	-1.04948	0.854798	H	4.045254	0.184261	-0.11106
C	2.356698	-0.08212	2.060209	H	5.018519	-2.30627	1.366132
C	0.982953	0.595552	1.850752	H	4.933052	-0.68316	2.068951
C	-3.11126	0.062629	-2.23069	H	3.93521	-2.76213	-0.91112
C	3.931532	-0.86927	0.154284	H	3.266882	-1.41658	-1.83773
C	5.063394	-1.23991	1.13657	H	5.013145	-1.54992	-1.60572
C	4.043193	-1.69523	-1.12617	H	6.011168	1.176313	0.869273
C	6.441735	-0.92682	0.591699	H	9.710018	0.307375	-0.49279
C	6.724086	0.505492	0.399955	H	8.500307	-0.74617	-1.21503
C	7.725033	1.057173	-0.31156	H	8.979486	0.778801	-2.01785
C	8.773413	0.292604	-1.06112	H	8.842462	2.868748	-0.07867
C	7.846127	2.550341	-0.40066	H	7.73693	2.878004	-1.43915
C	1.68568	1.685109	-0.28418	H	7.100825	3.062006	0.207181
O	-0.63235	1.346356	-1.5487	H	1.218726	2.663149	-0.17068
O	-1.49599	-1.14118	1.037747	H	2.642932	1.715332	0.231006
O	-7.2524	-0.37517	0.220196	H	1.88443	1.529657	-1.34325
C	-5.64301	1.741021	1.37831	H	-0.11523	0.764221	-2.12204
C	-5.72595	1.630482	-1.09744	H	-2.0253	-0.60571	1.64425
C	-7.95262	-1.37139	0.941955	H	-6.72686	1.69044	1.480227
O	7.238164	-1.82617	0.338895	H	-5.19325	1.357415	2.297874
H	-3.4965	0.352184	1.222365	H	-5.36719	2.790751	1.277963
H	-3.54643	-2.08473	0.40984	H	-6.78267	1.863765	-0.96737
H	-3.43315	-2.38508	-1.3113	H	-5.19048	2.568038	-1.25243
H	-5.76661	-2.47293	-0.51398	H	-5.63159	1.038746	-2.00362
H	-5.58571	-1.20083	-1.69858	H	-9.01608	-1.1635	0.827392
H	-5.57304	-0.84082	1.321341	H	-7.75073	-2.37921	0.562109
H	-3.33284	2.801165	0.784357	H	-7.69614	-1.34184	2.00832
**NImag**						0	
**Σ Electronic and thermal Free Energy**						-924308.332830946	
**mol Fraction**						0.0079	

trans-8*R,9*S** aglycone - Conformer 4**

Atom	X	Y	Z	Atom	X	Y	Z
C	-5.21966	0.802724	0.244553	H	-3.01254	2.534814	-0.71738
C	-3.66188	0.698267	0.248646	H	-1.46811	1.514247	1.703233
C	-3.00509	-0.25502	-0.80146	H	-0.98077	2.855655	0.685016
C	-3.7031	-1.63301	-0.72121	H	1.111095	-2.56164	-0.87521
C	-5.2255	-1.53533	-0.8011	H	-0.62006	-0.84351	-2.30687
C	-5.77991	-0.63456	0.292752	H	-1.1975	-2.27435	-1.46648
C	-2.95179	2.054901	0.260516	H	2.446405	-1.98061	1.221739
C	-1.49365	1.887933	0.68056	H	3.120236	0.781496	2.071302
C	-0.72174	0.922625	-0.21803	H	2.367937	-0.47836	3.023928
C	-1.49158	-0.42886	-0.37113	H	0.167931	0.146753	2.334842
C	0.753343	0.706864	0.310315	H	0.934826	1.728682	2.263123
C	1.317741	-0.66574	-0.01408	H	-2.47102	-0.30957	-2.9122
C	0.685044	-1.56986	-0.75849	H	-4.14862	0.070517	-2.61909
C	-0.68348	-1.32286	-1.33076	H	-2.90839	1.293968	-2.36871
C	2.521814	-0.94714	0.870484	H	4.0532	0.229577	-0.08509
C	2.323778	0.035056	2.063534	H	4.906428	-2.24767	1.488091
C	0.956737	0.719539	1.849089	H	4.887849	-0.59366	2.119081
C	-3.13984	0.241372	-2.25342	H	3.89467	-2.72866	-0.83485
C	3.907497	-0.81628	0.196673	H	3.28283	-1.38478	-1.80379
C	5.00515	-1.19565	1.213399	H	5.016733	-1.55278	-1.52122
C	4.031533	-1.66838	-1.06542	H	6.073627	1.160666	0.825475
C	6.40669	-0.97387	0.682107	H	9.712229	0.025056	-0.50775
C	6.752135	0.428586	0.398477	H	8.443861	-1.01027	-1.14974
C	7.768783	0.883881	-0.35739	H	8.987006	0.434518	-2.05343
C	8.769224	0.021574	-1.06543	H	8.972276	2.651924	-0.26003
C	7.957789	2.360223	-0.54891	H	7.849359	2.622222	-1.60595
C	1.661583	1.806831	-0.28494	H	7.245713	2.946239	0.030991
O	-0.6646	1.448819	-1.55765	H	1.239304	2.796198	-0.09181
O	-1.53734	-1.05359	0.928726	H	2.64632	1.79007	0.175825
O	-7.2115	-0.57849	0.241158	H	1.786128	1.685146	-1.35847
C	-5.67005	1.505608	1.54022	H	-0.5246	2.404576	-1.51783
C	-5.80413	1.593949	-0.93561	H	-0.68696	-1.49337	1.071382
C	-7.84956	-1.66241	0.889275	H	-6.74857	1.400418	1.657884
O	7.171537	-1.91878	0.51258	H	-5.18388	1.065677	2.414793
H	-3.43511	0.232044	1.211845	H	-5.44015	2.571135	1.524967
H	-3.42854	-2.11549	0.216407	H	-6.87248	1.746015	-0.78132
H	-3.345	-2.27633	-1.52817	H	-5.33173	2.574581	-1.00812
H	-5.65122	-2.5364	-0.69724	H	-5.67881	1.093505	-1.89186
H	-5.55581	-1.16148	-1.77215	H	-8.92382	-1.49588	0.813126
H	-5.48714	-1.05599	1.266159	H	-7.61162	-2.6249	0.422337
H	-3.4351	2.730849	0.966063	H	-7.57053	-1.7104	1.949404
**NImag**						0	
**Σ Electronic and thermal Free Energy**						-924310.328938319	
**mol Fraction**						0.2300	

trans-8*R**,9*S** aglycone - Conformer 5**

Atom	X	Y	Z	Atom	X	Y	Z
C	-5.21345	0.875291	0.184512	H	-2.92774	2.490089	-0.81295
C	-3.65828	0.72543	0.218439	H	-1.4495	1.536659	1.680293
C	-3.01353	-0.30598	-0.76437	H	-0.91435	2.809604	0.612646
C	-3.75297	-1.65597	-0.6166	H	1.09812	-2.66394	-0.81283
C	-5.27115	-1.52155	-0.72221	H	-0.69387	-1.04451	-2.24261
C	-5.81419	-0.54169	0.307967	H	-1.23438	-2.41125	-1.28285
C	-2.90094	2.055285	0.18671	H	2.500467	-2.05646	1.189977
C	-1.45481	1.858411	0.637031	H	3.136042	0.702172	2.072251
C	-0.70003	0.835613	-0.21447	H	2.398845	-0.57849	3.008792
C	-1.49824	-0.50492	-0.31358	H	0.193768	0.016988	2.319266
C	0.768866	0.6208	0.314245	H	0.937358	1.620106	2.282987
C	1.333107	-0.74814	-0.01689	H	-2.48028	-0.48654	-2.86737
C	0.674625	-1.66937	-0.70843	H	-4.14358	-0.03646	-2.60116
C	-0.71586	-1.45349	-1.23371	H	-2.86366	1.156437	-2.40799
C	2.553315	-1.01832	0.848455	H	4.053798	0.194513	-0.1116
C	2.347595	-0.05303	2.05507	H	4.984631	-2.28439	1.413098
C	0.971092	0.617708	1.852072	H	4.918042	-0.64665	2.083105
C	-3.13085	0.119593	-2.24056	H	3.939763	-2.75992	-0.88096
C	3.929673	-0.85573	0.163648	H	3.283333	-1.42259	-1.82834
C	5.047602	-1.22356	1.16298	H	5.027099	-1.55767	-1.57825
C	4.052105	-1.69565	-1.10672	H	6.033078	1.172747	0.846702
C	6.435364	-0.94267	0.624765	H	9.724096	0.216269	-0.47771
C	6.738771	0.480315	0.398313	H	8.501831	-0.83752	-1.17775
C	7.749524	0.999304	-0.32372	H	9.006126	0.65925	-2.01732
C	8.789493	0.200823	-1.04929	H	8.891013	2.801146	-0.13564
C	7.891357	2.487779	-0.45224	H	7.789666	2.788951	-1.49949
C	1.670516	1.732207	-0.27212	H	7.151347	3.025656	0.139223
O	-0.64067	1.304055	-1.57645	H	1.237079	2.719019	-0.08959
O	-1.46668	-1.14945	0.974751	H	2.649096	1.729596	0.201367
O	-7.24158	-0.44871	0.232829	H	1.814292	1.607584	-1.34338
C	-5.66033	1.665483	1.430219	H	-0.45373	2.252386	-1.57796
C	-5.75751	1.614762	-1.0473	H	-2.0262	-0.66282	1.595151
C	-7.91887	-1.47507	0.934017	H	-6.74238	1.593472	1.538024
O	7.222709	-1.85831	0.403891	H	-5.19762	1.26732	2.337074
H	-3.49173	0.314364	1.224296	H	-5.40375	2.722146	1.354163
H	-3.50538	-2.10031	0.347938	H	-6.81896	1.819956	-0.90863
H	-3.40122	-2.35245	-1.38058	H	-5.24448	2.568614	-1.17734
H	-5.72582	-2.50237	-0.56364	H	-5.65085	1.050516	-1.96941
H	-5.57908	-1.19894	-1.71844	H	-8.98661	-1.2828	0.832828
H	-5.54564	-0.91238	1.308967	H	-7.70269	-2.46917	0.526945
H	-3.37319	2.777514	0.852254	H	-7.65445	-1.46825	1.998839
**NImag**						0	
**Σ Electronic and thermal Free Energy**						-924308.418172224	
**mol Fraction**						0.0091	

trans-8*R,9*S** aglycone - Conformer 6**

Atom	X	Y	Z	Atom	X	Y	Z
C	-5.20705	0.850414	0.219433	H	-2.96183	2.507307	-0.79586
C	-3.65114	0.717735	0.231863	H	-1.44018	1.536115	1.654731
C	-3.01023	-0.28502	-0.78127	H	-0.92916	2.836181	0.586489
C	-3.73442	-1.64571	-0.65732	H	1.085232	-2.65404	-0.77786
C	-5.25428	-1.52149	-0.74588	H	-0.68533	-1.07154	-2.27563
C	-5.79398	-0.57375	0.315198	H	-1.24292	-2.39367	-1.29048
C	-2.91457	2.059978	0.19725	H	2.467251	-2.01028	1.241633
C	-1.45945	1.881868	0.622603	H	3.141603	0.758488	2.049633
C	-0.70686	0.877236	-0.24523	H	2.370404	-0.47844	3.01692
C	-1.5015	-0.47054	-0.34026	H	0.182777	0.16998	2.325638
C	0.766627	0.662993	0.281726	H	0.969708	1.738331	2.20152
C	1.314055	-0.72534	-0.00105	H	-2.49279	-0.44045	-2.8928
C	0.663099	-1.65735	-0.69388	H	-4.14527	0.028428	-2.60691
C	-0.71884	-1.43906	-1.24733	H	-2.85118	1.203437	-2.39944
C	2.530437	-0.9825	0.872329	H	4.045092	0.193132	-0.10982
C	2.336924	0.021113	2.048878	H	4.943956	-2.27974	1.443112
C	0.976859	0.716748	1.819411	H	4.894016	-0.63572	2.098662
C	-3.12896	0.166423	-2.24921	H	3.879393	-2.76898	-0.84185
C	3.906435	-0.85134	0.17926	H	3.260752	-1.42964	-1.81224
C	5.018423	-1.22192	1.183731	H	4.997208	-1.5994	-1.54529
C	4.016231	-1.7103	-1.07947	H	6.030238	1.161081	0.847248
C	6.410573	-0.96027	0.645308	H	9.710589	0.145711	-0.45766
C	6.72904	0.456847	0.406447	H	8.477495	-0.89414	-1.1588
C	7.746906	0.957841	-0.31852	H	9.007425	0.590844	-2.00358
C	8.779045	0.140797	-1.03439	H	8.908379	2.747979	-0.14493
C	7.905644	2.443274	-0.46022	H	7.809007	2.735984	-1.51034
C	1.682807	1.743631	-0.3419	H	7.170957	2.99485	0.125187
O	-0.66342	1.483144	-1.55039	H	1.229317	2.728202	-0.23233
O	-1.55975	-1.04017	0.985236	H	2.648277	1.763863	0.158688
O	-7.2242	-0.49428	0.257925	H	1.862689	1.572123	-1.40185
C	-5.64628	1.605193	1.489572	H	-0.17436	0.917379	-2.163
C	-5.77547	1.612631	-0.98756	H	-0.71873	-1.48934	1.150954
C	-7.88221	-1.54267	0.943745	H	-6.72667	1.523376	1.608813
O	7.187982	-1.88668	0.435448	H	-5.16894	1.187075	2.379552
H	-3.43629	0.281269	1.211499	H	-5.3971	2.665149	1.437838
H	-3.47164	-2.10112	0.297264	H	-6.83903	1.797677	-0.83601
H	-3.38733	-2.32303	-1.44106	H	-5.27806	2.577138	-1.09774
H	-5.69978	-2.51004	-0.61016	H	-5.66786	1.074199	-1.92531
H	-5.57387	-1.17428	-1.73032	H	-8.95331	-1.36157	0.857232
H	-5.51066	-0.96702	1.303056	H	-7.65857	-2.52571	0.514052
H	-3.38565	2.767276	0.880235	H	-7.60741	-1.55534	2.005964

**NImag**	0
**Σ Electronic and thermal Free Energy**	-924310.052206678
**mol Fraction**	0.1441

trans-8*R,9*S** aglycone - Conformer 7**

Atom	X	Y	Z	Atom	X	Y	Z
C	-5.21356	0.875085	0.184618	H	-2.92794	2.4902	-0.81256
C	-3.65838	0.725325	0.218524	H	-1.44973	1.536531	1.680609
C	-3.01354	-0.30587	-0.76445	H	-0.91462	2.809652	0.613155
C	-3.75288	-1.65593	-0.61692	H	1.098352	-2.66344	-0.81347
C	-5.27107	-1.52159	-0.72257	H	-0.69386	-1.04397	-2.24282
C	-5.81421	-0.54196	0.307768	H	-1.23418	-2.4109	-1.28326
C	-2.90115	2.055247	0.187037	H	2.500453	-2.0563	1.189801
C	-1.45502	1.858422	0.637388	H	3.135877	0.702207	2.072562
C	-0.70013	0.835809	-0.21427	H	2.39872	-0.57869	3.008815
C	-1.49824	-0.50478	-0.31367	H	0.193649	0.016857	2.319414
C	0.768748	0.620978	0.314459	H	0.937154	1.620005	2.283327
C	1.33311	-0.74784	-0.01694	H	-2.48047	-0.48627	-2.86751
C	0.674755	-1.66894	-0.70878	H	-4.14366	-0.03588	-2.60113
C	-0.71577	-1.45309	-1.23398	H	-2.86352	1.156746	-2.4079
C	2.553289	-1.01811	0.848428	H	4.053797	0.194869	-0.11143
C	2.347477	-0.05303	2.055205	H	4.984405	-2.28416	1.413223
C	0.970937	0.617666	1.852278	H	4.918129	-0.64639	2.08314
C	-3.13088	0.11992	-2.24058	H	3.939842	-2.75949	-0.88108
C	3.929687	-0.85541	0.163714	H	3.283435	-1.42208	-1.82836
C	5.047548	-1.22335	1.16302	H	5.027179	-1.55715	-1.57822
C	4.052172	-1.6952	-1.10673	H	6.033268	1.172671	0.846129
C	6.435417	-0.9428	0.624894	H	9.725288	0.218306	-0.47903
C	6.739005	0.480089	0.398037	H	8.503413	-0.83857	-1.17544
C	7.749865	0.998916	-0.32396	H	9.004669	0.657267	-2.01847
C	8.789923	0.200355	-1.04929	H	8.891258	2.8009	-0.13636
C	7.89163	2.487374	-0.45287	H	7.789897	2.788251	-1.50021
C	1.670377	1.732539	-0.27167	H	7.151573	3.025363	0.138427
O	-0.64075	1.304524	-1.57614	H	1.236895	2.7193	-0.08897
O	-1.46667	-1.14959	0.974513	H	2.648942	1.729886	0.201845
O	-7.2416	-0.44906	0.232554	H	1.81424	1.60812	-1.34294
C	-5.66049	1.664989	1.430489	H	-0.45366	2.252814	-1.57748
C	-5.75767	1.614789	-1.04703	H	-2.02592	-0.66291	1.595119
C	-7.91887	-1.47549	0.933661	H	-6.74253	1.592844	1.538306
O	7.222676	-1.85864	0.404534	H	-5.19771	1.266696	2.337255
H	-3.49179	0.314079	1.2243	H	-5.40402	2.721694	1.354637
H	-3.50529	-2.10039	0.347568	H	-6.8191	1.820017	-0.90824
H	-3.40105	-2.35227	-1.38099	H	-5.24461	2.568635	-1.17697
H	-5.72568	-2.50247	-0.56419	H	-5.65113	1.050685	-1.96924
H	-5.57898	-1.19882	-1.71875	H	-8.98661	-1.28326	0.832466
H	-5.54569	-0.91283	1.308709	H	-7.70265	-2.46956	0.52652
H	-3.37349	2.777339	0.852663	H	-7.65446	-1.46875	1.998487
**NImag**						0	
**Σ Electronic and thermal Free Energy**						-924308.430722411	
**mol Fraction**						0.0093	

trans-8*R,9*S** aglycone - Conformer 8**

Atom	X	Y	Z	Atom	X	Y	Z
C	-5.21961	0.802718	0.244621	H	-3.01247	2.534686	-0.71765
C	-3.66184	0.698272	0.248605	H	-1.4681	1.51431	1.703099
C	-3.00509	-0.25506	-0.80148	H	-0.98077	2.855694	0.684828
C	-3.70309	-1.63304	-0.7212	H	1.111117	-2.56156	-0.87549
C	-5.2255	-1.53532	-0.80107	H	-0.62021	-0.84354	-2.30698
C	-5.77986	-0.63455	0.292816	H	-1.19748	-2.27438	-1.46646
C	-2.95175	2.054908	0.260319	H	2.446416	-1.98069	1.221513
C	-1.49362	1.887971	0.680413	H	3.120175	0.781319	2.071418
C	-0.7217	0.92261	-0.2181	H	2.36784	-0.47862	3.023873
C	-1.49155	-0.42886	-0.37123	H	0.167834	0.14666	2.334768
C	0.753344	0.706845	0.310296	H	0.934839	1.728547	2.263165
C	1.317769	-0.66573	-0.0142	H	-2.47162	-0.31032	-2.9123
C	0.685054	-1.56979	-0.75865	H	-4.14885	0.07112	-2.61885
C	-0.68351	-1.32285	-1.33084	H	-2.9076	1.293663	-2.36892
C	2.521831	-0.94718	0.870388	H	4.053259	0.229707	-0.0849
C	2.323709	0.034875	2.063513	H	4.906372	-2.24787	1.487818
C	0.956691	0.719426	1.849083	H	4.887783	-0.59401	2.119216
C	-3.13986	0.241275	-2.25344	H	3.894696	-2.7284	-0.83504
C	3.907514	-0.81619	0.196686	H	3.283032	-1.38435	-1.80393
C	5.005115	-1.19579	1.213391	H	5.016899	-1.55253	-1.5212
C	4.031635	-1.66812	-1.06554	H	6.073729	1.160684	0.826054
C	6.406696	-0.97388	0.682227	H	9.711402	0.023551	-0.50719
C	6.752109	0.428637	0.3988	H	8.442688	-1.00996	-1.15114
C	7.768586	0.883916	-0.3573	H	8.987657	0.435293	-2.05299
C	8.768809	0.021545	-1.06558	H	8.972262	2.651857	-0.26018
C	7.957656	2.360247	-0.54875	H	7.848939	2.622332	-1.60574
C	1.661586	1.806899	-0.28485	H	7.245807	2.946279	0.031406
O	-0.66447	1.448746	-1.55778	H	1.239204	2.796213	-0.09173
O	-1.53726	-1.0536	0.928703	H	2.646268	1.790175	0.176026
O	-7.21145	-0.57844	0.241257	H	1.78628	1.685222	-1.35837
C	-5.66987	1.505584	1.540345	H	-0.52426	2.404459	-1.51798
C	-5.80419	1.593987	-0.93545	H	-0.687	-1.49366	1.071136
C	-7.84952	-1.66234	0.889413	H	-6.74838	1.400393	1.658115
O	7.171607	-1.91869	0.512593	H	-5.1836	1.065657	2.414861
H	-3.43499	0.23211	1.211812	H	-5.43997	2.571111	1.52505
H	-3.42855	-2.11552	0.216429	H	-6.87246	1.746339	-0.78092
H	-3.345	-2.27635	-1.52816	H	-5.33157	2.574492	-1.00821
H	-5.65125	-2.53638	-0.69722	H	-5.67928	1.093415	-1.8917
H	-5.55579	-1.16146	-1.77212	H	-8.92377	-1.49585	0.813187
H	-5.48707	-1.056	1.266205	H	-7.61152	-2.62485	0.422552
H	-3.43508	2.730961	0.965758	H	-7.57054	-1.71025	1.949559
**NImag**					0		
**Σ Electronic and thermal Free Energy**					-924310.326428282		
**mol Fraction**					0.2290		

trans-8*R**,9*S*** **aglycone - Conformer 9**

Atom	X	Y	Z	Atom	X	Y	Z
C	-5.22432	0.76094	0.312469	H	-3.05206	2.377258	-0.9015
C	-3.66407	0.674226	0.304023	H	-1.45083	1.685844	1.596915
C	-3.01591	-0.40078	-0.62573	H	-1.00164	2.895689	0.409763
C	-3.69111	-1.76316	-0.34375	H	1.127786	-2.65168	-0.50418
C	-5.21657	-1.70367	-0.41643	H	-0.65625	-1.14598	-2.10301
C	-5.76215	-0.67232	0.55884	H	-1.19514	-2.46586	-1.07611
C	-2.96939	2.027632	0.12868	H	2.499191	-1.79254	1.471472
C	-1.50175	1.929646	0.536746	H	3.1568	1.063694	1.93889
C	-0.73692	0.868629	-0.25318	H	2.445725	-0.07016	3.065618
C	-1.49007	-0.49986	-0.21488	H	0.22054	0.433866	2.354306
C	0.753365	0.737567	0.261497	H	0.963179	2.002222	2.063561
C	1.329071	-0.65901	0.101923	H	-2.54274	-0.73942	-2.72415
C	0.691998	-1.6573	-0.50581	H	-4.21077	-0.31512	-2.4381
C	-0.69161	-1.49924	-1.07314	H	-2.96251	0.924723	-2.38649
C	2.555311	-0.81273	0.987784	H	4.05187	0.241582	-0.14918
C	2.371406	0.313483	2.048177	H	4.958375	-1.99209	1.729836
C	0.990278	0.948782	1.781077	H	4.948982	-0.2654	2.120148
C	-3.1919	-0.10205	-2.12622	H	3.914096	-2.79164	-0.49452
C	3.925063	-0.75884	0.272203	H	3.265444	-1.59513	-1.62027
C	5.047488	-0.98972	1.306416	H	5.006678	-1.70753	-1.35705
C	4.032892	-1.7705	-0.86766	H	6.090827	1.283004	0.546858
C	6.436748	-0.84692	0.718202	H	9.734054	-0.00428	-0.6237
C	6.771851	0.499975	0.228113	H	8.474233	-1.13864	-1.09313
C	7.781982	0.846207	-0.59188	H	8.996724	0.158369	-2.20861
C	8.788394	-0.10272	-1.16815	H	8.970742	2.618617	-0.76668
C	7.957121	2.279279	-1.00136	H	7.839821	2.380157	-2.08482
C	1.633705	1.761458	-0.49107	H	7.243339	2.939412	-0.50997
O	-0.721	1.223441	-1.6493	H	1.20365	2.763401	-0.41522
O	-1.49623	-0.95338	1.154383	H	2.628996	1.812781	-0.05644
O	-7.19745	-0.70433	0.69873	H	1.735078	1.504512	-1.54302
C	-5.66028	1.615541	1.518479	H	-0.57954	2.176292	-1.73242
C	-5.82661	1.41082	-0.9429	H	-0.6389	-1.36724	1.329247
C	-7.98251	-1.03125	-0.43845	H	-6.73597	1.519925	1.665143
O	7.199771	-1.8073	0.670877	H	-5.15965	1.288241	2.433361
H	-3.4164	0.338385	1.315123	H	-5.43659	2.671954	1.368644
H	-3.39705	-2.10594	0.647461	H	-6.88364	1.624702	-0.77959
H	-3.33469	-2.50776	-1.05904	H	-5.33353	2.360809	-1.15317
H	-5.62269	-2.68564	-0.15981	H	-5.74261	0.79443	-1.83416
H	-5.54584	-1.48875	-1.4341	H	-9.021	-0.91581	-0.12834
H	-5.40765	-0.95512	1.55606	H	-7.799	-0.36871	-1.28732
H	-3.44777	2.784243	0.750779	H	-7.83155	-2.06592	-0.76069
**NImag**						0	
**Σ Electronic and thermal Free Energy**						-924306.719504302	
**mol Fraction**						0.0005	

trans-8*R,9*S** aglycone - Conformer 10**

Atom	X	Y	Z	Atom	X	Y	Z
C	-5.22427	0.774427	0.295915	H	-3.02322	2.375113	-0.89228
C	-3.66448	0.675937	0.305947	H	-1.45138	1.663788	1.614756
C	-3.01448	-0.4056	-0.6151	H	-0.98444	2.873614	0.426535
C	-3.70365	-1.76226	-0.34175	H	1.13042	-2.67614	-0.46116
C	-5.22755	-1.69014	-0.43267	H	-0.68984	-1.26715	-2.07416
C	-5.77627	-0.65436	0.53617	H	-1.20626	-2.50934	-0.96926
C	-2.95646	2.023515	0.137558	H	2.519249	-1.80878	1.478789
C	-1.49273	1.915462	0.556488	H	3.159618	1.044395	1.969588
C	-0.73188	0.853794	-0.23229	H	2.443588	-0.09878	3.083577
C	-1.49423	-0.51439	-0.18773	H	0.222712	0.416259	2.375011
C	0.756741	0.72023	0.281171	H	0.96649	1.983396	2.082514
C	1.330018	-0.67777	0.125503	H	-2.53628	-0.761	-2.71174
C	0.690202	-1.68385	-0.46663	H	-4.18997	-0.28899	-2.43897
C	-0.70254	-1.54413	-1.01742	H	-2.91112	0.918485	-2.37329
C	2.563397	-0.82547	1.001307	H	4.041886	0.24916	-0.14032
C	2.374124	0.293217	2.06904	H	4.977636	-1.98554	1.723261
C	0.993152	0.929768	1.801656	H	4.962411	-0.25984	2.117073
C	-3.16921	-0.10272	-2.1173	H	3.927296	-2.78347	-0.50146
C	3.927096	-0.75459	0.276012	H	3.260021	-1.58702	-1.61615
C	5.058379	-0.98196	1.301027	H	5.003823	-1.68548	-1.36666
C	4.034541	-1.75938	-0.86999	H	6.081862	1.295581	0.526283
C	6.44297	-0.83142	0.703463	H	9.726201	0.025795	-0.65678
C	6.766258	0.515808	0.20679	H	8.471395	-1.11678	-1.11904
C	7.769565	0.86535	-0.62021	H	8.982535	0.180402	-2.23961
C	8.779298	-0.07925	-1.19781	H	8.945534	2.644726	-0.80982
C	7.932757	2.297752	-1.03681	H	7.808214	2.393046	-2.11998
C	1.638769	1.749429	-0.46564	H	7.217603	2.955325	-0.544
O	-0.73134	1.340473	-1.587	H	1.171201	2.73327	-0.44019
O	-1.51691	-0.95833	1.185751	H	2.615649	1.831672	0.005454
O	-7.21326	-0.67567	0.659493	H	1.796588	1.478538	-1.50829
C	-5.6669	1.632641	1.49698	H	-0.23548	0.736543	-2.15634
C	-5.80797	1.429618	-0.96564	H	-0.66264	-1.37066	1.377575
C	-7.98778	-0.99551	-0.48689	H	-6.74482	1.545129	1.631911
O	7.211879	-1.78701	0.654818	H	-5.17865	1.301973	2.41732
H	-3.43212	0.338298	1.319981	H	-5.4334	2.68715	1.349091
H	-3.424	-2.10728	0.65305	H	-6.86374	1.656302	-0.81159
H	-3.34583	-2.51023	-1.05296	H	-5.30134	2.372988	-1.17312
H	-5.64475	-2.66881	-0.18142	H	-5.72486	0.811059	-1.85555
H	-5.54229	-1.47239	-1.45437	H	-9.02884	-0.87305	-0.18835
H	-5.43554	-0.93993	1.537392	H	-7.79004	-0.33323	-1.33274
H	-3.43468	2.781949	0.75782	H	-7.84065	-2.03086	-0.80878
**NImag**					0		
**Σ Electronic and thermal Free Energy**					-924306.212476714		
**mol Fraction**					0.0002		
